# Structural basis of FatB-mediated iron uptake via tyrosine/histidine direct coordination accompanying long-distance domain reorganization

**DOI:** 10.1038/s41467-026-72127-y

**Published:** 2026-04-18

**Authors:** Hyosub Lee, Seong Ok Kim, Seyoung You, Alekos Segalina, Taeyoon Noh, Hyotcherl Ihee

**Affiliations:** 1 Department of Chemistry, Korea Advanced Institute of Science and Technology (KAIST), Daejeon, Republic of Korea; 2 Center for Advanced Reaction Dynamics (CARD), Institute for Basic Science (IBS), Daejeon, Republic of Korea

**Keywords:** X-ray crystallography, SAXS, Coordination chemistry, Computational biophysics, Ion transport

## Abstract

Iron is an essential cofactor for fundamental biological processes. However, Fe(III) is poorly soluble under aerobic conditions, limiting its bioavailability. To secure this essential nutrient, bacteria release high-affinity siderophores that capture environmental Fe(III) and are subsequently imported into the cell as ferric siderophore complexes. While biochemical studies have characterized siderophore uptake in *Bacillus* species, atomic-level mechanisms of recognition and coordination remain unclear. Here, we investigate the siderophore-binding protein FatB from *Bacillus cereus* and its interactions with its siderophore, petrobactin (PB), as well as with ferric petrobactin (FePB) and its ferric photoproduct (FePB^ν^). Crystal structures of apo- and ferric-ligand-bound FatB, supported by biophysical and mutational analyses, reveal that ferric-siderophore binding induces substantial domain closure of FatB. This conformational transition involves an extensive ~29-Å reorganization of a flexible loop, which positions His252 alongside Tyr317 to directly coordinate the Fe(III) center in the FePB-FatB complex. This protein-derived coordination mode is maintained in the FePB^ν^-FatB complex, where a structured water network preserves interfacial complementarity and functional recognition. These findings provide a structural framework for siderophore recognition and iron acquisition and illustrate how active-site coordination and domain reorganization facilitate robust capture of chemically labile ligands, offering insights for antimicrobial development targeting bacterial iron uptake.

## Abstract (web summary)

FatB captures iron-loaded petrobactin by reshaping its binding cleft and coordinating iron, and structural and biophysical analyses reveal how this preserves bacterial iron uptake after photochemical modification of the ligand.

## Introduction

Iron is an essential nutrient for life, serving as a critical cofactor in respiration, DNA synthesis, and redox transformations^1–4^. Under aerobic, neutral pH conditions, Fe(III) is poorly soluble, which limits its bioavailability and prompts bacteria to secrete small, high-affinity iron-chelators^5–9^. Among these, siderophores sequester Fe(III) through multidentate coordination using diverse scaffolds, such as catecholate, hydroxamate, and carboxylate types, and deliver it to specific transport systems for cellular uptake^6,7,9,10^. While Gram-negative bacteria import iron-siderophore complexes via TonB-dependent transporters, Gram-positive bacteria, such as *Bacillus cereus,* lack an outer membrane and instead employ ATP-binding cassette (ABC) transporters to import siderophores through the thick peptidoglycan layer^7,11–13^. These transporters not only ensure high-affinity recognition of target siderophores but also discriminate among chemically similar ligands to maintain efficient iron acquisition under physiologically restrictive conditions^14,15^. In Gram-positive bacteria, this critical function is mediated by substrate-binding proteins (SBPs), which serve as the initial recognition units that capture ligands for delivery to the cognate ABC importer^11,16,17^.

SBPs share a bilobal architecture and are classified by their interdomain linker^18–20^. Many siderophore-binding SBPs belong to class III, characterized by a single, central α-helix that acts as a hinge^21–24^. Unlike the flexible Venus flytrap motion of classes I and II, class-III SBPs undergo a more rigid, hinge-like closure to secure the ligand for delivery to the ABC transporter^21–26^. Despite extensive progress in elucidating siderophore-mediated iron uptake, the structural insight into Gram-positive systems remains limited^11,13,14,27^. Bridging this gap is crucial for developing Trojan horse antibiotics, where siderophore conjugates are hijacked by bacterial uptake systems to deliver antimicrobial payloads^14,28–31^. Such strategies successfully target Gram-negative pathogens, as exemplified by cefiderocol, a siderophore-cephalosporin conjugate that exploits TonB-dependent transporters for cell entry^32–34^. Mechanistically, whereas Gram-negative uptake is initiated by TonB-dependent outer-membrane receptors that select and translocate ferric complexes through a dedicated channel, Gram-positive uptake relies on SBP-mediated capture as the primary extracellular selectivity step followed by handoff to an ABC importer. This SBP-centered architecture imposes distinct constraints and opportunities for stabilizing ferric coordination during recognition and delivery.

In this context, FatB provides an opportunity to address a central mechanistic question: how can an SBP preserve high-affinity uptake while accommodating ferric ligands that are chemically labile and can undergo structural modification? FatB is the cognate class-III SBP for petrobactin (PB), a catecholate-type siderophore widely used by *Bacillus* species^35,36^. Upon light exposure, Fe(III)-bound PB (hereafter denoted as FePB) populates a ligand-to-metal charge-transfer (LMCT) excited state, which subsequently drives oxidative decarboxylation, yielding a photoproduct (PB^ν^)^37^. Remarkably, PB^ν^ remains capable of chelating Fe(III) and reconstitutes the ferric complex (hereafter denoted as FePB^ν^)^38^. Unlike most photoactive siderophores whose photoproducts typically lose their ability to bind the cognate receptor, FatB continues to recognize and bind FePB^ν^ with high affinity, thereby maintaining siderophore-mediated iron uptake even after substantial chemical modifications^36,39^. Thus, understanding how FatB achieves such adaptability, in which effective iron uptake is preserved despite changes in ligand structure induced by photoreaction, provides not only fundamental insight into bacterial iron acquisition but also a framework for developing siderophore-based therapeutic strategies against Gram-positive pathogens^9,15,40^.

We hypothesized that FatB maintains high-affinity uptake across FePB and its photoproduct, FePB^ν^, by coupling ligand-dependent conformational gating with protein- and solvent-mediated stabilization within the binding cleft. In this view, chemical modification of the ferric ligand may be accommodated by compensatory interactions that preserve a transport-compatible complex. To test this hypothesis, we investigated the FatB from *B. cereus* at atomic resolution, focusing on its interactions with FePB, its ferric photoproduct, FePB^ν^, and the minimal catecholate mimic Fe(III)-(3,4-dihydroxybenzoic acid)_2_ (hereafter referred to as [Fe(3,4-DHB)_2_]).

In this work, crystal structures of apo- and ferric-ligand-bound FatB, complemented by small-angle X-ray scattering (SAXS), circular dichroism (CD), site-directed mutagenesis, fluorescence assays, and ab initio quantum-chemical calculations, reveal how FatB establishes a first coordination sphere via Tyr and His residues, undergoes hinge-like domain rearrangements, and leverages protein scaffold flexibility and solvent-mediated interactions to accommodate distinct ligands. Together, these findings establish a mechanistic framework for how Gram-positive siderophore-binding proteins preserve high-affinity iron uptake from chemically labile ligands by dynamically reorganizing their coordination sphere and binding cleft.

## Results

### The apo-FatB structure reveals a canonical class-III SBP fold with a dynamic ligand-binding motif

**Fig. 1:**
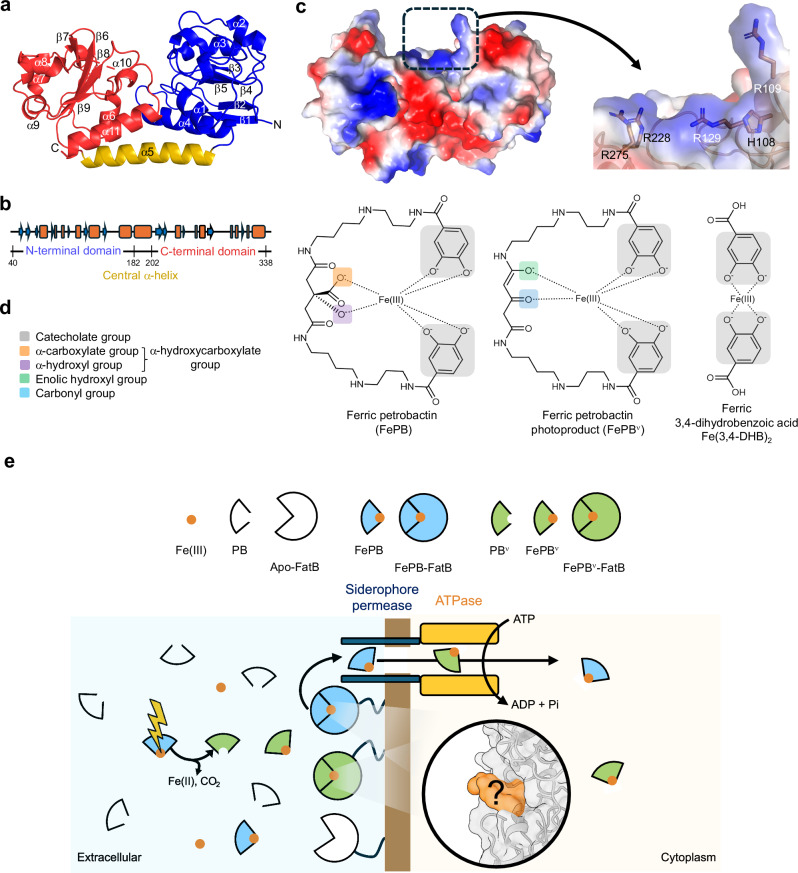
Overall structure of apo-FatB and the chemical landscape of its ferric ligands. **a** Crystal structure of apo-FatB showing the N-terminal domain (blue), central α-helix (yellow), and C-terminal domain (red). **b** Domain organization and secondary-structure schematic with α-helices shown as orange boxes and β-strands as blue arrows. **c** Electrostatic surface potential of apo-FatB (red, negative; blue, positive), highlighting the positively charged interdomain cleft (right). **d** Molecular structures of the ferric ligands used in this study, with Fe(III)-coordinating functional groups highlighted^36,37^. Catechol donors are gray; in FePB (left), the α-hydroxyl and α-carboxyl groups (orange, purple) form an α-hydroxycarboxylate donor, in FePB^ν ^(middle), an enolic hydroxyl and carbonyl (green, blue) form the donor pair, and Fe(3,4-DHB)_2_ (right) contributes only catechol donors. **e** Schematic of FePB/FePB^ν^ photochemistry and FatB-mediated uptake based on previous studies^17,36,37,39,45^. PB chelates Fe(III) to form FePB, which undergoes photodecarboxylation, yielding Fe(II) and PB^ν^; the latter re-sequesters Fe(III) to form FePB^ν^. Both ferric complexes are captured by the cell-surface FatB and proposed to deliver ferric iron to the cytoplasm via an ABC transporter. The question mark highlights the currently unresolved mechanism by which FatB recognizes and binds its ligands, FePB and FePB^ν^, which is elucidated in this work.

To establish a structural basis for understanding the function of FatB, recombinant FatB was expressed in *E. coli* and purified as described in the Methods section (Supplementary Table 1). First, we determined the X-ray crystal structure of FatB in its ligand-free (PDB ID: 21ZD, hereafter referred to as apo-FatB) form. Apo-FatB adopts the bilobal architecture of class-III SBPs, featuring N- and C-terminal α/β domains connected by a central α-helix (Fig. 1a, b). A cluster of basic residues (His108, Arg109, Arg129, Arg228, and Arg275) lines the cleft, creating a strongly positive, solvent-accessible surface well-suited for accommodating anionic siderophores (Fig. 1c and Supplementary Fig. 1). The overall electrostatic surface reveals a domain-level asymmetry: the C-terminal domain presents an extended, positively charged patch, previously proposed as an interaction surface for transport partners, while the N-terminal domain displays a heterogeneous electrostatic potential with mixed positive and negative patches suited for siderophore recognition^35^.

To compare apo-FatB with known siderophore-binding proteins for identifying features unique to its architecture, we performed a structural homology search using the DALI server^41^. This search identified the closest structural homologs of FatB: (i) *Parageobacillus thermoglucosidasius* Pth (PDB ID: 8BNW, hereafter apo-Pth), and (ii) *Bacillus subtilis* YclQ (PDB ID: 3GFV, hereafter apo-YclQ)^35,42^. Consistent with the trends in SBPs, both homologs exhibit remarkable structural similarity with apo-FatB, characterized by an identical Z-score of 25.5 despite low sequence identity (~30%) (Supplementary Fig. 2)^41,43^. Superposition of the entire structures results in root-mean-square deviation (RMSD) values of 2.3 Å for apo-Pth and 2.5 Å for apo-YclQ. Structural alignment of the individual domains shows higher N-terminal conservation, with RMSDs of 1.3 Å for the N-terminal domains in both apo-Pth and apo-YclQ, compared to 1.4 and 1.5 Å for the C-terminal domains, respectively (Supplementary Fig. 3).

This discrepancy between global and individual-domain RMSDs highlights a pronounced deviation in the relative orientation of the two domains in apo-FatB. To quantify this divergence, we calculated the angle separating the centers of mass (COMs) of the C-terminal domains, using the COM of the superimposed N-terminal domain as a reference. While the homologs share a similar orientation (differing by only 2.3°), the C-terminal domain of apo-FatB is displaced significantly outward, resulting in large hinge angle differences of 10.9° and 13.2° relative to apo-Pth and apo-YclQ, respectively.

Several structural features help explain this altered orientation. The central α-helix in apo-FatB (20 residues, 30.4 Å) is approximately one α-helical turn shorter than the corresponding helices in apo-Pth (24 residues, 35.0 Å) and apo-YclQ (25 residues, 36.5 Å), indicating a more compact hinge between the domains. Beyond the hinge, the C-terminal domain of apo-FatB exhibits high intrinsic flexibility: it features a missing segment (residues 281–288) following the α7 helix (Fig. 1a) where adjacent residues display elevated B-factors (> 65 Å^2^ vs. 34.2 Å^2^ average). Moreover, apo-FatB possesses a prominent loop (residues 244–254), protruding outward from the interdomain axis. These flexible elements distinguish apo-FatB from its homologs and may facilitate conformational adaptability upon ligand interaction.

This divergent interdomain geometry is supported by specific stabilizing motifs at the interface between the central α-helix and its flanking helices, α4 and α11 (Fig. 1a)^26^. The interaction between α4 and the central helix is mediated by a conserved electrostatic pair in both apo-FatB (K169–D193) and apo-YclQ, whereas such an interaction is not observed in apo-Pth (Supplementary Fig. 4). The contacts stabilizing the central helix against α11 show greater diversity. Unlike the homologs, which employ direct polar interactions to anchor the domains, either alone (apo-Pth) or reinforcing a water tether (apo-YclQ), FatB lacks such direct contacts and relies on a water-mediated tether (S195–W72–F329) to stabilize the central helix (Supplementary Fig. 4). Consequently, while apo-FatB shares the overall class-III SBP fold, these specific helical variations define a hinge architecture distinct from that of its structural homologs.

### Ferric ligand binding induces domain closure into a conserved closed conformation

**Fig. 2:**
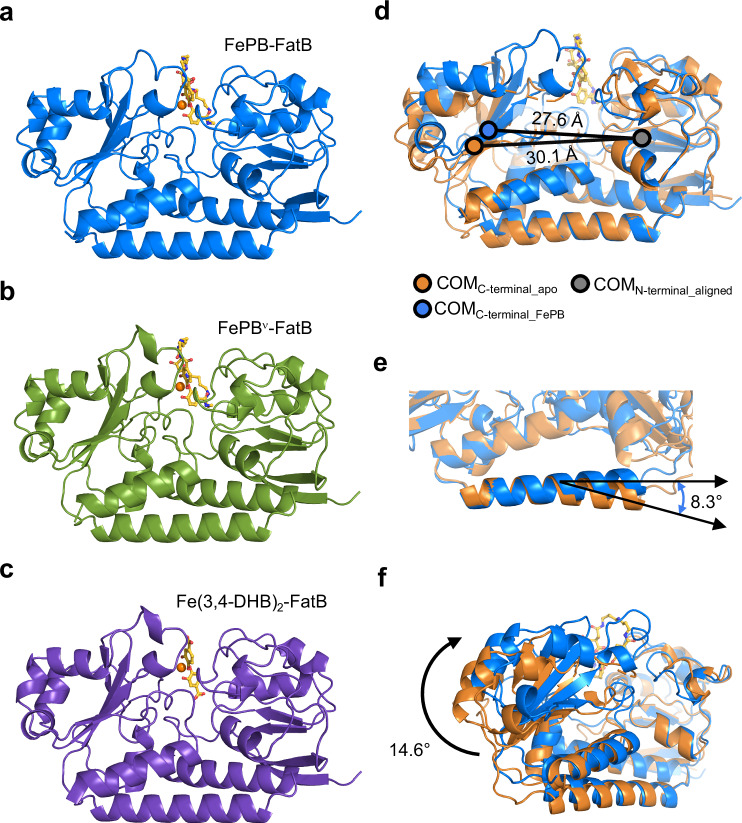
Crystal structures of FatB in complex with ferric ligands and analysis of the hinge-driven domain closure. Crystal structures of **a** FePB-FatB (blue), **b** FePB^ν^-FatB (green), and **c** Fe(3,4-DHB)_2_-FatB (purple). Ligands are shown as yellow sticks and Fe(III) ions as orange spheres. **d**–**f** Analysis of conformational changes based on the superposition of apo-FatB (orange) and FePB-FatB (blue), aligned on the N-terminal domain. **d** Hinge-driven domain closure illustrated by the reduction in distance between the COMs of the two domains. **e** Straightening of the central α-helix (residues 182–202) visualized by the angle change of the bent segment (residues 196–202). The 8.3° value shown corresponds to the direct apo-FatB/FePB-FatB superposition used for this representative panel. **f** Rotation of the C-terminal domain upon ligand binding, indicated by the curved arrow.

Photochemical modification of ferric siderophores can pose a challenge for SBP-mediated uptake because changes in the ligand scaffold may perturb the ferric coordination environment and the interaction features required for stable receptor capture and delivery^10^. This challenge motivated us to investigate FatB, for which previous biochemical work indicated that high-affinity binding was maintained even after photodecarboxylation of the ferric ligand. Specifically, apo-FatB binds its native ferric siderophore, FePB, the photolyzed derivative FePB^ν^, and a minimal catecholate mimic Fe(3,4-DHB)_2_ (Fig. 1d)^36^. However, the structural basis by which FatB accommodates these chemically distinct ligands has remained unclear (Fig. 1e). To elucidate this siderophore binding mechanism, we determined the crystal structures of FatB in complex with each ferric ligand (Supplementary Table 2). The resulting complexes, FePB-FatB, FePB^ν^-FatB, and Fe(3,4-DHB)_2_-FatB represent FatB bound to the native, photolyzed, and catecholate-core ligands in their ferric forms, respectively, providing a structural framework for analyzing ligand interactions across these distinct chemical states (Fig. 2a–c).

Compared with the apo-FatB structure, these ferric-ligand-bound FatB structures reveal a conserved transition from an open to a closed conformation, a characteristic hinge-like domain rearrangement observed in class-III SBPs (Supplementary Fig. 5). This transition produces clear crystallographic compaction, quantified by superposing the N-terminal domain of apo-FatB onto each ferric-ligand-bound FatB complex. The resulting conformational changes with respect to apo-FatB include a rotation of the C-terminal domain by 14.5 ± 0.5°, a straightening of the central α-helix by 8.3 ± 0.1° and a shortening of the interdomain distance by 3.0 ± 0.1 Å (Fig. 2d–f and Supplementary Table 3). Here, the reported values (mean ± standard deviation (SD)) were calculated across all three ferric-ligand-bound FatB complexes. These values place FatB within the range reported for class-III SBPs, between 20.5° in *Bacillus subtilis* FeuA and 8° in *Vibrio cholerae* FhuD, highlighting a moderate but functionally decisive motion^21,24,26^. The coupling of helix straightening with domain rotation effectively seals the interdomain cleft, encapsulating the siderophore within a protected binding pocket.

Despite the substantial transition from the open to the closed conformations, the three FatB complexes bound to ferric ligands adopt highly similar global structures. The N-terminal domains of any two complexes differ by only 0.17 ± 0.02 Å (RMSD), approximately one-quarter of the 0.61 ± 0.01 Å difference between the apo and any of the ferric-ligand-bound forms (Supplementary Table 4). This similarity extends to the entire protein, with an average overall Cα RMSD of just 0.20 ± 0.02 Å among the FatB complexes with ferric ligands, compared to 1.76 ± 0.08 Å for apo-FatB relative to the ferric-ligand-bound FatB structures (Supplementary Table 4).These findings demonstrate that ferric ligand binding stabilizes FatB into a highly consistent closed conformation, regardless of the specific structural variations among the bound siderophores, suggesting the formation of a consistent binding pocket.

### SAXS and CD corroborate ferric ligand-induced compaction of FatBin solution

**Fig. 3:**
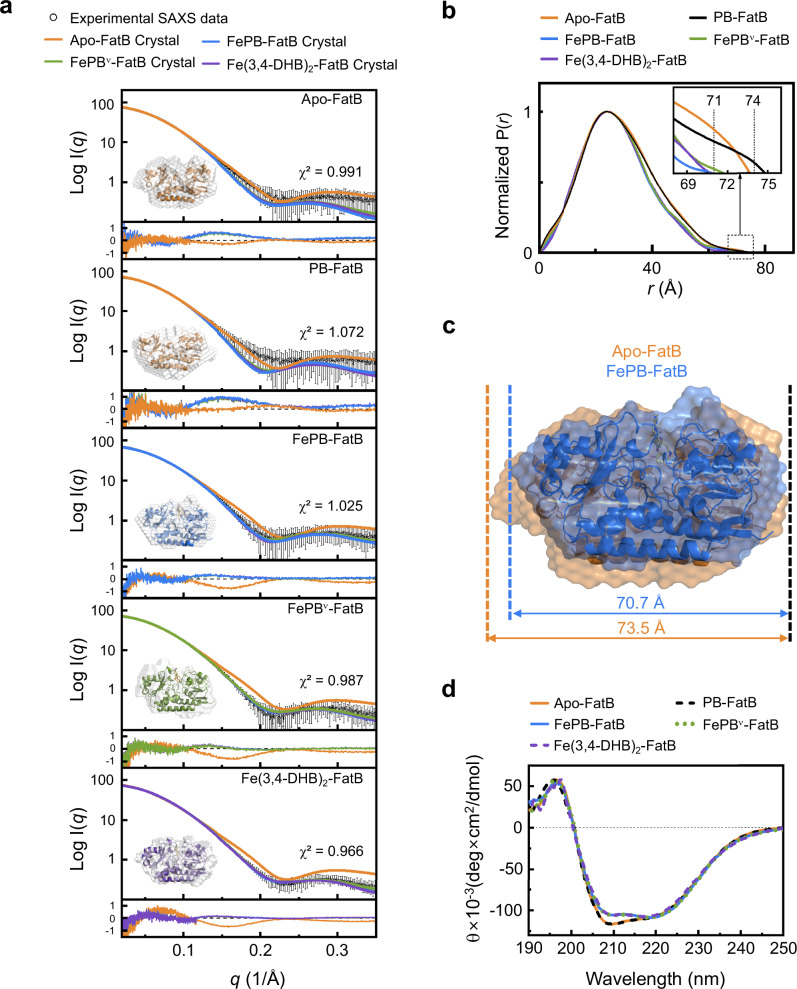
SAXS and CD analyses of apo- and ligand-bound FatB. **a** Experimental SAXS profiles (black points with error bars) for apo-FatB, PB-FatB, FePB-FatB, FePB^ν^-FatB, and Fe(3,4-DHB)_2_-FatB (top to bottom). Black points represent the mean scattering intensity obtained from *n* = 50 successive SAXS measurements for each sample, and error bars indicate ± SD across these measurements. The unit of study is an individual SAXS measurement collected from the same protein sample under continuous-flow conditions; thus, these represent technical replicates. Each experimental SAXS profile is overlaid with four CRYSOL curves calculated from the crystal structures of apo-FatB (orange), FePB-FatB (blue), FePB^ν^-FatB(green), and Fe(3,4-DHB)_2_-FatB (purple); the *χ*^2^ value in each panel refers to the crystal structure whose CRYSOL curve best matches the experimental data. For every state, the corresponding DAMMIN-refined ab initio bead model (gray) is shown with this best-fitting crystal structure superposed, and the residuals from the CRYSOL fits to the experimental SAXS data are plotted beneath the scattering profiles. **b** Pair-distance distribution functions *P(r)* derived from the SAXS data, showing a shift of *D*_*max*_ toward shorter *r* values for ferric-ligand-bound states relative to apo-FatB. **c** Superposition of the apo-FatB and FePB-FatB DAMMIN-refined bead models with their crystal structures, illustrating shortening of the long molecular axis upon ferric ligand binding, consistent with closure of the interdomain cleft. **d** Far-UV CD spectra of apo- and ligand-bound FatB. Apo- and PB-FatB display a slightly deeper minimum near 208 nm, whereas the 222 nm band overlays closely across all states, indicating that the overall secondary-structure content is maintained upon ligand binding. Colors are used consistently in **b**, **d**: apo-FatB (orange), PB-FatB (black), FePB-FatB (blue), FePB^ν^-FatB (green), and Fe(3,4-DHB)_2_-FatB (purple).

To determine whether the crystallographically observed domain closure occurs in solution, SAXS measurements were performed across the five FatB states: apo-, PB-, FePB-, FePB^ν^-, and Fe(3,4-DHB)_2_-FatB (Supplementary Table 5). Consistency between the solution and crystal structures was evaluated by fitting each experimental SAXS profile to theoretical scattering curves calculated from all available crystal structures using CRYSOL (Fig. 3a and Supplementary Table 6)^44^. The resulting *χ*² values clearly distinguished two conformational classes: the SAXS profiles of apo- and PB-FatB were best fit by the open apo structure, and those of the ferric-ligand-bound FatB complexes were best reproduced by their respective closed-state crystal models. This separation is also evident directly in the experimental data: difference SAXS curves relative to apo-FatB (Δ*I*(*q*) = *I*(*q*)_*ligand-bound FatB*_ − *I*(*q*)_*apo-FatB*_) remain close to zero for PB-FatB within experimental uncertainty, whereas those of all ferric-ligand-bound FatB complexes show clear, systematic deviations (Supplementary Fig. 6). The observation that PB-FatB retains the open conformation is consistent with the canonical mechanism of siderophore-binding proteins, where metal-free siderophore-protein complexes remain in an open conformation that closes upon metal coordination^17,45^. Applying the open-state model to the SAXS profiles of FatB complexes with ferric ligands, or fitting closed-state models to the apo-FatB profile, yielded substantially larger *χ*^2^ values, confirming that the distinct crystallographic conformations and the associated conformational compaction are maintained in solution.

The structural compaction accompanying domain closure was quantitatively confirmed by the SAXS-derived structural parameters (Table 1). To determine the radius of gyration (*R*_*g*_), we performed Guinier analysis for all FatB states, and the corresponding Guinier plots are provided in Supplementary Fig. 7. The resulting Guinier plots show linear behavior in the fitted low-*q* region (*qR*_*g*_ < 1.3) without a low-*q* upturn, and the symmetric, monodisperse peaks observed in the size-exclusion chromatography profiles (Supplementary Fig. 8) are consistent with sample monodispersity under the SAXS conditions. Based on the Guinier analysis, the open states (apo- and PB-FatB) share an *R*_*g*_ of approximately 22.1 Å, whereas the ferric-ligand-bound FatB complexes are more compact, with smaller *R*_*g*_ values ranging from 21.0 to 21.4 Å^46^. This shift is mirrored in the pair-distance distribution functions, *P(r)*, which exhibit bell-shaped profiles displaced toward shorter interatomic distances for the FatB complexes with ferric ligands (Fig. 3b)^46,47^. Consistently, the maximum particle dimension (*D*_*max*_) decreases from ~74 Å in the open states to roughly 71 Å in the closed states (Fig. 3b and Table 1). Ab initio envelope reconstructions visualize this transition as a reduction in the lateral width of the bilobal architecture while maintaining comparable thickness, consistent with cleft closure (Fig. 3c and Supplementary Fig. 9)^48–50^.

**Table 1:** Radius of gyration (*R*_*g*_) and maximum particle dimension (*D*_*max*_) from SAXS profiles obtained using the GNOM algorithm^47^

Complex	*R*_*g*_ (Å)	*D*_*max*_ (Å)	[*θ*]_222_/[*θ*]_208_
Apo-FatB	22.1	73.5	0.90
PB-FatB	22.1	74.8	0.90
FePB-FatB	21.2	70.7	1.01
FePB^ν^-FatB	21.4	71.3	1.01
Fe(3,4-DHB)_2_-FatB	21.0	71.2	0.98

The ratio of mean residue ellipticity at 222 and 208 nm ([*θ*]_222_/[*θ*]_208_) was used to assess α-helical character from CD spectra.

Finally, far-UV CD spectroscopy provided an independent probe of this compaction. The CD spectra for all states overlap closely near 222 nm but diverge around 208 nm, where open states exhibit a deeper 208-nm minimum than the ferric-ligand-bound FatB complexes (Fig. 3d). Consequently, the [*θ*]_222_/[*θ*]_208_ ratio, a diagnostic of α-helical packing and tertiary compaction, increases from almost 0.9 (apo-/PB-FatB) toward approximately 1.0 in the FatB complexes with ferric ligands (Table 1)^51,52^. This spectral signature indicates a transition toward a more tightly packed helical structure, aligning well with the straightening of the central α-helix observed in the crystal structures. Collectively, SAXS and CD demonstrate that ferric ligand binding drives a global, ligand-dependent compaction of FatB in solution, consistent with the conformational closure seen in the crystals.

### A conserved basic triad defines the catecholate-binding cleft

To connect the global conformational behavior of FatB with the atomic determinants of ligand recognition, we examined the binding cleft across all three ferric-ligand-bound FatB complexes. A hallmark of ligand recognition in class-III siderophore-binding proteins is the presence of a conserved basic triad. This motif generates the electropositive environment required for attracting anionic siderophores, and critically, establishes a charge-assisted hydrogen-bond network that neutralizes the deprotonated catecholate oxygens, thereby locking the ligand into the binding pocket^22–24,35,53^. In FatB, sequence and structural comparison identifies Arg129, Arg228, and Arg275 as the constituents of this canonical triad, forming the electropositive core of the cleft (Fig. 1c, Supplementary Figs. 2, 10, and 11). In the apo structure, this triad is largely pre-organized: Arg275 closely matches the conformation seen in YclQ and Pth, Arg129 maintains a conserved backbone position with modest side-chain variability, and Arg228 adopts a more permissive orientation at the edge of the pocket, suggesting built-in flexibility within an overall conserved framework.

**Fig. 4:**
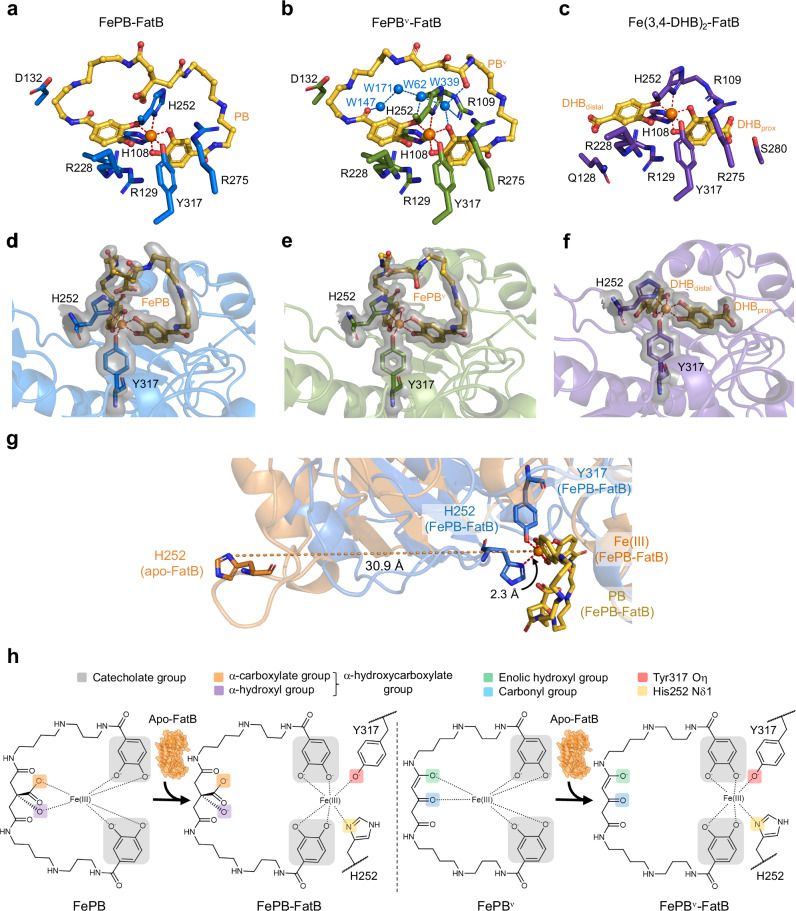
Binding-cleft architecture and Fe(III) coordination in FatB. Detailed binding-site geometries of **a** FePB-FatB, **b** FePB^ν^-FatB, and **c** Fe(3,4-DHB)_2_-FatB. Electron-density maps (2*mF*_*o*_*–DF*_*c*_, gray semitransparent surfaces contoured at 1.0 *σ*) overlaid on the refined structure models, highlighting the Fe(III) coordination by Tyr317 and His252 in **d** FePB-FatB, **e** FePB^ν^-FatB, and **f** Fe(3,4-DHB)_2_-FatB. FePB-, FePB^ν^-, and Fe(3,4-DHB)_2_-FatB are colored blue, green, and purple, respectively; interacting residues are shown as sticks in the corresponding protein colors, ligands as yellow sticks, Fe(III) ions are shown as orange spheres, and ordered water molecules (W147, W171, W62, and W339) in **e** are displayed as blue spheres. **g** Substantial displacement of His252. Superposition of apo-FatB (orange) and FePB-FatB (blue) reveals that His252 in apo-FatB is positioned 30.9 Å away from the Fe(III) position in FePB-FatB, indicating that a large-scale loop rearrangement is required to assemble the coordination sphere. **h** Two-dimensional schematic of FePB and FePB^ν^ in solution and in complex with FatB, highlighting how changes in the Fe(III) coordination sphere is reorganized around His252 and Tyr317. Catecholate groups (gray) are retained in all states, whereas the α-hydroxycarboxylate donors of FePB and the enolic hydroxyl and its adjacent carbonyl donors of FePB^ν^ are replaced, upon binding to FatB, by the protein-derived ligands Tyr317 Oη and His252 Nδ1 (color-coded as indicated).

Upon binding any of the three ferric ligands, this pre-formed scaffold becomes fully engaged, as the basic triad collaborates with the adjacent His108 to create a charge-assisted hydrogen-bond network that envelops the deprotonated catecholate oxygens and anchors the ligand within the cleft (Fig. 4a–c)^22,35^. The persistence of this stabilizing network across FePB-, FePB^ν^-, and Fe(3,4-DHB)_2_-FatB highlights its functional importance: even in the minimal Fe(3,4-DHB)_2_ complex, which lacks the aliphatic backbone of native siderophores, the ferric catecholate core occupies an essentially identical position, anchored precisely by the engagement of the basic triad and His108. Thus, the concerted action of these residues constitutes the fundamental binding motif in FatB for the 3,4-catecholate scaffold of the ligands, pre-organizing the cleft for catecholate recognition and providing the electrostatic framework on top of which the protein-derived Tyr/His coordination sphere is superimposed.

### A protein-derived Tyr/His pair forms the first coordination sphere of Fe(III)

Across all three ferric-ligand-bound FatB structures, a common structural feature is the direct coordination of the Fe(III) ion by the protein-derived Tyr317/His252 pair (Fig. 4d–f, Supplementary Figs. 12 and 13). The location of His252 in apo-FatB, 30.9 Å away from the Fe(III) center in the FePB-FatB, indicates that a substantial loop rearrangement is required to assemble this active site (Fig. 4g). This mode of protein participation is distinct from that of *Campylobacter jejuni* CeuE, which coordinates synthetic tetradentate analogs (n-LICAM) by completing the octahedral geometry^22,53^. Unlike these tetradentate analogs, free FePB and FePB^ν^ intrinsically adopt a hexadentate geometry, coordinating Fe(III) via four catecholate oxygens and their respective α-hydroxycarboxylate or enol/carbonyl tails (Fig. 1d)^10,35–38,54,55^.In the ferric-ligand-bound FatB complexes, the four catecholate oxygens remain ligand-derived, whereas a protein-derived Tyr/His pair (Tyr317 and His252) provides the remaining two Fe(III) ligands, completing the octahedral first coordination sphere (Fig. 4h).

The electron density map of the FePB-FatB complex provides clear evidence for this protein-derived coordination (Fig. 4d and Supplementary Fig. 12). The Fe-Tyr317 Oη and Fe-His252 Nδ1 contacts fall within canonical coordination distances, whereas the ligand’s α-hydroxyl oxygen (OAY) and α-carboxylate oxygen (OBY) are positioned at non-coordinating separations of 6.6 and 4.5 Å, respectively (Fig. 4a and Supplementary Fig. 14). When refinement was constrained to retain an α-hydroxycarboxylate coordination to Fe(III), OAY was drawn toward the metal center but refined to zero occupancy, and strong negative *mF*_*o*_*–DF*_*c*_ density appeared at the OBY, indicating that these atoms are misplaced in such a geometry (Supplementary Fig. 15). A Polder omit map confirmed the absence of any density for OBY (Polder CC −0.019, Supplementary Fig. 16a), while His252 and Tyr317 refined into well-defined density with strong Polder correlations (Polder CC > 0.85, Supplementary Fig. 16b, c). These observations demonstrate that the protein-derived Tyr/His pair replaces the ligand’s native donors in the FePB-FatB complex.

The same coordination strategy is observed in the FePB^ν^-FatB and Fe(3,4-DHB)_2_-FatB complexes. In FePB^ν^-FatB, the ligand’s enolic hydroxyl and carbonyl oxygens remain at non-coordinating distances (5.8 and 6.8 Å, respectively), blocked by a cluster of ordered water molecules (Fig. 4b, Supplementary Figs. 17 and 18). This model is fully consistent with the experimental density, where the lack of significant peaks in the *mF*_*o*_*–DF*_*c*_ map near the metal center confirms that no other ligand atoms participate in binding. Similarly, in Fe(3,4-DHB)_2_-FatB, although this ligand provides only a tetradentate catecholate core, the same Tyr317/His252 pair completes the Fe(III) coordination. In both cases, continuous electron density extends from the Fe(III) center to the coordinating protein residues, supporting the conserved mixed coordination mode (Fig. 4e, f, and Supplementary Fig. 12).

Together, these results establish a consistent coordination model for FatB: in all three ferric-ligand-bound FatB complexes, the protein actively remodels the Fe(III) sphere by consistently substituting the siderophore’s native donors with a Tyr/His pair.

### Ligand-specific adaptations at the binding interface

Although ferric ligand binding drives FatB into a conserved closed conformation featuring a shared Tyr317/His252-Fe(III) core, the binding pocket exhibits considerable plasticity, deploying distinct local adaptations for each ligand (Supplementary Fig. 19). In the FePB-FatB complex, the ligand’s global orientation is primarily secured by an external anchor: a hydrogen bond between the side chain of Asp132 and a secondary amine on the siderophore’s aliphatic backbone (Fig. 4a and Supplementary Fig. 19a). By anchoring the ligand in this manner, the intact α-hydroxycarboxylate tail is positioned to occupy the binding pocket adjacent to the metal center, leaving no internal cavity.

Upon photoreaction, free FePB releases CO_2_ from its α-hydroxycarboxylate, yielding the ferric photoproduct FePB^ν^, which can bind to FatB and leaves an internal cavity adjacent to the metal center (Fig. 4b). This decarboxylation removes the associated donor group, potentially destabilizing the ligand. The FePB^ν^-FatB structure reveals that FatB compensates for this loss by organizing four ordered water molecules (W339, W62, W171, and W147) into a structured relay that fills the physical void and replaces the missing interactions (Fig. 4b and Supplementary Fig. 18). Crystallographic data support the stability of the relay; the water molecules are well-defined with full occupancy and display relatively low B-factors (24.6, 21.1, 27.0, and 29.2 Å^2^), comparable to neighboring protein atoms, indicating a well-ordered interfacial solvent relay.

This templated solvent network is crucial for stabilizing the modified ligand (Fig. 4b and Supplementary Fig. 19b). By forming an extended hydrogen-bonding network, the relay restores interfacial connectivity between the siderophore and the protein. The network initiates at the solvent-facing side of the cavity with W339, which couples a catecholate oxygen to the backbone-side enolic hydroxyl oxygen of PB^ν^ and thereby stabilizes the backbone-side portion of the ligand within the remodeled pocket. In addition, W339 bridges these ligand oxygen atoms to the Arg109 guanidinium group, sustaining protein-ligand coupling at the interface. The relay is propagated by W62, which engages the second catecholate oxygen and transmits the solvent-mediated linkage into the interior of the network. This connectivity is further relayed through W171 to the terminal water W147, which hydrogen-bonds with the carboxylate oxygen on the catecholate-bearing arm of PB^ν^. Together, these waters form a continuous solvent-mediated hydrogen-bonding network that stabilizes the decarboxylated ligand across the remodeled FePB^ν^-binding interface (Fig. 4b). This solvent-templated relay functionally compensates for the lost interactions, while maintaining the octahedral geometry of the Fe(III) center.

To probe the binding plasticity with a minimal catecholate core, we analyzed the FatB complex with the tetradentate siderophore mimic, Fe(3,4-DHB)_2_ (Fig. 4c and Supplementary Fig. 19c). The two 3,4-DHB moieties coordinate Fe(III) through their catecholate oxygens; the proximal 3,4-DHB (DHB_prox_) maintains the same benzyl carbon orientation as in FePB or FePB^ν^, whereas the distal unit (DHB_distal_) adopts the opposite orientation, resulting in a reversed carboxylate group (Fig. 4c). Lacking the aliphatic backbone of FePB, Fe(3,4-DHB)_2_ is stabilized by alternative interactions: FatB engages the carboxylate of DHB_prox_ via hydrogen bonds with Arg109 or Ser280 and further adapts to the inverted orientation of DHB_distal_ by recruiting Gln128 to hydrogen-bond with its reversed carboxylate (Fig. 4c and Supplementary Fig. 19c). These local rearrangements demonstrate FatB’s ability to accommodate chemically simplified ligands by flexibly recruiting a different set of secondary stabilizing residues.

### His252 loop dynamics enable recruitment into the Fe(III) coordination sphere

Structural comparison with apo-Pth and apo-YclQ suggests that FatB possesses a distinctive hinge architecture consistent with enhanced interdomain flexibility. Previous study on related systems, *Vibrio cholerae* FhuD and HutB, demonstrated that interdomain mobility in class-III SBPs is governed by the packing of the central α-helix and its adjacent helices^26^. Resembling the more flexible *Vc*HutB, FatB features a central α-helix one turn shorter than in homologs and a less parallel arrangement of helices α4 and α11 (Supplementary Fig. 20a). This reduced packing likely facilitates interlobe motion and the substantial open-to-closed conformational transition upon ferric ligand binding. Notably, structural superposition of apo-FatB and the FePB-FatB complex highlights a spatial separation, where His252 in apo-FatB resides on a solvent-exposed loop, with its coordinating Nδ1 atom 30.9 Å away from the Fe(III) center in the FePB-FatB complex (Fig. 4g). Consistently, the position of the His252 imidazole-ring centroid differs by 28.6 Å between the two states, indicating that formation of the Tyr/His coordination sphere requires large-scale recruitment of the His252-containing loop into the binding cleft. This displacement underscores that high-affinity ferric capture would require substantial rearrangement of the His252-containing loop, raising the question of whether apo-FatB spontaneously samples loop-proximal conformations that could facilitate assembly of the Tyr/His site upon ligand encounter. To understand how His252 dynamically accesses the binding geometry, we performed molecular dynamics (MD) simulations of apo-FatB (Supplementary Data 1).

MD simulations indicate that apo-FatB is not a rigid scaffold and can transiently explore loop-proximal conformations that move His252 toward the ferric-ligand-bound site geometry. In particular, residues 244–254, which comprise the His252-containing loop, exhibits the highest root-mean-square fluctuation (RMSF) values, indicating substantial mobility (Supplementary Fig. 20b). Consistent with this flexibility, multiple frames capture the His252 loop extending toward the binding pocket, departing from the fully open state (Supplementary Fig. 20c). In a representative trajectory, the simulation captured an encounter-like event in which the COM of His252 imidazole ring approaches within 6.0 Å of the Fe(III) position in the FePB-FatB crystal structure (Supplementary Fig. 20d). In this transient configuration, the distance between the coordinating His252 Nδ1 atom and the putative Fe(III) position narrows to 7.7 Å. This dynamic sampling demonstrates that apo-FatB intermittently visits binding-proximal states where the His252 loop approximates the coordination geometry observed in ferric-ligand-bound FatB complexes.

### Ligand-dependent contributions of Tyr317 and His252 to FatB binding affinity

Our crystallographic analyses identified the Tyr317/His252 pair as the primary protein-derived contributors to the Fe(III) coordination sphere. To validate their functional importance, we performed site-directed mutagenesis (H252A, Y317F, and H252A/Y317F) and fluorescence quenching assays to determine dissociation constants (*K*_*d*_) (Supplementary Figs. 21, 22, and Supplementary Table 7)^56^. Both the metal-free (PB and 3,4-DHB) and their ferric counterparts (FePB and Fe(3,4-DHB)_2_), as well as the photolyzed derivative FePB^ν^, were tested to distinguish general binding from Fe(III)-dependent coordination effects (Table 2 and Supplementary Fig. 21).

**Table 2:** Dissociation constants (*K*_*d*_) for FatB wild-type and mutants binding various ligands, determined by fluorescence quenching^56^

Ligand	WT	H252A	Y317F	H252A-Y317F
	*K*_*d*_ (nM)	*K*_*d*_ (nM)	*K*_*d*_ (nM)	*K*_*d*_ (nM)
PB	85 ± 10	129 ± 8	124 ± 8	178 ± 21
FePB	105 ± 5	160 ± 11	206 ± 18	808 ± 102
FePB^ν^	29 ± 2	47 ± 3	43 ± 5	100 ± 13
3,4-DHB	72 ± 8	99 ± 8	156 ± 12	351 ± 39
Fe(3,4-DHB)_2_	24 ± 3	58 ± 5	260 ± 18	289 ± 57

Ferric complexes exhibited a stronger dependence on the Tyr/His pair than either the metal-free or photolyzed forms (Table 2). For the tetradentate Fe(3,4-DHB)_2_, the Y317F mutation alone caused an approximately 11-fold affinity loss from the high-affinity WT baseline (*K*_*d*_ ≈ 24 nM), comparable to the 12-fold decrease observed in the double mutant. This highlights Tyr317 as the dominant contributor to stabilizing this ferric catecholate complex. In contrast, FePB binding displayed a cooperative dependency relative to the WT baseline (*K*_*d*_ ≈ 105 nM). While single mutations increased the *K*_*d*_ by only 1.5–2-fold, simultaneous removal produced an almost 8-fold loss in affinity, confirming that Tyr317 and His252 function jointly to anchor the Fe(III) center.

The ferric photoproduct FePB^ν^ displayed a distinct profile among the ferric ligands. Despite high WT affinity (*K*_*d*_ ≈ 30 nM), binding was resilient to perturbations (Table 2). Both single mutants cause only modest, roughly 1.5–1.6-fold increases in *K*_*d*_, whereas the double mutant results in a 3.3-fold increase. The similar magnitude of affinity decreases for single mutants indicates that FePB^ν^ binding is not strongly biased toward either residue, and the 3.3-fold effect of the double mutant is consistent with an approximately additive contribution of two single mutations, rather than the cooperativity evident for FePB. Structural analysis reveals that FePB^ν^ accommodates a structured water network bridging the catecholates, Arg109, and the nitrogen atoms in the PB^ν^ backbone, providing an alternative interaction capability distinct from other FatB complexes. The relationship between this water-mediated interface and the muted mutational sensitivity of FePB^ν^ binding is addressed in the “Discussion”.

For the metal-free ligands, mutational effects were modest (Table 2). PB binding weakened only 2-fold in the double mutant (WT *K*_*d*_ ≈ 85 nM). The smaller catecholate fragment 3,4-DHB (WT *K*_*d*_ ≈ 72 nM) was slightly more sensitive: single mutants caused approximately 1.4- and 2.2-fold decreases in affinity, respectively, and the double mutant produced a fivefold reduction in affinity, likely due to fewer nonspecific hydrophobic contacts. Overall, these data establish that while Tyr317 and His252 contribute to the general pocket environment, they are structurally critical for ligands requiring direct protein-derived Fe(III) coordination.

### Spectroscopic and quantum-chemical analyses reveal Tyr317-mediated charge-transfer tuning

**Fig. 5:**
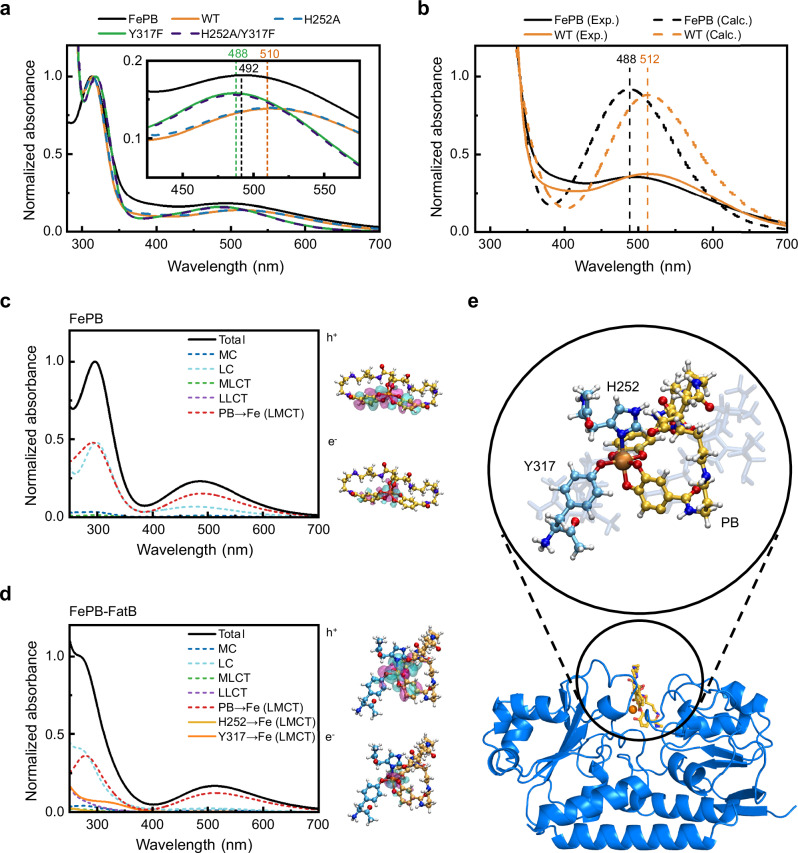
Comparative spectroscopic and computational characterization of the ferric coordination core in free FePB and FePB-FatB. **a** UV–vis spectra of free FePB and FePB-FatB complexes. Normalized absorption spectra are shown for free FePB (black) and FePB-FatB variants (WT, orange; H252A, blue; Y317F, green; H252A/Y317F, purple). The inset provides an expanded view of the low-energy absorption band (425–575 nm), highlighting the shift of the absorption maximum from 492 nm for free FePB to 510 nm in the WT and H252A complexes, and its reversal toward shorter wavelength (~488 nm) in the Y317F-containing mutants. **b** Comparison between the experimental (Exp.) and calculated (Calc.) absorption spectra for FePB and FePB-FatB. All spectra are normalized over the 340–700 nm range to facilitate direct comparison and to highlight the red shift in the lowest-energy band. The same normalization was applied to ensure that the observed red shift is evaluated consistently across the FePB and WT systems. The calculated spectra were generated by Gaussian convolution with a full width at half maximum (FWHM) of 0.30 eV. A 0.05 eV rigid red shift was applied to all calculated spectra. Decomposition of the calculated absorption spectra for **c** FePB and **d** FePB-FatB into the dominant electronic excitation characters obtained from the transition-density analysis. The contributions are grouped according to whether the excitation is primarily metal-centered (MC), ligand-centered (LC), or involves charge transfer between the metal and the ligands (LMCT/MLCT) or between ligands (LLCT)^57^. The individual ligand-to-metal charge-transfer (LMCT) contributions from the catecholate (PB), Tyr317, and His252 fragments are shown separately to highlight how the protein environment redistributes the donor contributions. For FePB, we show the natural transition orbital (NTO) pair with the largest weight associated with the lowest-energy LMCT transition, whereas for the FePB-FatB complex, we report the highest-weight NTOs of the higher-energy band; an identical isovalue of 0.015 was applied in both cases. **e** The QM/QM’ model employed in this work. The primary QM region (CAM-B3LYP; shown as opaque ball-and-stick representation) includes the Fe center and its coordinating ligands, while the surrounding protein environment treated at the QM’ level (SCAN-3c) is shown as semitransparent sky blue sticks.

Given that Tyr317 and His252 directly coordinate the Fe(III) center, we investigated how these residues perturb the excited-state manifold and the photochemistry of the FePB-FatB complex. As a baseline, we first analyzed free FePB in solution, which exhibits a broad absorption band centered near 492 nm and a more intense higher-energy feature at ~320 nm (Fig. 5a), both of which are well reproduced by our time-dependent density functional theory (TD–DFT) calculations^55^. To clarify the origin of these bands, we analyzed the charge-transfer character of the excited states^57–60^, a fragment-based excited-state analysis widely used to characterize charge-transfer states in complex molecular and condensed-phase systems^61–63^. This analysis shows that the 492 nm feature is dominated by catecholate → Fe(III) LMCT character, whereas the ~300–320 nm region, where the photodecarboxylation is typically induced, contains a heterogeneous mixture of LMCT and ligand-centered (LC) contributions (Fig. 5b, c)^38,64,65^.

Upon binding to WT FatB, the low-energy absorption band of FePB undergoes a red shift from ~492 to ~510 nm. This shift is consistent with a modification of the metal-coordination environment and is well reproduced by our TD–DFT calculations (Fig. 5b). Site-directed mutagenesis reveals the driver of this spectral tuning: the H252A mutant fully preserves this red-shifted signature, whereas removal of Tyr317 (Y317F or H252A/Y317F) abolishes the shift entirely, yielding a spectrum nearly indistinguishable from free FePB (Fig. 5a). These trends indicate that while His252 is structurally present, Tyr317 plays a pivotal role in shaping the excited-state character of the FePB-FatB complex.

To probe this behavior at the electronic-structure level, we modeled the excited-state spectrum of FePB-FatB using TD–DFT within a QM/QM’ framework, incorporating the local protein environment (Fig. 5d)^66–69^. The calculations again reproduce both the magnitude of the red shift and the overall spectral envelope (Fig. 5b), enabling a direct comparison between experiment and theory. Analysis of the excited-state character shows that the red shift does not arise from a change in the fundamental nature of the transition; in the FePB-FatB model, the lowest-energy LMCT state remains predominantly catecholate → Fe(III) in character, with negligible Tyr → Fe contribution^57–61^. Instead, Tyr317 reshapes the local coordination environment of the Fe(III) center, driving the complex toward a distorted octahedral arrangement, where the Fe-His252 bond becomes slightly elongated, while the Fe-Tyr317 bond is correspondingly shortened. Quantitatively, standard octahedral metrics reveal that FatB binding increases bond-length dispersion and anisotropy, whereas angular deviations and the overall degree of octahedral distortion remain largely unchanged (Supplementary Table 8)^70^. Thus, the FePB-FatB complex retains a near-ideal but directionally distorted octahedral geometry compared to free FePB. This structural reorganization modifies the relative energies of the metal center and its ligands, narrowing the donor–acceptor energy gap. Consequently, the LMCT transition shifts to lower energy, yielding the experimentally observed red shift, even though its fundamental nature remains unchanged.

At higher excitation energies (~320 nm), a distinct LMCT band of Tyr317 → Fe character emerges (Fig. 5d), whereas this contribution is negligible in the lowest-energy band (~510 nm). Concomitantly, the appearance of Tyr317 → Fe LMCT partially redistributes the overall charge-transfer manifold, competing with the dominant PB → Fe LMCT pathways and increasing the ligand-to-ligand charge-transfer (LLCT) character. Notably, this redistribution occurs within the ~300–320 nm window where the FePB photoreaction is generally induced. By structurally influencing both the low-energy absorption and the redistribution of high-energy excited states, Tyr317 exerts a substantial effect on the photochemical behavior of the FePB-FatB complex.

## Discussion

A notable feature of the FatB complexes with ferric ligands is the distinct coordination mode of His252, which binds Fe(III) via its Nδ1 atom rather than the typical Nε2 donor (Fig. 4e–g)^71^. Our structural refinement reveals a local hydrogen-bonding network where the protonated Nε2 atom is stabilized either by an ordered water molecule (W223 in FePB-FatB and W293 in Fe(3,4-DHB)_2_-FatB) or by the carbonyl oxygen of PB^ν^ in the FePB^ν^-FatB complex (Supplementary Fig. 23). This environment suggests that the protein may favor a tautomeric equilibrium where Nε2 remains protonated, making Nδ1 available for Fe(III) coordination (Supplementary Fig. 24). While unusual in siderophore-binding proteins, such Nδ1 coordination of histidine is a known strategy in other metalloproteins for fine-tuning electronic properties or redox potential^72,73^. In FatB, His252 adopts this mode alongside Tyr317, defining a mixed catecholate-tyrosinate/imidazole sphere with tailored electronic features.

Within this coordination framework, FatB exhibits remarkable plasticity, accommodating diverse ligands through specific adaptations. Our mutational data confirm that the Tyr317/His252 pair is structurally critical for ligands requiring direct protein-derived coordination, as evidenced by the significant affinity loss for FePB or Fe(3,4-DHB)_2_ upon mutation. In contrast, FePB^ν^ binding is partially affected against mutational perturbations: the modest effects of the single mutants and the approximately additive effect of the double mutant indicate a reduced reliance on any single residue and a redistribution of stabilizing interactions across the interface. A structural basis for this robustness is suggested by the FePB^ν^-FatB complex, where a structured water relay fills the cavity created by photodecarboxylation. This relay introduces an alternative network of hydrogen-bonding contacts linking the catecholate groups, Arg109, and the PB^ν^ backbone. This structural framework also provides a plausible basis for the higher observed affinity of FePB^ν^ relative to FePB (Table 2). Because the Tyr317/His252-Fe(III) coordination framework is conserved, the affinity difference is more likely to arise from ligand-specific second-sphere interaction at the binding interface. This second-sphere interaction is manifested as a solvent relay that provides compensatory hydrogen-bonding contacts and enhances the protein-ligand interaction network at the interface.

While apo-FatB retains the conserved class-III SBP fold, the protein adapts the hinge network to accommodate its specific interdomain arrangement, characterized by a shorter central α-helix and a less parallel arrangement of flanking helices. MD simulations demonstrate that the His252-containing loop intermittently visits binding-proximal states in the absence of ligand, consistent with loop mobility enabling His252 to access geometries proximal to the Fe(III) site observed in the ferric-ligand-bound FatB complexes. This conformational sampling suggests that the protein is poised for siderophore capture once a ligand becomes available. In parallel, Tyr317 provides a stable donor platform within the cleft. The interplay between the pre-positioned Tyr317 at the pocket base and the mobile His252 on a flexible loop represents a key element of FatB’s recognition mechanism. Consequently, the dynamic behavior of His252 and its subsequent stabilization through coordination establish a protein-derived Fe(III) center that couples conformational flexibility with precise electronic tuning, indicating how FatB stabilizes ferric siderophores while maintaining high-affinity recognition across chemically distinct ligands.

The cross-system comparisons with *Cj*CeuE, *Bs*FeuA, *Vc*FhuD, and *Vc*HutB given in the Results section can be organized into the following three characteristics that make FatB distinctive among class-III SBPs. First, at the level of Fe(III) coordination, while *Cj*CeuE provides a clear reference for protein participation in completing octahedral geometry for tetradentate ligands^22,53^, in FatB, a Tyr/His pair provides the decisive first-sphere donors even for ferric petrobactin species that are intrinsically hexadentate in solution. Second, FatB undergoes ligand-dependent conformational gating via hinge-driven domain closure, with a C-terminal domain rotation of ~15° placing FatB between the larger global motions reported for *Bs*FeuA and the smaller motions reported for *Vc*FhuD^21^. This comparison emphasizes that FatB lies in a moderate regime of global domain closure while ferric capture is accompanied by an unusually pronounced local rearrangement of the His252-containing loop. Third, FatB exhibits pronounced loop mobility centered on the His252-containing loop, which undergoes long-range recruitment upon ferric binding and constitutes the most dynamic region in apo-FatB by MD simulation. A previous comparison of *Vc*FhuD and *Vc*HutB suggested that looser packing around the central α-helix is associated with greater interdomain mobility and enhanced loop plasticity^26^. Consistent with this framework, apo-FatB shows a similarly less tightly packed environment around the central helix, including a one-turn-shorter central α-helix and a less parallel arrangement of helices α4 and α11, providing a plausible structural basis for the unusually large recruitment of His252 into the coordination sphere. Taken together, these comparisons suggest that FatB combines a permissive hinge architecture with unusually large His252-loop recruitment, providing a structural context for protein-derived Tyr/His coordination.

Beyond structural investigation, our spectroscopic and quantum-chemical analyses indicate that the protein environment, via Tyr317/His252 coordination, reshapes the excited-state manifold of FePB. Although the FePB^ν^-FatB structure was determined using FePB^ν^ generated prior to complex formation, FatB’s high affinity for FePB and FePB^ν^ suggests that the protein likely accommodates in situ photodecarboxylation. In such a case, our TD–DFT calculations reveal that the protein environment diminishes the α-hydroxycarboxylate → Fe(III) LMCT channel required for photodecarboxylation of FePB. This attenuation stems from the emergence of Tyr317 → Fe character and increased LLCT pathways in the high-energy region (~320 nm), competing with the dominant PB → Fe LMCT pathways. By redistributing the excited-state population, the protein environment effectively dilutes the contribution of the α-hydroxycarboxylate → Fe(III) channel. Consequently, this specific electronic tuning implies that FatB may modulate the likelihood of this photoreaction. Our findings lay the groundwork for future time-resolved investigations aimed at disentangling these specific photodecarboxylation kinetics within the binding pocket from bulk-solution pathways. Such studies would provide further insights into the reaction dynamics and mechanism by which the protein environment modulates this photochemical process.

Our structural characterization of FatB, integrated with spectroscopic and computational data, establishes the molecular basis for how this Gram-positive siderophore-binding protein recognizes and stabilizes ferric ligands. Our crystallographic and solution-phase analyses reveal that FatB undergoes a conserved hinge-like domain closure upon binding ferric ligands, employing a distinctive protein-derived coordination sphere where the Tyr317/His252 pair actively replaces the native donors of the ligand to complete the Fe(III) octahedral geometry. Collectively, these findings demonstrate that FatB is not merely a passive carrier but an active modulator of ferric coordination chemistry. By coupling global conformational flexibility with the precise recruitment of the protein-derived coordination sphere, FatB preserves robust iron acquisition across chemically distinct and labile ligands. This structural framework offers a blueprint for the development of siderophore-based Trojan Horse antibiotics. The plasticity of the FatB binding pocket suggests that siderophore-drug conjugates could be designed to exploit these versatile recognition motifs, facilitating the delivery of antimicrobial payloads into Gram-positive pathogens. Such strategies could provide a promising avenue to overcome the limit of conventional antibiotics in targeting bacterial iron uptake.

## Methods

### Cloning, expression, and purification of *Bacillus cereus* FatB

*Bacillus cereus* ATCC 14579 was obtained from the American Type Culture Collection. The gene encoding wild-type (WT) *Bacillus cereus* FatB without its signaling peptide (residues 40–338, hereafter referred to as FatB; Supplementary Table 1) was amplified by PCR from the genomic DNA of *B. cereus* ATCC 14579 and cloned into a 2B-T vector (a gift from Scott Gradia; Addgene plasmid # 29666) using the LIC (ligation-independent cloning) method. The resulting plasmid was designated as p2B-T-BcFatB-WT, which encodes an N-terminal His_6_-tag followed by a TEV protease cleavage site (ENLYFQ/S) upstream of the protein coding sequence. The mutants of FatB (H252A, Y317F, and H252A-Y317F) were generated by site-directed mutagenesis kit (EZ change site-directed Mutagenesis kit, Enzynomics) with the p2B-T-BcFatB-WT plasmid as a template. The primer sequences used for constructing FatB WT and mutants are provided in Table S1. The insertion of the *fatB* gene into the p2B-T-BcFatB-WT plasmid and the presence of the intended mutations in all constructs were confirmed by DNA sequencing.

Following sequence verification, each construct was transformed into *Escherichia coli* BL21 (DE3) for recombinant expression of FatB and its mutants. The bacteria expressing FatB WT or one of the mutants (H252A, Y317F, or H252A/Y317F) were grown in Luria-Bertani (LB) broth at 37 °C until the culture reached an OD_600_ of 0.6–0.8. Cells were induced with 0.4-mM IPTG and incubated for an additional 18 h at 25 °C. The cells were harvested by centrifugation (9334 × *g*, 5 min, 4 °C) and stored at −80 °C. The harvested cells were lysed by sonication in Lysis buffer (20-mM Tris-HCl, pH 8.0, 500-mM NaCl) and centrifuged (9334 × *g*, 30 min, 4 °C) to obtain the soluble fraction of the cell lysate. After centrifugation, the supernatant containing the recombinant protein was applied to IMAC Sepharose 6 Fast Flow (Cytiva), pre-equilibrated with Lysis buffer. The column was washed with Wash buffer (Lysis buffer containing 30-mM imidazole), and the protein was eluted with Elution buffer (Lysis buffer containing 250-mM imidazole). The His_6_-tag of the recombinant protein in the collected elution fractions was cleaved by TEV protease during overnight dialysis against 20-mM Tris-HCl, pH 8.0, at 4 °C. The dialyzed protein lacking the His_6_-tag was further purified using Q sepharose (Cytiva), equilibrated with Buffer A (20-mM Tris-HCl, pH 8.0). The dialyzed sample was loaded onto the column, and bound protein was eluted using a linear gradient of 0–50% Buffer B (Buffer A with 1-M NaCl). The eluted fractions were dialyzed against a TBS buffer (20-mM Tris-HCl, pH 8.0, 50-mM NaCl). After dialysis, the protein concentration was determined by measuring the absorbance at 280 nm using a UV–vis spectrophotometer (Cary 5000, Agilent), based on a calculated extinction coefficient of 32,430 M^−1^ cm^−1^. The full UV–vis spectrum (wavelength range of 250–700 nm) was also recorded to confirm the protein was in its ligand-free (apo) state. The protein was concentrated to 60 mg/mL using an Amicon Ultra centrifugal filter device (Millipore). The concentrated protein was aliquoted, flash-frozen in liquid nitrogen and stored at −80 °C.

### Preparation of Fe(3,4-DHB)_2_-FatB using the iron-limited culture medium of *B. cereus* ATCC 14579

*B. cereus* ATCC 14579 was cultured on LB agar plate at 37 °C overnight, and a single colony was inoculated into 10 mL of iron-limited medium prepared as described previously^74^. The small-scale culture was diluted 100-fold into a 1 L shaking flask containing the iron-limited medium and shaken at 37 °C for 20 h. The culture was centrifuged (9334 × *g*, 60 min) at 4 °C to remove the cell pellet, and the supernatant was filter-sterilized by passing through a VacuCap 90 Filter Unit with a 0.2 μm Supor Membrane (PALL). The filtered supernatant was mixed with 1 mL of purified apo-FatB (60 mg/mL) and stirred for 1 h at 4 °C. Fe(3,4-DHB)_2_-FatB was purified from the protein-supernatant mixture by Q Sepharose to remove the remaining cellular contents from the supernatant. The collected fractions were dialyzed against the TBS buffer overnight. After dialysis, the concentration of Fe(3,4-DHB)_2_-FatB was determined, and the protein was concentrated to 30 mg/mL using the same procedure as for apo-FatB.

### Preparation of Fe-petrobactin (FePB) and its photoproduct(FePB^ν^)

Petrobactin (PB) was synthesized by Accela Chembio. All experimental procedures using petrobactin were conducted under dim light. The obtained iron-free PB was dissolved in DMSO to have a molar concentration of 10 mM, flash-frozen in liquid nitrogen, and stored at −80 °C. To form FePB, 10-mM iron-free PB was titrated with 10-mM FeCl_3_, which was also dissolved in DMSO, to a final 1:1 molar ratio, and then vortexed. Then, the FePB solution was diluted with a buffer containing 20-mM Tris-HCl, pH 8.0 to have a final concentration of 1 mM.

To prepare FePB^ν^, the titrated and vortexed FePB solution (5 mM) was diluted to 0.5 mM with deionized water. The diluted FePB solution was stirred while irradiated at 325 nm by using a mounted LED (M325L5, Thorlabs) for 12 h at 4 °C. After irradiation, the mixture was further diluted with the buffer containing 20-mM Tris-HCl, pH 8.0, to have a final concentration of 0.1 mM. Photoconversion to FePB^ν^ was confirmed by monitoring the changes in the UV–vis absorption spectra (Supplementary Fig. 25).

### Preparation of siderophore-FatB complexes (PB-FatB, FePB-FatB, and FePB^ν^-FatB)

To prepare the FePB-FatB complex, 2 mL of concentrated apo-FatB was diluted to 2.4 mg/mL with 20-mM Tris-HCl, pH 8.0, and mixed with 10 mL of 1-mM FePB prepared in the same buffer. To prepare the FatB complex bound to iron-free petrobactin (PB-FatB), 2 mL of concentrated apo-FatB was similarly diluted and mixed with 10 mL of 1-mM PB prepared in the same buffer. To prepare the FePB^ν^-FatB complex, 2 mL of 60-mg/mL apo-FatB was added into 100 mL of 0.1-mM FePB^ν^ in 20-mM Tris-HCl, pH 8.0. In all three cases, the resulting complex was purified using Q Sepharose to remove excess ligands. The collected fractions were dialyzed overnight against the TBS buffer. After dialysis, the concentration of each purified FatB complex was determined, and each sample was concentrated to 30 mg/mL using the same procedure employed for apo-FatB.

### Fluorescence spectroscopy

Fluorescence quenching of recombinant FatB variants upon siderophore binding was monitored using a Fluoromax+ spectrofluorometer (HORIBA). Measurements were conducted with an excitation wavelength of 280 nm, while emission spectra were recorded from 300 to 400 nm with a slit bandpass of 4 nm. Measurements were performed at a protein concentration of 100 nM in the TBS buffer. The ligand solutions were freshly prepared prior to each series of measurements. The iron-free ligands, dissolved in DMSO at a concentration of 10 mM, were mixed with an equal volume of 10-mM FeCl_3_ solution (also dissolved in DMSO), and the mixture was subsequently diluted with the TBS buffer to a final concentration of 0.1 mM. For measurements with iron-free ligands, the FeCl_3_ solution was omitted, and the ligands were diluted to a final concentration of 0.1 mM. The ligand solutions were equilibrated for 2 h at 4 °C and then diluted with the TBS buffer to final concentrations of 1, 5, 10, 20, 50, and 100 μM. The protein solutions were titrated with ligand solutions, and allowed to equilibrate for 15 min prior to each fluorescence measurement. After normalizing the fluorescence intensity for dilution by the ligand solution, the fluorescence data were analyzed by nonlinear regression using a one-site binding model implemented in DYNAFIT (version 4.11.111; BioKin Ltd.) to describe the fluorescence response as a function of ligand concentration. The dissociation constants (*K*_*d*_) are reported as the mean values obtained from three independent titrations.

### Small-angle X-ray solution scattering (SAXS)

The static SAXS data for each FatB variant were collected at the 4 C beamline of Pohang Light Source-II (PLS-II), Pohang Accelerator Laboratory (PAL, Korea). Data were acquired using a flow-cell setup with a quartz capillary (1.0 mm diameter), where the sample was flowed at a rate of 2 mm/s to minimize X-ray radiation damage. The protein sample (3 mg/mL) was centrifuged (16,582 × *g*, 10 min, 4 °C), filtered through a 0.22 μm Millex PTFE syringe filter (Millipore), and loaded into the flow-cell system for SAXS measurements. The SAXS experiments were conducted with sample-to-detector distances of 1 and 3 m using an X-ray energy of 16.9 keV. An EIGER2 X 4 M detector was used to record the scattering images. The scattering images of the TBS buffer were also collected prior to each protein measurement. The scattering curves were obtained by performing azimuthal integration of the scattering image using processing software provided by the beamline.

Further SAXS data analysis was performed using PRIMUS (version 3.0.5) with a *q* range of 0.045–0.6 Å^−1^^46^ The buffer scattering curve was subtracted from the protein scattering curve to isolate the scattering signal only from the protein. *R*_*g*_ and *D*_*max*_ of each protein sample were determined by evaluating the pair distribution function *P(r)* using a software called GNOM (version 5.0)^47^. To visualize the three-dimensional solution shape, ab initio bead modeling was performed using DAMMIF (version r13837)^48^. Ten independent ab initio reconstructions were generated assuming P1 symmetry, and each reconstruction was further refined against the experimental scattering profile using DAMMIN (Refine with DAMMIN option; version r13837)^48^. The resulting bead models were superposed using DAMSUP (version r13837) to calculate the pairwise normalized spatial discrepancy (NSD) matrix, and a representative reconstruction was selected using DAMSEL (version r13837) based on the smallest mean NSD within the ensemble^49^. The alignment of the atomic crystal structure with the reconstructed SAXS envelope was performed using SUPCOMB (version r13837)^50^.

### Circular dichroism (CD) spectroscopy

To perform the CD spectroscopic measurements, the purified apo-, and ligand-bound FatB complexes were further dialyzed against 20-mM Tris-HCl, pH 8.0 at 4 °C overnight and diluted to a final concentration of 0.5 mg/mL. CD spectroscopy was carried out using a CD spectrophotometer (J-1700, Jasco) at ambient temperature under a continuous flow of nitrogen. For the CD measurements, the following parameters were used: wavelength range: 180–250 nm, data pitch: 0.2 nm, scanning speed with continuous mode: 50 nm/min, bandwidth: 1 nm, three times of accumulation, and pathlength: 0.5 mm. The CD spectra of protein samples were measured, and the corresponding buffer baseline was recorded under the same parameters. Each protein CD spectrum was corrected by subtracting the corresponding buffer baseline spectrum.

### Crystallization of FatB in its apo and ferric ligand-bound forms

Apo-FatB and FatB complexes with three different ferric ligands (Fe(3,4-DHB)_2_, FePB, FePB^ν^) were crystallized. Before crystallization, the stored protein samples were fully thawed and diluted with the TBS buffer. Initial crystallization screening of all FatB forms was performed at protein concentrations of 10, 15, 20, and 30 mg/mL using three commercial screening kits: Index, PEGRx, and PEG/Ion (Hampton Research). For apo-FatB, an initial crystallization condition was obtained from a condition containing 0.1-M potassium thiocyanate, 30% (w/v) PEG monomethyl ether 2000. Fe(3,4-DHB)_2_-FatB crystals were first obtained from a condition containing 20% (v/v) 2-propanol, 0.1-M MES monohydrate, pH 6.0, 20% (w/v) PEG monomethyl ether 2000. For both FePB-FatB and FePB^ν^-FatB, initial crystallization conditions were observed in drops containing 10% (v/v) 2-propanol, 0.1-M BICINE, pH 8.5, 30% PEG 1500. The crystallization conditions were further optimized by screening precipitant concentrations and pH values around the initial hits. The final crystallization conditions for each FatB form were as follows: apo-FatB (15 mg/mL) crystallized in 0.15-M potassium thiocyanate, 30% (w/v) PEG monomethyl ether 2000; Fe(3,4-DHB)_2_-FatB (10 mg/mL) crystallized in 15% (v/v) 2-propanol, 0.1-M MES monohydrate, pH 6.0, 30% (w/v) PEG monomethyl ether 2000; FePB-FatB (10 mg/mL) crystallized in 10% (v/v) 2-propanol, 0.1-M BICINE, pH 8.5, 36% (w/v) PEG 1000; and FePB^ν^-FatB (10 mg/mL) crystallized in 10% (v/v) 2-propanol, 0.1 M BICINE, pH 8.5, 28% (w/v) PEG 1000.

### Data collection, structure determination, and analysis

X-ray diffraction data from protein crystals under cryogenic conditions were collected at 100 K on the 11 C beamline of PLS-II at Pohang Accelerator Laboratory (PAL, Korea). The X-ray energy was 12.659 keV, and the sample-to-detector distance of 350 mm, resulting in a resolution of 1.78 Å at the edge of the diffraction image. Prior to data collection, single crystals of apo-FatB and ferric-ligand-bound FatB complexes were coated with LV CryoOil (Mitegen) and flash-frozen in liquid nitrogen. Diffraction images were recorded on a Pilatus 3 6 M detector (Dectris), with each crystal exposed for 1 s per 1.0° rotation of ω. All diffraction data were indexed, integrated, and scaled using HKL2000 (version 722) and HKL3000 (version 722) (HKL Research Inc.).

The structures of all FatB forms were solved by molecular replacement using Phaser MR (version 2.8.3) in the PHENIX software package (version 1.20.1-4487), with a crystal structure of FatB from Desulfitobacterium hafniense (PDB code: 7SF6) as a search model. For each FatB form, the initial MR solution was rebuilt and refined using AutoBuild^75^ and phenix.refine^76^ modules of PHENIX (version 1.20.1-4487)^77^, and further manual model adjustment and ligand fitting were performed in Coot (version 0.9.8.95)^78^. The electron densities corresponding to the ligands were fitted and refined in Coot using real-space refinement, after importing the ligand coordinates with their three-letter codes (F8W for petrobactin, DHB for 3,4-DHB, and FE for Fe(III) ion) and placing them in the corresponding positive electron densities. The final model of apo-FatB included residues 44–337, and the Fe(3,4-DHB)_2_-FatB model included residues 43–337, and the FePB-FatB and FePB^ν^-FatB models included residues 43–338. The coordinates and structure factors for all FatB forms have been deposited in the Protein Data Bank under accession codes: apo-FatB (PDB 21ZD), FePB-FatB (PDB 21ZE), and FePB^ν^-FatB (PDB 21ZF), Fe(3,4-DHB)_2_-FatB (PDB 21ZG).

For rigorous validation of the ligand binding modes, polder omission maps were calculated using the phenix.polder^79^ module in PHENIX (version 1.20.1-4487) to minimize bulk-solvent density interference. Additionally, to analyze specific hydrogen bonding networks stabilizing the metal coordination sphere, hydrogen atoms were added to the final refined models using the program Reduce (version 3.7)^80^. In this analysis, the His252 residue was explicitly modeled as the HIE tautomer (protonated at Nε2) to verify the donor-type hydrogen bonds. Note that these hydrogen atoms were generated solely for geometric analysis and were not included in the deposited coordinates.

### Molecular dynamics simulations

All-atom MD simulations of apo-FatB were performed using GROMACS 2022.2^81^ with the CHARMM36 force field^82^ and the SPC/E water model(Supplementary Table 9)^83^. The system was solvated in a periodic box and neutralized with Na^+^ counterions. Histidine protonation/tautomer states were kept as assigned in the initial topology (no manual reassignment was performed). Long-range electrostatics were treated using the particle-mesh Ewald (PME) method^84,85^, while van der Waals interactions and short-range electrostatics were cut off at 1.0 nm. The temperature (300 K) and pressure (1 bar) were maintained using the velocity-rescaling thermostat (time constant = 0.1 ps) and the Parrinello–Rahman barostat^86^ (time constant = 2.0 ps), respectively. Bond lengths involving hydrogen atoms were constrained using the LINCS algorithm^87^, allowing a 2 fs integration timestep. Three independent replicas were simulated for 250 ns each (total 750 ns). Replicas were initiated with independently assigned initial velocities drawn from a Maxwell–Boltzmann distribution during NVT equilibration (gen_vel = yes; gen_seed = −1, random seed), and subsequent NPT/production runs were continued without regenerating velocities (gen_vel = no). Convergence was evaluated by monitoring backbone RMSD and *R*_*g*_ over the full trajectory range (Supplementary Fig. 26). Based on these convergence metrics, the initial 50 ns of each trajectory were treated as equilibration and excluded from all downstream analyses; quantitative analyses were performed over the subsequent 200-ns production window (50–250 ns). During equilibration (NVT and NPT), harmonic positional restraints were applied to the protein heavy atoms (define = -DPOSRES), whereas production trajectories were run without positional restraints or enhanced sampling biasing potentials. Periodic boundary artifacts were removed prior to analysis, and trajectories were aligned to the N-terminal domain (residues 44–181) of the FePB-FatB crystal structure. Loop dynamics were quantified by measuring the distance from the fixed Fe(III) position (defined by the superimposed FePB-FatB structure) to (i) the center of mass (COM) of the His252 imidazole ring and (ii) the His252 Nδ1 atom. Root-mean-square fluctuation (RMSF) values were calculated for Cα atoms after aligning the N-terminal domains.

### System models

To investigate how the protein environment modulates the optical response of Fe(III) complexes, we examined two representative systems: the isolated FePB complex, in which all atoms contributing to the octahedral coordination originate from the PB ligand, and the FePB-FatB complex, which corresponds to the WT binding configuration, and is embedded within its native protein environment, as characterized in this work. Solvent effects for both systems were included using the conductor-like polarizable continuum model (CPCM) with water as the dielectric medium^88^.

For FePB-FatB, the computational model comprised the Fe(III)-centered coordination sphere together with neighboring amino acid residues that define the binding cleft and form hydrogen bonds or electrostatic contacts with the ligand and that fall within a radius of ~15 Å from the Fe(III) center. The specific model used for the calculations is shown in Fig. 4d. The Fe(III)-coordinating ligands were treated at the QM level, while the interacting protein residues were described using a lower-level QM’ treatment, providing a balanced and physically meaningful representation of environmental polarization and charge redistribution, as detailed below.

### DFT and TD–DFT calculations

All ground-state geometries were optimized at the CAM-B3LYP^89^ level of theory, including Grimme’s D3 dispersion correction^90^. A mixed basis-set approach was employed, using def2-TZVP for the Fe center and def2-SVP for all other atoms together with the def2/J auxiliary basis. All electronic-structure calculations were performed using the ORCA program package^91^.

For the FePB-FatB system, a multilayer QM/QM’ approach was used: the FePB binding site in FatB was treated at CAM-B3LYP, while the surrounding residues were described at the SCAN-3c level^92^. Geometry optimization was performed only for the QM region using a loose optimization setup in order to avoid artificial close contacts or unphysical rearrangements at the QM/QM’ boundary.

Vertical excitation energies and oscillator strengths were computed using TD–DFT. Simulated absorption spectra were generated by convoluting individual transitions with Gaussian functions of 0.30 eV full width at half maximum (FWHM). For FePB, the first 65 excited states were computed, whereas for FePB-FatB, the first 100 roots were evaluated.

A detailed description of the excited-state analysis, including the use of natural transition orbitals (NTOs) and the one-particle transition-density matrix (1TDM), is provided in Supplementary Note 1.

### Reporting summary

Further information on research design is available in the Nature Portfolio Reporting Summary linked to this article.

## Supplementary information

Supplementary Information

Description of Additional Supplementary Files

Supplementary Data 1

Reporting Summary

Transparent Peer Review File

## Source data

Source Data

## Data Availability

All crystallographic coordinates and associated structure-factor data have been deposited into the Protein Data Bank (PDB) under accession codes 21ZD (apo-FatB), 21ZE (FePB-FatB), 21ZF (FePB^ν^-FatB), and 21ZG (Fe(3,4-DHB)_2_-FatB). Previously published structures used in this study are available in the PDB under accession codes 8BNW (apo-Pth), 3GFV (apo-YclQ), and 7SF6 (*Desulfitobacterium hafniense* FatB). The SAXS data have been deposited in the SASBDB database under accession codes SASDYB7 (apo-FatB), SASDYC7 (PB-FatB), SASDYD7 (FePB-FatB), SASDYE7 (FePB^ν^-FatB), SASDYF7 (Fe(3,4-DHB)_2_-FatB). MD simulation parameter and coordinate files are provided as Supplementary Data 1. Source data are provided as a Source data file. Source data are provided with this paper.
