# Supplementary material for: Structural basis of FatB-mediated iron uptake via tyrosine/histidine direct coordination accompanying long-distance domain reorganization
Source: Nat Commun. 2026 Apr 18;17:5413. doi: 10.1038/s41467-026-72127-y (PMC13279818; doi:10.1038/s41467-026-72127-y)
Supplement: Supplementary file 1 — Supplementary Information [file 41467_2026_72127_MOESM1_ESM.pdf]

Supplementary Information

Structural basis of FatB-mediated iron uptake via tyrosine/histidine direct coordination accompanying long-distance domain reorganization

Hyosub Lee[1,2], Seong Ok Kim[1,2], Seyoung You[1,2], Alekos Segalina[1,2], Taeyoon Noh[1,2], and Hyotcherl Ihee*[,1,2]

[1]Department of Chemistry, Korea Advanced Institute of Science and Technology (KAIST); Daejeon 34141, Republic of Korea

[2]Center for Advanced Reaction Dynamics (CARD), Institute for Basic Science (IBS); Daejeon 34141, Republic of Korea

*Corresponding author. Email: hyotcherl.ihee@kaist.ac.kr (H.I.)

This PDF file includes:

Supplementary Figure 1–26

Supplementary Table 1–9

Supplementary Note 1

Supplementary References

Table of contents

Supplementary Figure 1. Relative solvent accessibility (rASA) of apo-FatB.

Supplementary Figure 2. Sequence alignment of FatB and its homologous siderophore-binding proteins.

Supplementary Figure 3. Structural comparison of apo-FatB with homologous siderophore-binding proteins.

Supplementary Figure 4. Comparison of inter-domain stabilizing interactions in apo-FatB and its homologs.

Supplementary Figure 5. Ligand-dependent conformational states of FatB.

Supplementary Figure 6. Difference SAXS profiles between apo-FatB and ligand-bound states.

Supplementary Figure 7. Guinier plots of apo- and ligand-bound FatB states.

Supplementary Figure 8. Size-exclusion chromatography profiles of FatB complexes used for SAXS measurements.

Supplementary Figure 9. Most-probable and DAMMIN-refined ab initio bead models for FatB states.

Supplementary Figure 10. Superposition of apo-FatB with apo-Pth and apo-YclQ showing conserved binding-cleft residues.

Supplementary Figure 11. Representative electron density map in the binding-cleft region of apo-FatB.

Supplementary Figure 12. Close-up electron density maps highlighting the Fe(III) coordination environment in ferric ligand-bound FatB.

Supplementary Figure 13. Ligand omit maps for ferric ligand-bound FatB complexes.

Supplementary Figure 14. Crystallographic validation of the Tyr/His coordination sphere in FePB-FatB.

Supplementary Figure 15. Electron density maps of the α-hydroxycarboxylate–retained alternative FePB-FatB model.

Supplementary Figure 16. Polder omit maps calculated from an alternative model where the ligand's α-hydroxycarboxylate group was enforced to coordinate the Fe(III) ion.

Supplementary Figure 17. Electron-density comparison of FePB and $FePB^v$ in the FatB binding site.

Supplementary Figure 18. Electron density maps of $FePB^v$-FatB around the Fe(III) center.

Supplementary Figure 19. Hydrogen-bonding networks in the FatB binding clefts.

Supplementary Figure 20. Structural and dynamic basis of hinge flexibility and loop mobility in apo-FatB.

Supplementary Figure 21. Fluorescence quenching analysis of FatB variants with ligands.

Supplementary Figure 22. SAXS analysis of FatB variants in apo and FePB-bound states.

Supplementary Figure 23. His252 δ-coordination stabilized by Nε2–H···O hydrogen bonds across all FatB complexes bound to ferric ligands.

Supplementary Figure 24. Modeling trials demonstrate that His252 coordinates Fe(III) via Nδ1, not Nε2.

Supplementary Figure 25. UV-visible absorption spectra of FePB and $FePB^v$.

Supplementary Figure 26. Convergence assessment of apo-FatB MD simulations.

Supplementary Table 1. Oligonucleotide primers used for cloning and site-directed mutagenesis of *Bc*FatB.

Supplementary Table 2. X-ray data collection and refinement statistics.

Supplementary Table 3. Quantification of ligand-induced conformational changes in FatB.

Supplementary Table 4. Pairwise Cα RMSD analysis of FatB complexes.

Supplementary Table 5. Details for SAXS measurements and analysis.

Supplementary Table 6. Goodness-of-fit (χ^2) values from CRYSOL analysis comparing experimental SAXS

profiles to theoretical crystal structure models.
Supplementary Table 7. Structural parameters derived from SAXS data for FatB variants.
Supplementary Table 8. Essential octahedral metrics for FePB and FePB-FatB.
Supplementary Table 9. System setup for apo-FatB MD simulations.
Supplementary Note 1. Excited-state analysis of the FePB-FatB complex.
Supplementary References

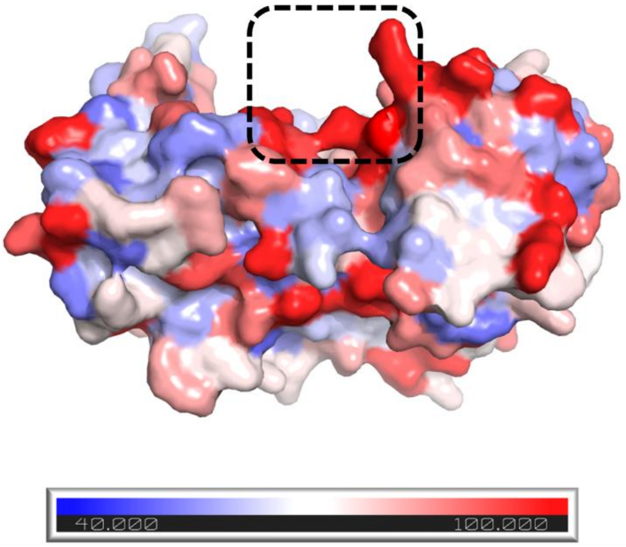

Supplementary Figure 1. Relative solvent accessibility (rASA) of apo-FatB. Per-residue rASA (0–100%) computed as the sum of atomic SASA with a 1.4 Å probe, normalized by the residue-specific maximal ASA is mapped onto the structure (blue → white → red). The dashed box highlights the binding cleft of apo-FatB, which is highly solvent-accessible relative to the surrounding surface.

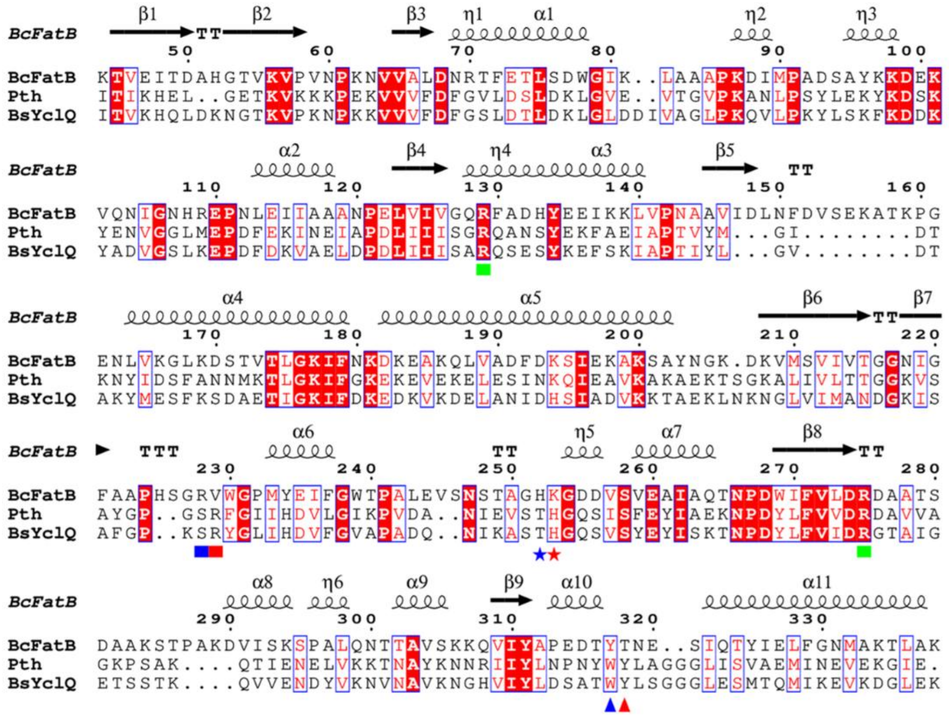

Supplementary Figure 2. Sequence alignment of FatB and its homologous siderophore-binding proteins. Amino acid sequences of *Bacillus cereus* FatB (BcFatB), *Parageobacillus thermoglucosidasius* CeuE homolog (Pth), and *Bacillus subtilis* YclQ (BsYclQ) were aligned using Clustal Omega, and the alignment was visualized with ESPript 3.0[1,2]. Secondary structure elements of apo-FatB, as assigned by the Define Secondary Structure of Proteins algorithm, are shown above the alignment (coils for α- and 3_{10}-helices, arrows for β-strands, and TT represents β-turns)[3]. Symbols below the sequences indicate functionally important residues that are conserved among the compared proteins: stars (★) mark His252 in BcFatB and its aligned histidines in Pth and BsYclQ (at position 253), triangles (▲) denote Tyr317 in BcFatB and the corresponding tyrosines in the homologs (at position 318), and squares (■) represent the basic arginine triad involved in anion coordination (Arg129, Arg228, Arg275 in BcFatB) and their aligned counterparts (with the Arg228 equivalent appearing at position 229). Because the secondary-structure annotation follows the BcFatB numbering, these symbols in Pth and BsYclQ appear shifted by one residue but still refer to the same conserved alignment columns. Color coding distinguishes the origin of each marked residue: blue symbols indicate the position in *B. cereus* FatB, red symbols denote the corresponding residues in homologs, and green symbols highlight positions where all three proteins share the same aligned residue. Gaps in the secondary structure annotation for BcFatB correspond to disordered regions (e.g., residues 281-288) that could not be modeled in the apo-FatB crystal structure.

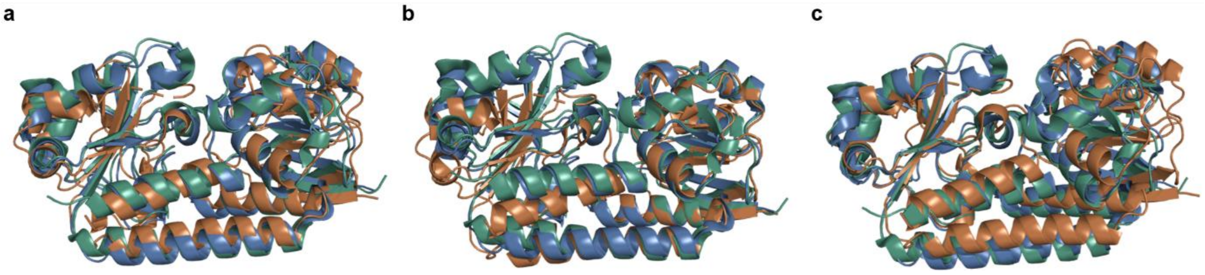

Supplementary Figure 3. Structural comparison of apo-FatB with homologous siderophore-binding proteins. Cartoon representations of apo-FatB (orange), apo-Pth (blue), and apo-YclQ (green) are shown[1,2]. Superpositions based on (**a**) the entire protein, (**b**) the N-terminal domains, and (**c**) the C-terminal domains demonstrate that all three proteins share a conserved bilobal fold with only minor differences in interdomain orientation, consistent with their low sequence identity but high structural similarity.

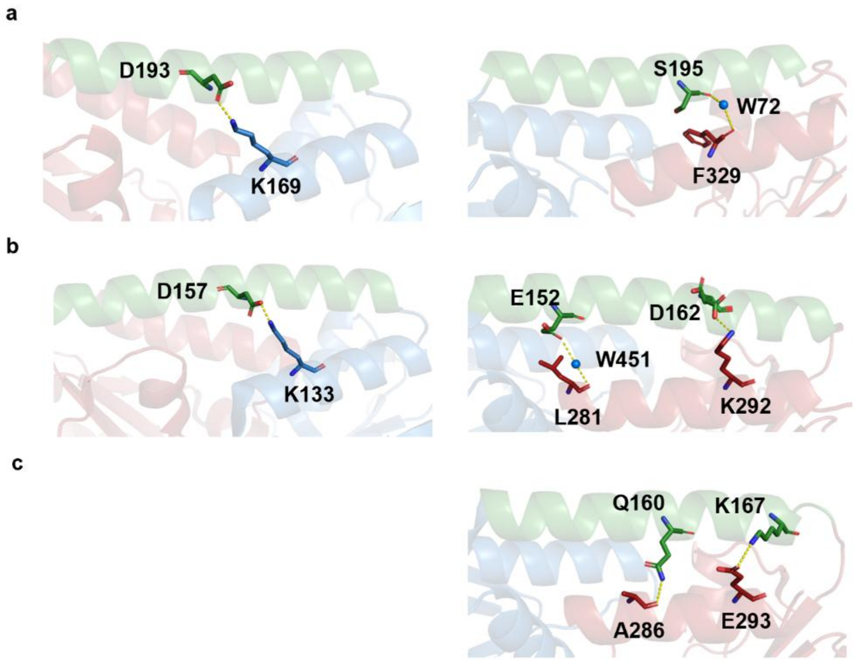

Supplementary Figure 4. Comparison of inter-domain stabilizing interactions in apo-FatB and its homologs. The left column shows interactions between the central α-helix (green) and the final α-helix of the N-terminal domain (blue), and the right column displays interactions between the central α-helix and the final α-helix of the C-terminal domain (red). (**a**) In apo-FatB, stability is provided by an N-terminal electrostatic pair (K169–D193) and a C-terminal water-mediated tether (S195–W72–F329). (**b**) In apo-YclQ, a similar N-terminal electrostatic pair (K133–D157) is present, while the C-terminal domain is stabilized by both a water-mediated tether (E152–W451–L281) and a direct electrostatic interaction (D162–K292)[2]. (**c**) In apo-Pth, no corresponding direct interaction between the N-terminal domain and the central α-helix was observed (left panel). C-terminal domain stability is maintained by two direct interactions (Q160–A286 and K167–E293)[1].

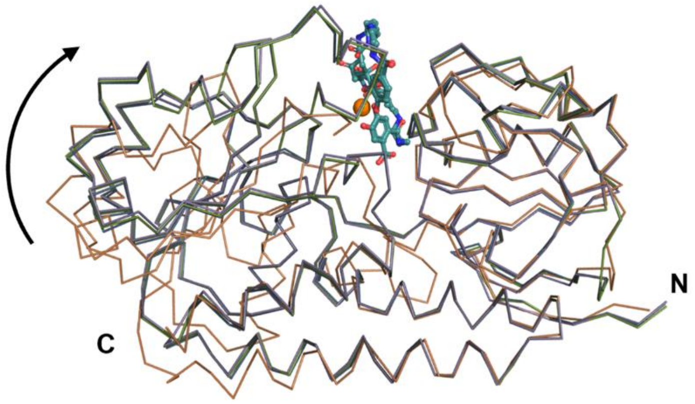

Supplementary Figure 5. Ligand-dependent conformational states of FatB. Superposition of apo-FatB (brown), FePB-FatB (green), FePB^{v}-FatB (blue), and Fe(3,4-DHB)$_2$-FatB (purple), aligned on the N-terminal domain. Each structure is shown as a ribbon representation with the ferric ligand (cyan sticks) and Fe(III) ion (orange sphere). Relative to apo-FatB, all ferric-ligand-bound FatB structures adopt a more closed orientation of the C-terminal domain toward the N-terminal domain. The arrow indicates the direction of this domain rearrangement. Labels N and C mark the N- and C-termini of FatB, respectively.

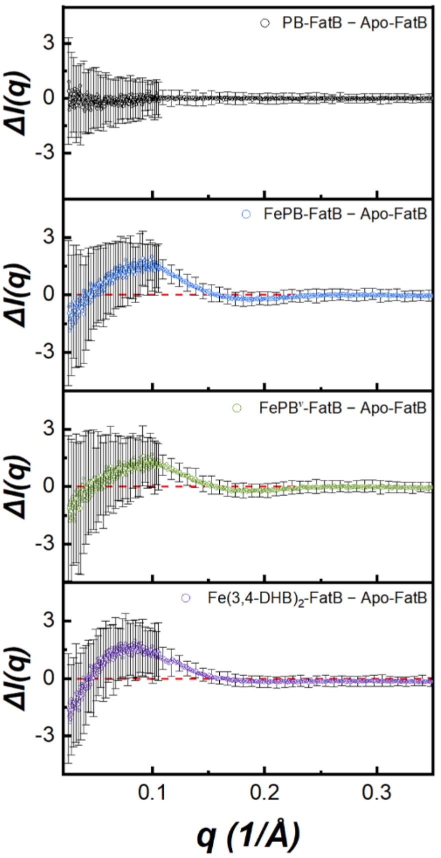

Supplementary Figure 6. Difference SAXS profiles between apo-FatB and ligand-bound states. Difference SAXS profiles were calculated as $\Delta I(q) = I(q)_{ligand\text{-}bound\ FatB} - I(q)_{apo\text{-}FatB}$ using buffer-subtracted SAXS profiles placed on the same intensity scale (identical concentration normalization for all states). Scattering curves are shown for PB-FatB, FePB-FatB, FePB^{v}-FatB, and Fe(3,4-DHB)$_2$-FatB relative to apo-FatB, with panels arranged from top to bottom in this order. Error bars represent propagated experimental uncertainties, and the red dashed line indicates $\Delta I(q) = 0$.

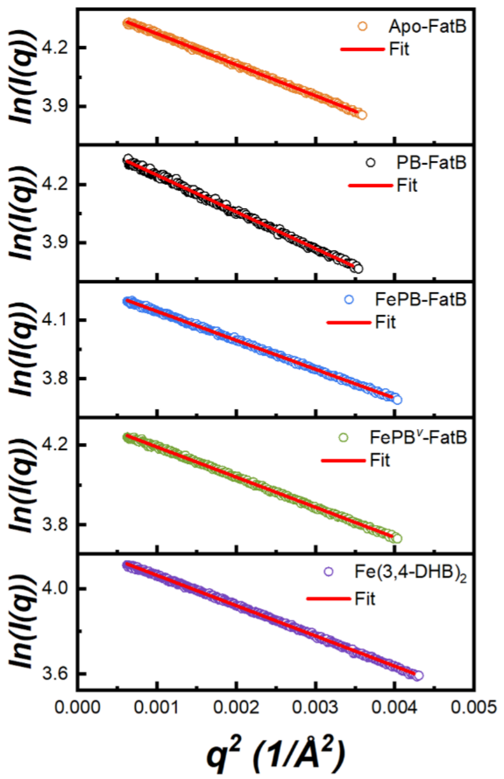

Supplementary Figure 7. Guinier plots of apo- and ligand-bound FatB states. Guinier plots (*ln I*(*q*) vs q^2) are shown for apo-FatB, PB-FatB, FePB-FatB, FePB^{v}-FatB, and Fe(3,4-DHB)$_2$-FatB. Linear Guinier fits (red lines) were performed within the low-q region satisfying the standard Guinier criterion (qR_g < 1.3), and the resulting R_g values are reported in Table 1.

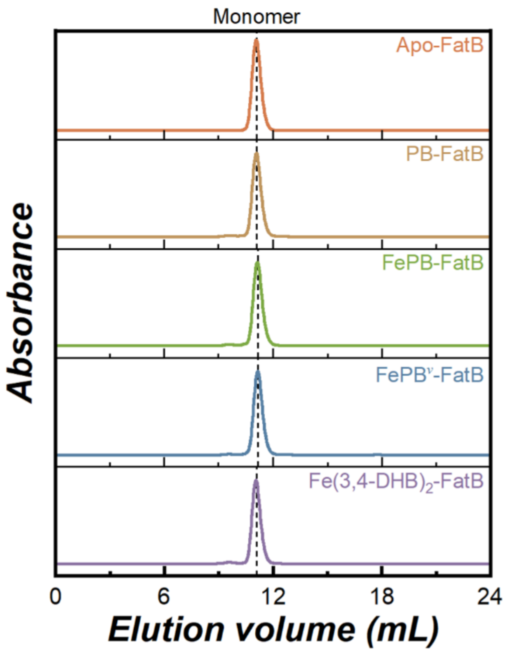

Supplementary Figure 8. Size-exclusion chromatography profiles of FatB complexes used for SAXS measurements. Each sample eluted as a single, symmetric peak, indicating monodispersity and the absence of aggregation. The elution volumes correspond to monomeric FatB for all apo and ligand-bound states.

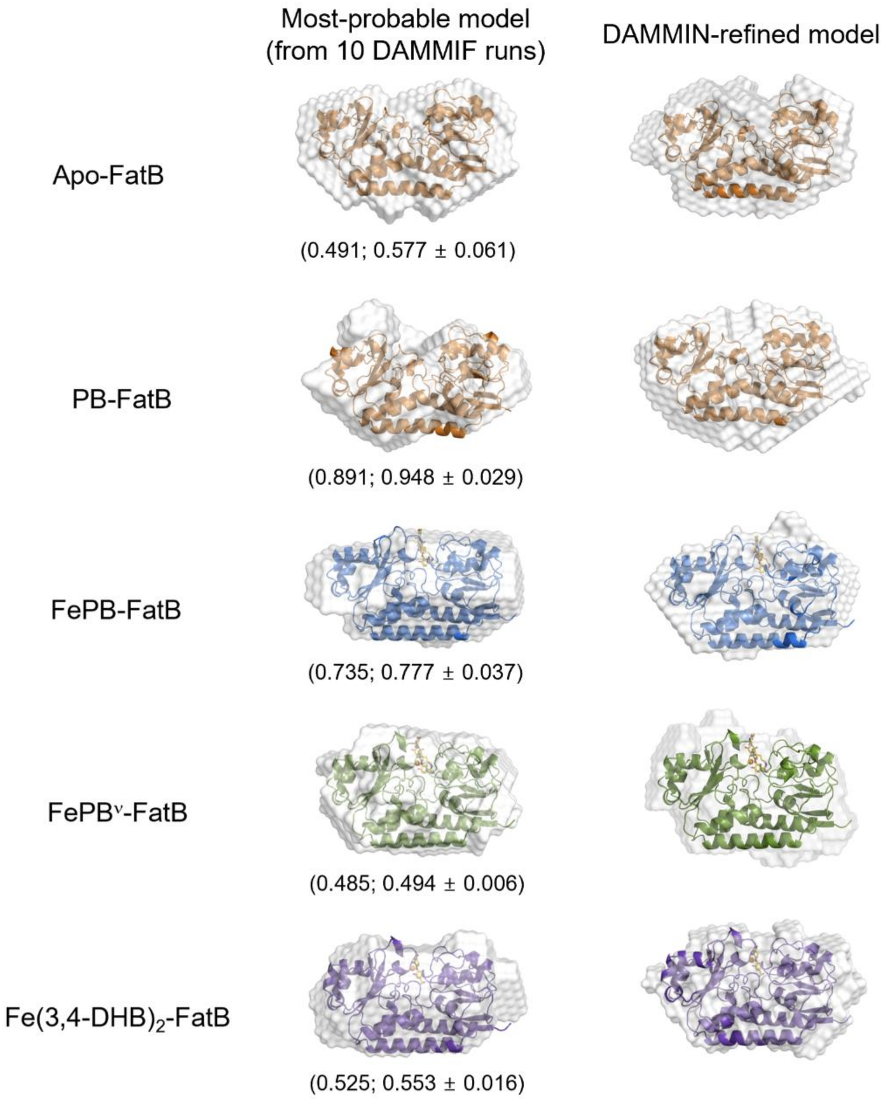

Supplementary Figure 9. Most-probable and DAMMIN-refined ab initio bead models for FatB states[4,5]. For each state (apo-FatB, PB-FatB, FePB-FatB, FePB^{v}-FatB, and Fe(3,4-DHB)$_2$-FatB), 10 independent ab initio reconstructions were generated using DAMMIF assuming P1 symmetry[4]. Left, the most-probable model (DAMSEL-selected) defined as the reconstruction with the smallest mean NSD after DAMSUP superposition of the ensemble. Right, the corresponding DAMMIN-refined model obtained by refining each reconstruction against the experimental scattering profile (Refine with DAMMIN option)[5]. In each panel, the bead-model envelope is shown in gray and superposed with the crystal structure colored as apo-FatB (orange), FePB-FatB (blue), FePB^{v}-FatB (green), and Fe(3,4-DHB)$_2$-FatB (purple) (PB-FatB is superposed with the same best-matching crystal structure used for Fig. 3a). Numbers in parentheses report the NSD of the selected model and the ensemble mean ± standard deviation (SD). These additional overlays are provided to document the range of

acceptable representations and to allow readers to directly assess the agreement between the solution envelopes and the corresponding crystal structures.

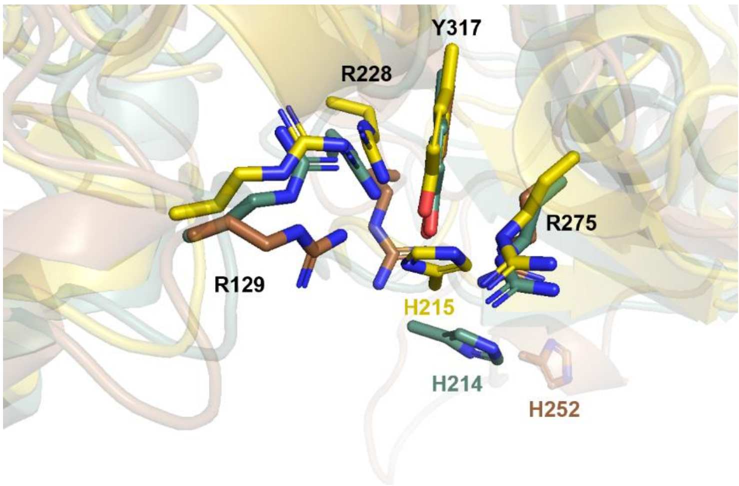

Supplementary Figure 10. Superposition of apo-FatB (brown) with apo-Pth (yellow) and apo-YclQ (green) showing conserved binding-cleft residues[1,2]. The corresponding histidines (Pth His215, YclQ His214 and FatB His252) are individually labeled to distinguish their positions, and other conserved binding-cleft residues are annotated with the residue numbers of the FatB sequence.

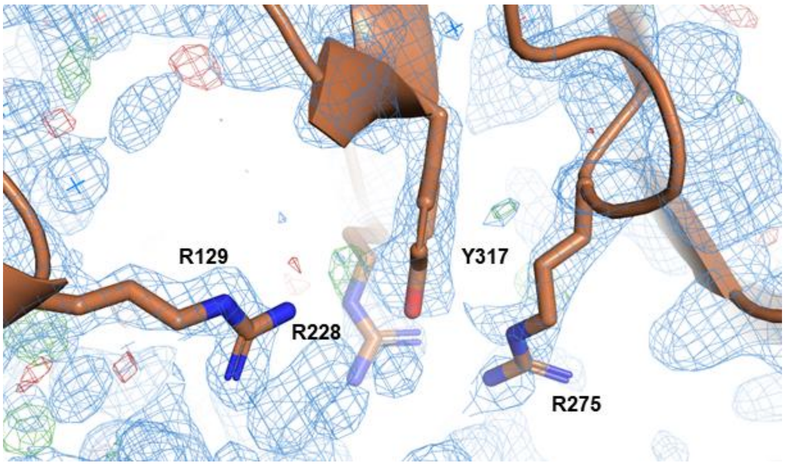

Supplementary Figure 11. Representative electron density map in the binding-cleft region of apo-FatB. A close-up view of the apo-FatB binding cleft is shown with the refined model overlaid on the electron density map. The map is displayed as a $2mF_o$–DF_c map contoured at 1.0 σ, with the corresponding mF_o–DF_c map shown at ±3.0 σ

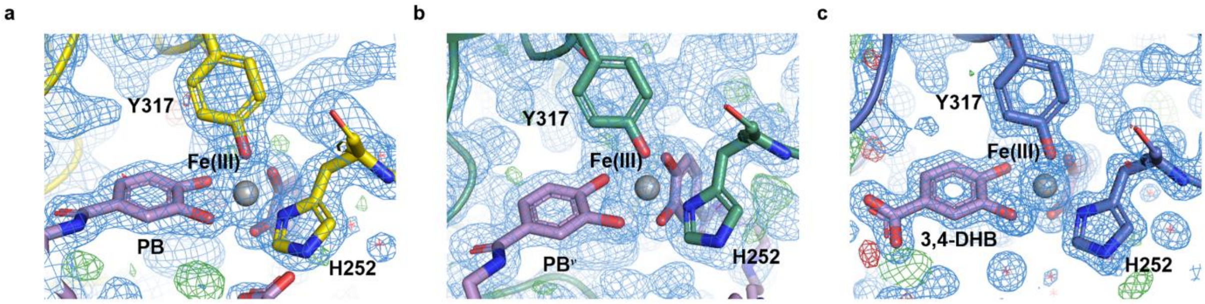

Supplementary Figure 12. Close-up electron density maps around the Fe(III) coordination sphere in ferric-ligand-bound FatB. These electron density maps highlight continuous $2mF_o–DF_c$ density for all Fe(III)-coordinating residues (protein-colored sticks), ligands (purple sticks) and Fe(III) ion (gray sphere). (**a**) FePB-FatB (yellow), (**b**) FePB^{v}-FatB (green), and (**c**) Fe(3,4-DHB)$_2$-FatB (blue). Maps are shown at $2mF_o–DF_c$ (blue, 1.0 σ) and $mF_o–DF_c$ (green/red, ± 3.0 σ) contour levels.

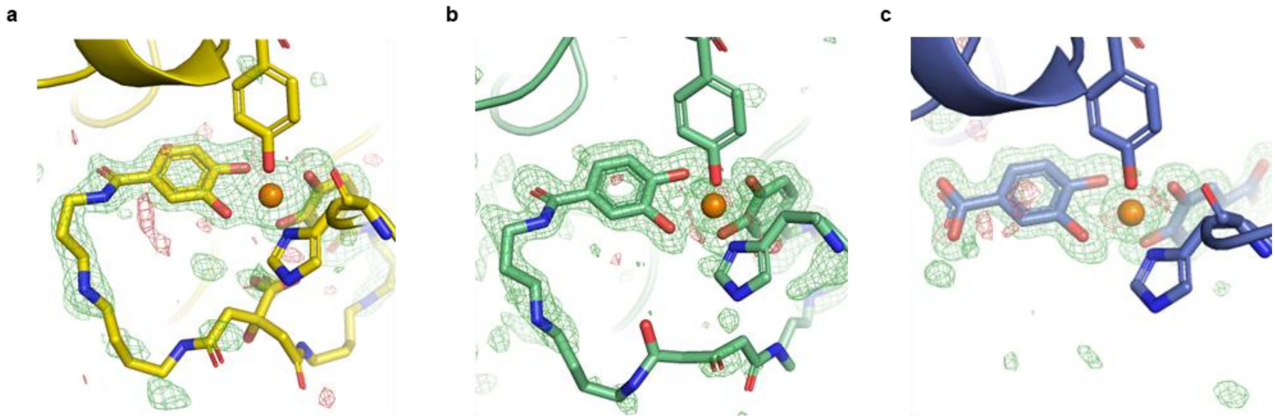

Supplementary Figure 13. Ligand omit maps for ferric ligand-bound FatB complexes. *mFo–DFc* omit maps are shown for (a) FePB-FatB (yellow), (b) $FePB^n$-FatB (green), and (c) $Fe(3,4\text{-}DHB)_2$-FatB (blue). For each complex, the ferric ligand was omitted from the model prior to refinement. The resulting positive omit density is shown as green mesh contoured at +3.0 σ and the resulting negative omit density is shown as red mesh contoured at -3.0 σ. Final refined ligand models are shown as sticks and Fe(III) ions as orange spheres.

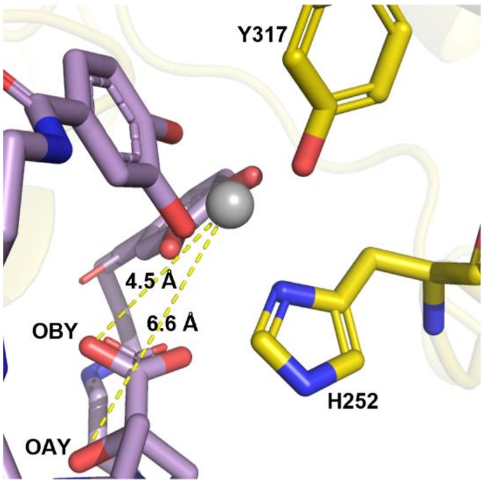

Supplementary Figure 14. Crystallographic validation of the Tyr/His coordination sphere in the FePB-FatB complex. The Final refined model of the FePB-FatB binding site is shown, with the ligand depicted as purple sticks and the Fe(III) ion as a gray sphere. In free FePB, the ligand's α-hydroxycarboxylate oxygens (OAY and OBY) serve as Fe(III)-coordinating donors, but in the FePB-FatB structure, they are located at non-coordinating distances of 4.5 Å and 6.6 Å respectively, from the metal center, consistent with Fe(III) being ligated instead by Tyr317 and His252.

a b

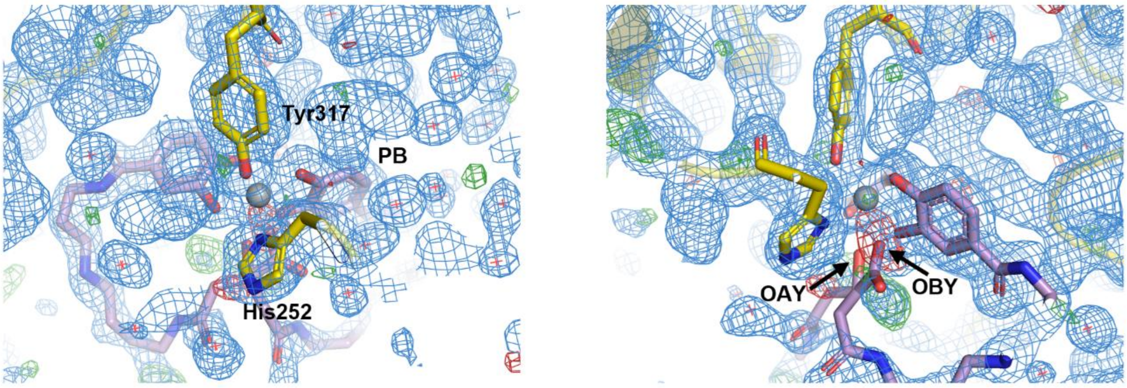

Supplementary Figure 15. Electron density maps of the α-hydroxycarboxylate-retained alternative FePB-FatB model. (**a**) The $2mF_o–DF_c$ map (blue, contoured at 1.0 σ) and $mF_o–DF_c$ map (green/red, contoured at ±3.0 σ) are shown for the refinement model where the ligand's α-hydroxycarboxylate group (OAY and OBY) was forced to coordinate the Fe(III) ion. (**b**) A 70° rotated view of the same site. The maps show an absence of supporting positive (green) $mF_o–DF_c$ density at the modeled α-hydroxyl oxygen (OAY) and, strong negative (red) $mF_o–DF_c$ density on the modeled α-carboxylate oxygen (OBY), demonstrating this alternative model is not supported by the data.

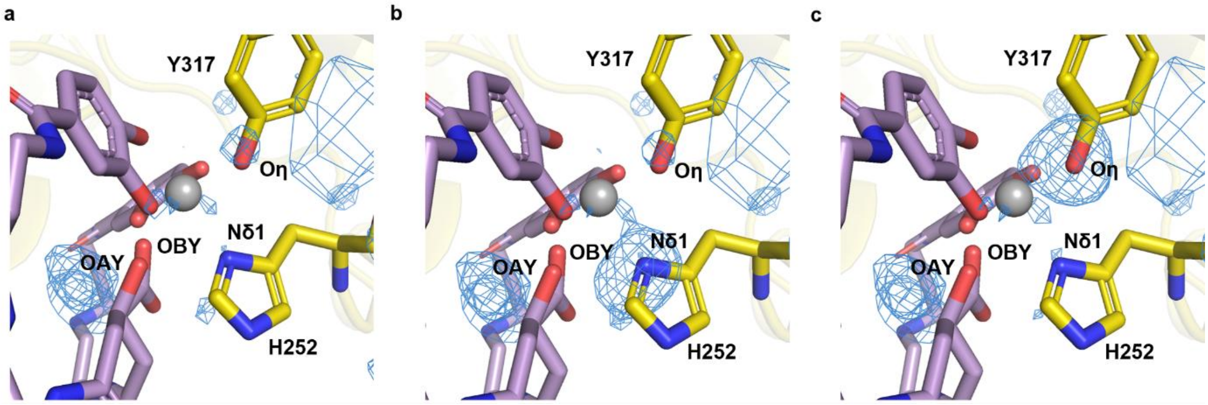

Supplementary Figure 16. Polder omit maps (blue mesh, contoured at +3.0 σ) calculated from an alternative model where the ligand's α-hydroxycarboxylate group was enforced to coordinate the Fe(III) ion[6]. (**a**) The Polder map calculated for the omitted OAY atom shows an absence of electron density at its modeled position. (**b**) The map calculated for the omitted His252 residue shows strong and continuous density at the Nδ1 atom. (**c**) The Polder map calculated for the omitted Tyr317 residue also shows strong and continuous density at the Oη atom. Together, these maps confirm that the Tyr317/His252 pair, and not the ligand's α-hydroxycarboxylate, coordinates the Fe(III) ion.

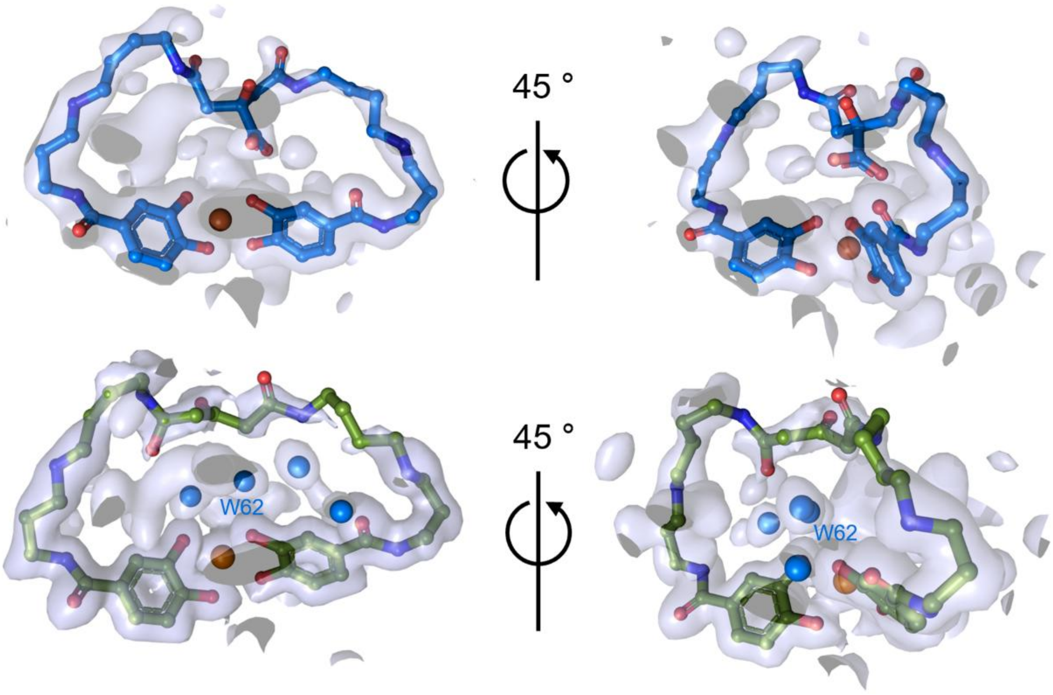

Supplementary Figure 17. Electron-density comparison of FePB and FePB^{v} in the FatB binding site. Representative $2mF_o–DF_c$ electron-density maps (gray semitransparent surfaces) are shown around the ferric ligand bound in the FePB-FatB (top, PB; blue sticks) and FePB^{v}-FatB (bottom, PB^{v}; green sticks) complexes. For each complex, the ligand region is displayed in two orthogonal views (right panels rotated 45° relative to the left). In the FePB-FatB structure, the density follows the carboxylate-bearing arm of FePB, consistent with the intact ligand model. In the FePB^{v}-FatB structure, the density expected for the corresponding carboxylate group is absent, consistent with assignment of the decarboxylated photoproduct. Notably, the ordered water W62, which occupies the cavity adjacent to the ligand in the FePB^{v}-binding interface, is spatially separated from the ligand density. To allow comparison of local density under identical visualization conditions, all maps in this figure are contoured at 0.75σ. The Fe(III) ion is shown as an orange sphere, and ordered solvent molecules associated with the FePB^{v}-binding interface are shown as blue spheres.

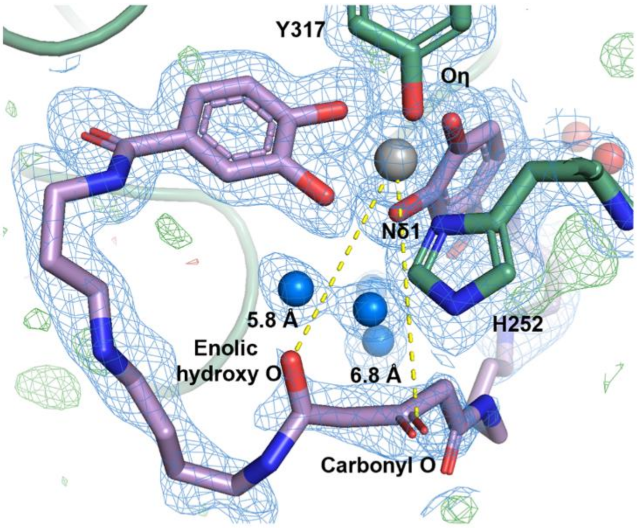

Supplementary Figure 18. Electron density maps of FePB^{v}-FatB around the Fe(III) center, contoured at 1.0 σ for the 2mF_o–DF_c map (blue) and ±3.0 σ for the mF_o–DF_c map (green/red). Continuous density from Fe(III) to Tyr317 Oη and His252 Nδ1 are shown around the Fe(III) site, with no positive residual density consistent with any additional enolic/carbonyl donor near the metal. The cavity left by photodecarboxylation is filled by three ordered waters, W339, W62, W171, W147, each supported by contiguous 2mF_o–DF_c density. Dashed lines indicate the non-coordinating separations from Fe to the enolic O (~5.8 Å) and adjacent carbonyl O (~6.8 Å) of FePB^{v}. Together, these data support that PB^{v} does not supply the proposed enolic/carbonyl pair in the FatB pocket; instead, Tyr317/His252 provide the protein-derived first coordination sphere.

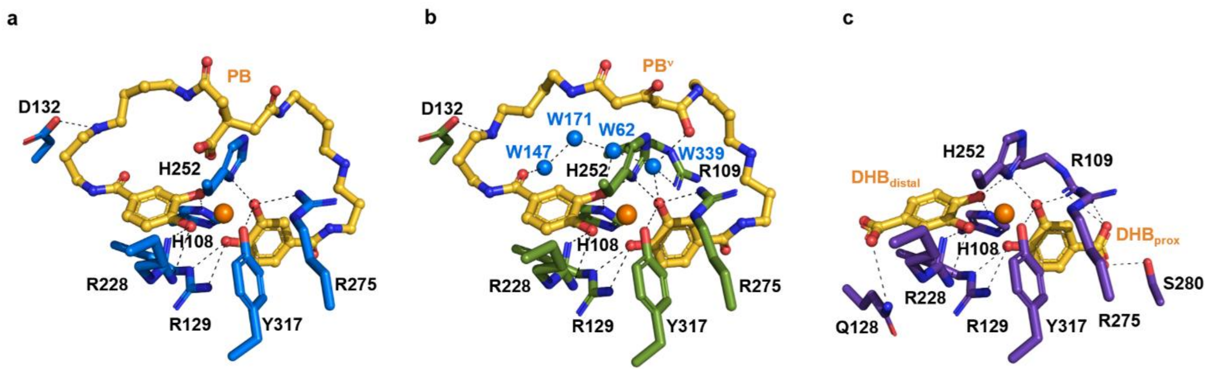

Supplementary Figure 19. Hydrogen-bonding networks in the FatB binding clefts. Detailed views of the binding clefts of (a) FePB-FatB, (b) FePB^{v}-FatB, and (c) Fe(3,4-DHB)$_2$-FatB showing the hydrogen-bonding interactions around the ligands. Hydrogen bonds are indicated by black dashed lines. Ligands are shown as yellow sticks, and Fe(III) ions as orange spheres. Protein residues are shown as sticks and colored blue, green, and purple in panels a, b, and c, respectively. Ordered water molecules in the FePB^{v}-FatB complex are shown as blue spheres and labeled (W147, W171, W62, and W339).

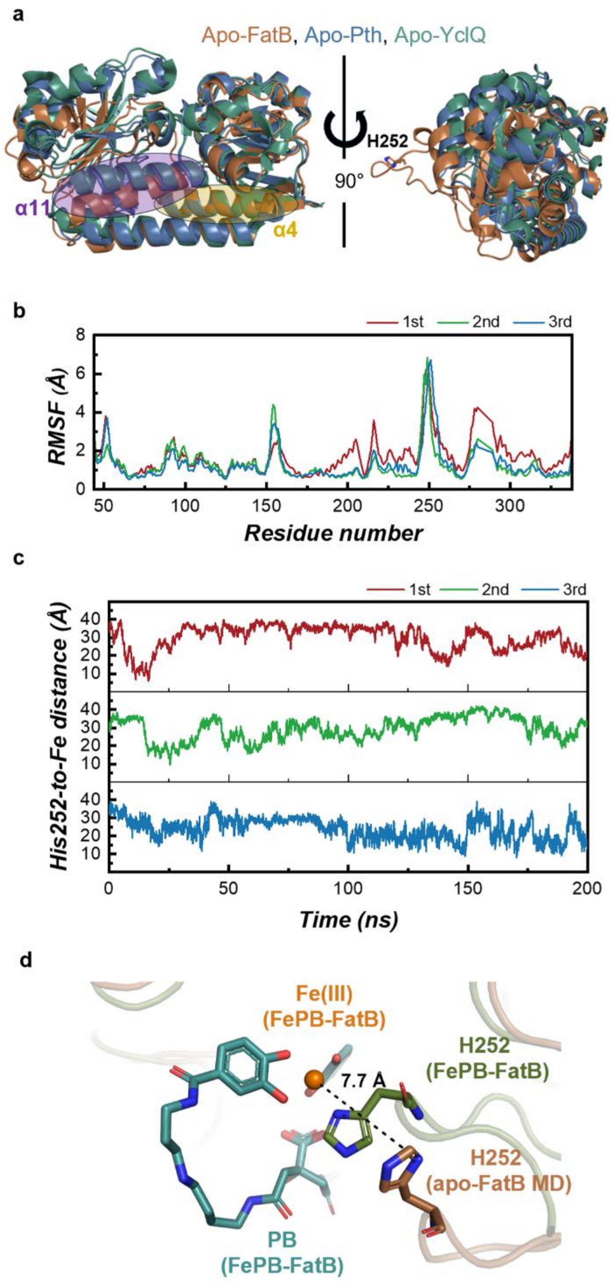

Supplementary Figure 20. Structural and dynamic basis of hinge flexibility and loop mobility in apo-FatB. (**a**) Superposition of apo-FatB (brown), apo-Pth (blue), and apo-YclQ (green) aligned on the N-terminal domain. The left view shows that C-terminal helix α11 (brown, within the purple-highlighted region) in apo-FatB is oriented less parallel to the N-terminal helix α4 (brown within the yellow-highlighted region) than in homologs, resulting in looser hinge packing. The right view (rotated 90°) highlights the outward-protruding loop (residues 244–254) containing His252 (shown as sticks). (**b**) RMSF profiles computed over the production window (50–250 ns; 200 ns) from three independent 250-ns MD simulations of apo-FatB, showing highest

flexibility in the His252-containing loop. (**c**) Time-evolution of the center-of-mass distance between the His252 imidazole ring and the Fe(III) position in FePB-FatB, defined from the FePB-FatB structure, shown for three independent production trajectories (50–250 ns; plotted as 0–200 ns after removing the initial 50 ns). The traces exhibit intermittent decreases to binding-proximal distances (~10 Å). (**d**) Representative MD snapshot showing the closest approach of His252, aligned with the FePB-FatB crystal structure.

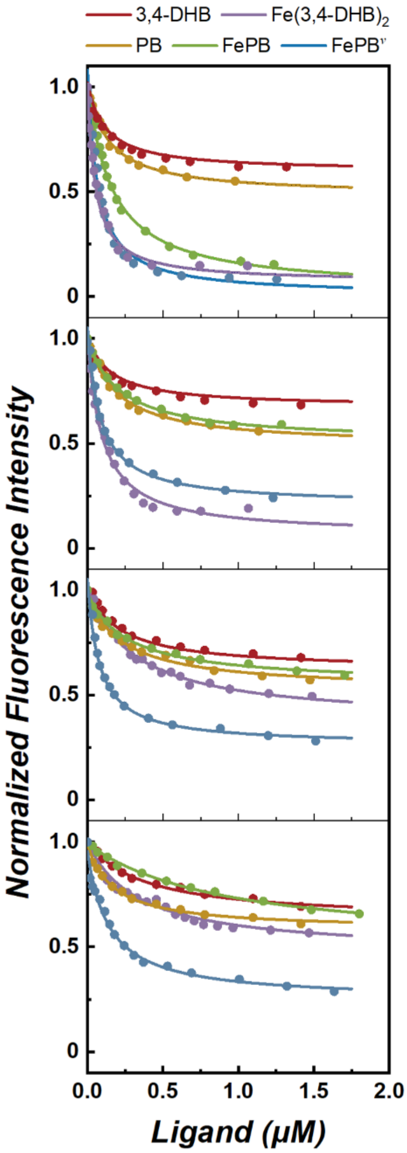

Supplementary Figure 21. Fluorescence quenching analysis of FatB variants with ligands. Tryptophan fluorescence quenching titrations were performed for four FatB constructs: WT, H252A, Y317F, and H252A/Y317F. Each protein was titrated with five ligands, 3,4-DHB (red), and $Fe(3,4\text{-}DHB)_2$ (purple), PB (yellow), FePB (green), $FePB^v$ (blue), and fitted in DYNAFIT using a one-site binding model to obtain dissociation constants (K_d)[7]. Solid lines represent best-fit curves, and data points denote experimental measurements.

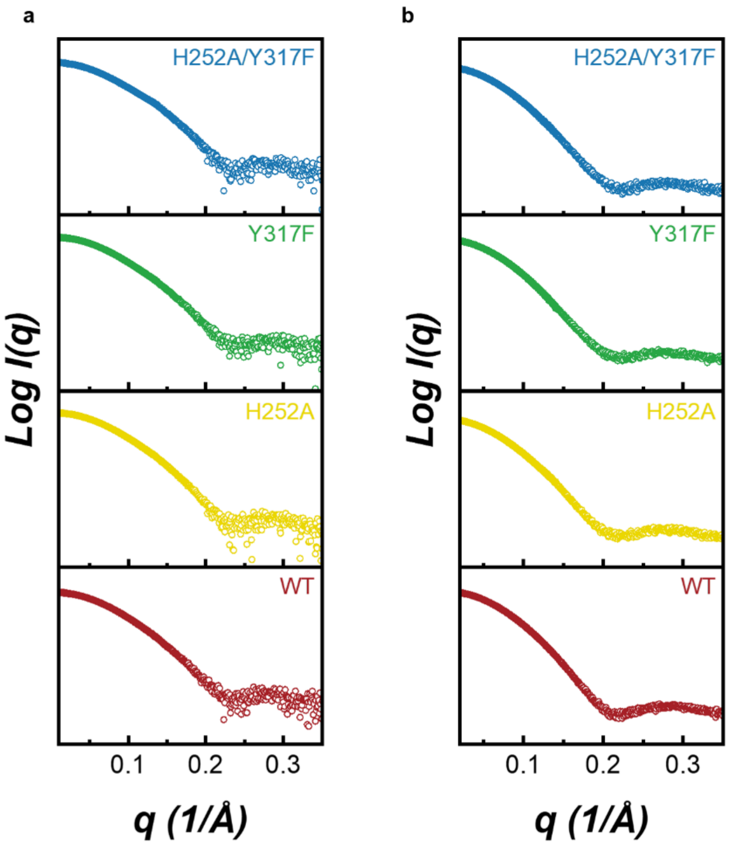

Supplementary Figure 22. SAXS analysis of FatB variants in apo and FePB-bound states. Experimental solution scattering profiles are shown for wild-type (WT) and the H252A, Y317F, and H252A/Y317F mutants. (a) Profiles for the apo-FatB constructs. (b) Profiles for the corresponding FePB-bound constructs. The scattering intensity is plotted on a logarithmic scale as a function of the scattering vector, *q*.

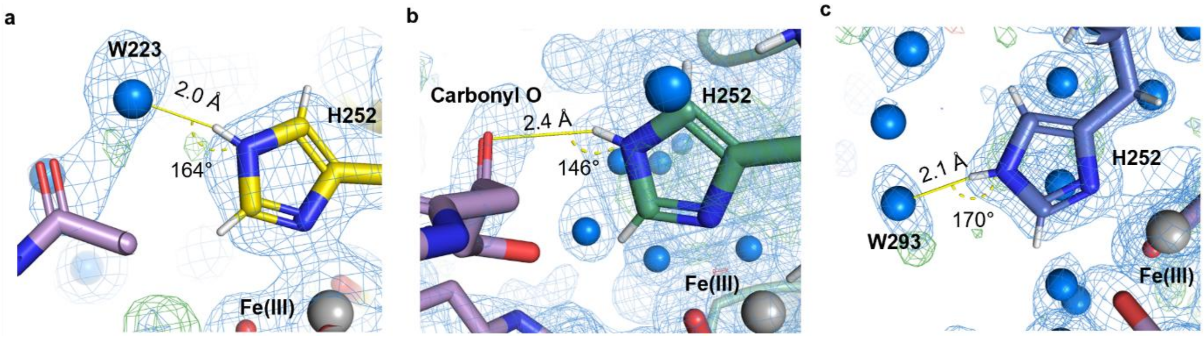

Supplementary Figure 23. His252 δ-coordination stabilized by Nε2–H···O hydrogen bonds across all FatB complexes bound to ferric ligands. His252 donates a hydrogen bond via Nε2 in three FatB complexes. (**a**) FePB-FatB: Nε2–H···O(W223) (**b**) FePB^{v}-FatB: Nε2–H···O (the carbonyl oxygen of PB^{v}) (**c**) Fe(3,4-DHB)$_2$-FatB: Nε2–H···O(W293). Protein hydrogen atoms were added using the program Reduce (as implemented in the PHENIX package)[8] for analysis only; water hydrogens were not modeled. His252 was fixed as the HIE tautomer (Nε2–H donor, Nδ1 metal ligand). Electron density is shown as $2mF_o–DF_c$ at 1.0 σ (blue mesh) and $mF_o–DF_c$ at ±3.0 σ (green/red mesh). Each panel annotates the Nε2–H···O distance and Nε2–H···O angle, consistently indicating a donor-type hydrogen bond from Nε2 in all three FatB complexes, thereby supporting Nδ1 coordination of His252 to Fe(III). Deposited coordinates do not include hydrogens; angles/distances were measured on the hydrogenated analysis models.

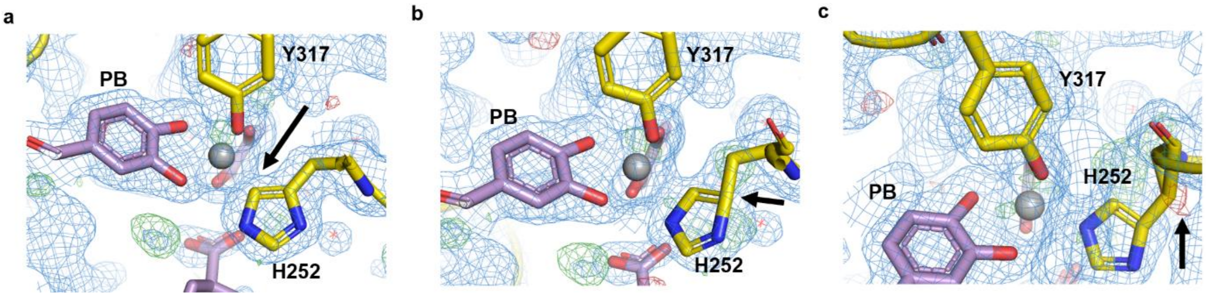

Supplementary Figure 24. Modeling trials demonstrating that His252 coordinates Fe(III) via Nδ1, not Nε2. Electron density maps are $2mF_o–DF_c$ at 1.0σ (blue mesh) and $mF_o–DF_c$ at ±3.0σ (green/red mesh). The protein is shown in yellow sticks, the ligand in purple sticks, and the Fe(III) ion as a gray sphere. (**a**) Unrestrained refinement of an alternative conformation. The His252 side chain was manually reoriented to place its Nε2 atom toward the Fe(III) ion. In the subsequent unrestrained refinement, however, the imidazole ring pivoted, bringing a carbon edge, not the Nε2 atom, closest to the metal center. (**b**) Geometrically strained model with a forced Fe–Nε2 link. An explicit Fe–Nε2 LINK restraint was imposed during refinement. This forced the side chain into a chemically implausible geometry, resulting in a Cβ–Cγ–Nδ1 angle of 177.7°, flagged by MolProbity as a 38σ outlier. (**c**) Emergence of strong difference density peaks. Continued refinement of the restrained model resulted in large positive and negative $mF_o–DF_c$ peaks. The Cβ atom was modeled into a region lacking $2mF_o–DF_c$ support, creating a large negative (red) peak at its new position, while a large positive (green) peak appeared at its original, stable site. Together, these modeling trials show that enforcing an Fe–Nε2 coordination geometry results in severe geometric distortions and strong residual electron density, confirming that His252 exclusively coordinates the Fe(III) ion via its Nδ1 atom in the native structure.

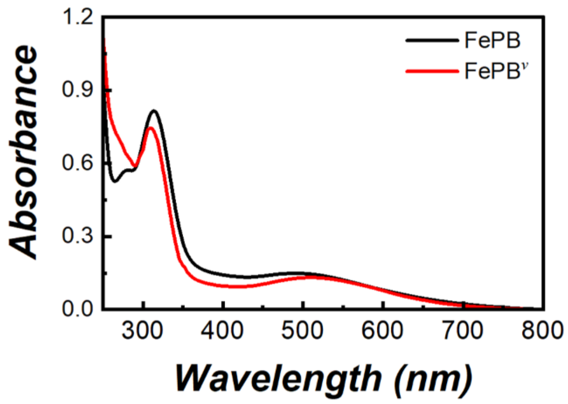

Supplementary Figure 25. UV–visible absorption spectra of FePB (black) and FePB^{v} (red) measured at 0.06 mM in 20-mM Tris–HCl (pH 8.0).

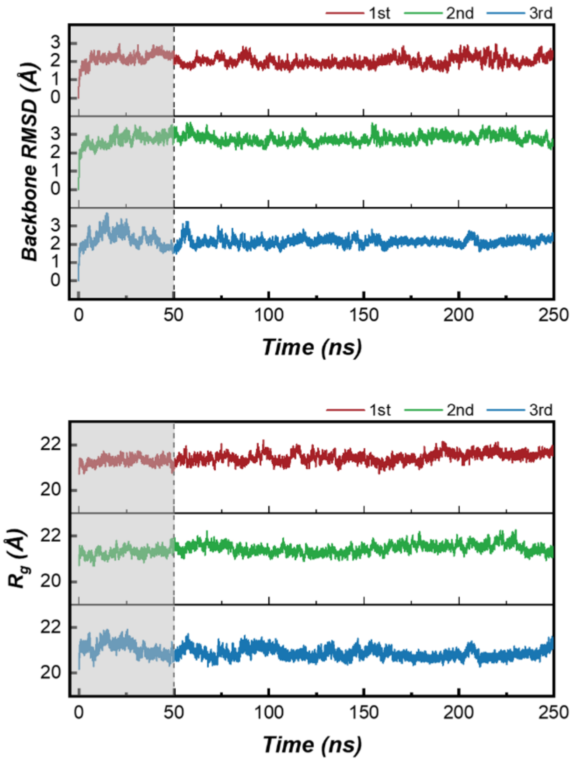

Supplementary Figure 26. Convergence assessment of apo-FatB MD simulations. Time evolution of (top) backbone RMSD and (bottom) R_g for three independent apo-FatB MD replicas (1st–3rd). Backbone RMSD was computed relative to the starting structure after least-squares fitting to the N-terminal domain (residues 44–181) of FePB-FatB, and R_g was calculated for the protein. The gray shaded region (0–50 ns) indicates the equilibration period, which was excluded from downstream analyses. After ~50 ns, all replicas display stable RMSD and R_g without systematic drift, supporting the use of the subsequent 200-ns window (50–250 ns) as the production segment for quantitative analysis.

Supplementary Table 1. Oligonucleotide primers used for cloning and site-directed mutagenesis of *Bc*FatB. The first two primer pairs were used to introduce mutations into the *FatB* wild-type (WT) construct. The following two pairs were used for generating single mutants (H252A and Y317F), and the double mutant (H252A/Y317F) was obtained by introducing the Y317F mutation into the H252A background.

Primer name	Sequence (5' → 3')
FatB_40F_LIC_for	TACTTCCAATCCAATGCAAGTGATAAGCCAAAAACGGTTGAAATC
FatB_338R_LIC_rev	TTATCCACTTCCAATGTTATTACTTAGCTAAAGTTTTTGCCATATTTCCA
FatB_40F_H252A_for	GTTTCAAATTCTACTGCAGGTGCTAAAGGTGATGACGTTTCTGTTG
FatB_40F_H252A_rev	CAACAGAAACGTCATCACCTTTAGCACCTGCAGTAGAATTTGAAAC
FatB_40F_Y317F_for	CAAGTTATTTATGCACCAGAAGATACTTTCACGAATGAATCAATTC
FatB_40F_Y317F_rev	TTGAATTGATTCATTCGTGAAAGTATCTTCTGGTGCATAAATAACTTG

Supplementary Table 2. X-ray data collection and refinement statistics.

	Apo-FatB	FePB-FatB	FePB^{v}-FatB	Fe(3,4-DHB)$_2$-FatB
**PDB ID**	21ZD	21ZE	21ZF	21ZG
**Data collection**				
Space group	$P2_12_12_1$	$P2_1$	$P2_1$	$P2_1$
Cell dimensions				
a, *b*, *c* (Å)	36.4, 57.8, 100.9	39.7, 68.0, 50.6	39.6, 66.5, 50.1	41.4, 66.7, 50.5
α, β, γ (°)	90, 90, 90	90, 92.1, 90	90, 91.7, 90	90, 90.4, 90
Resolution (Å)	38.0 – 1.91 (1.98 – 1.91)	40.6 – 1.79 (1.85 – 1.79)	40.01 - 1.58 (1.64 - 1.58)	41.44 – 1.40 (1.45 – 1.40)
R_{merge}	0.346 (1.062)	0.756 (1.389)	0.083 (0.254)	0.116 (0.561)
$I / \sigma I$	13.5 (2.2)	41.1 (11.1)	14.2 (3.9)	20.2 (3.8)
Completeness (%)	95.0 (85.6)	98.0 (96.2)	97.7 (95.2)	97.6 (92.0)
Redundancy	5.5 (2.9)	21.2 (18.3)	4.0 (3.9)	6.6 (5.8)
**Refinement**				
No. reflections	16974 (1430)	25529 (2448)	34799 (3320)	53132 (4938)
R_{work} / R_{free}	0.215 / 0.259	0.169 / 0.217	0.155 / 0.189	0.139 / 0.173
No. atoms				
Protein	2203	2292	2286	2253
Ligand/ion	0	68	49	31
Water	172	309	394	502
B-factors				
Protein	30.6	28.5	16.1	16.4
Ligand/ion	N/A	35.2	23.7	20.5
Water	33.1	35.2	28.3	31.1
R.m.s. deviations				
Bond lengths (Å)	0.003	0.009	0.010	0.006
Bond angles (°)	0.60	1.01	0.93	0.84

The values in parentheses are for highest-resolution shell.

Supplementary Table 3. Quantification of ligand-induced conformational changes in FatB. Comparison of interdomain rearrangements between apo-FatB and its ferric ligand-bound forms. The rotation angle of the C-terminal domain was determined following the method established in previous studies, by aligning the N-terminal domains and measuring the relative motion of the C-terminal domain. The straightening angle of the central α-helix was calculated as the angle between vectors connecting residue 196 (where divergence begins) and residue 202 (helix terminus). For the main text, values are reported as mean ± SD across the three ferric-ligand-bound complexes (FePB-FatB, $FePB^{v}$-FatB, and $Fe(3,4\text{-}DHB)_2$-FatB).

Complex	Rotation angle of C-terminal domain	Straightening angle of central α-helix	Distance shortening between COM of domains
FePB-FatB	14.4°	8.3°	3.0 Å
$FePB^{v}$-FatB	15.0°	8.2°	3.0 Å
$Fe(3,4\text{-}DHB)_2$-FatB	14.0°	8.3°	3.1 Å
Mean ± SD	14.5 ± 0.5°	8.3 ± 0.1°	3.0 ± 0.1 Å

Supplementary Table 4. Pairwise Cα RMSD analysis of FatB complexes. Root-mean-square deviations (RMSDs, Å) were calculated for Cα atoms across the entire protein ($RMSD_{Total}$), the N-terminal domain ($RMSD_N$), and the C-terminal domain ($RMSD_C$) after optimal superposition of the corresponding domains. Only unique structure pairs are listed. All ferric-ligand-bound states exhibit minimal deviations from one another (≤ 0.3 Å), consistent with a shared closed conformation. In contrast, apo-FatB shows significantly larger RMSDs relative to the holo complexes, reflecting its open conformation.

Complex 1	Complex 2	$RMSD_{Total}$	$RMSD_N$	$RMSD_C$
Apo-FatB	FePB-FatB	1.82	0.60	0.46
Apo-FatB	$FePB^v$-FatB	1.79	0.61	0.45
Apo-FatB	$Fe(3,4\text{-}DHB)_2$-FatB	1.67	0.61	0.49
FePB-FatB	$FePB^v$-FatB	0.18	0.15	0.17
FePB-FatB	$Fe(3,4\text{-}DHB)_2$-FatB	0.22	0.19	0.22
$FePB^v$-FatB	$Fe(3,4\text{-}DHB)_2$-FatB	0.20	0.18	0.17

Supplementary Table 5. Details for SAXS measurements and data analysis.

(*a*) Sample details					
	Apo-FatB	PB-FatB	FePB-FatB	$\mathrm{FePB^{v}}$-FatB	Fe(3,4-DHB)$_2$-FatB
Organism	*Bacillus cereus*				
Source (Catalogue No. or reference)	*E. coli* expressed (This study)				
Description: sequence (including Uniprot ID + uncleaved tags), bound ligands/modifications, *etc.*	Q815N5 (40-338), no ligands	Q815N5 (40-338), PB-bound	Q815N5 (40-338), FePB-bound	Q815N5 (40-338), $\mathrm{FePB^{v}}$-bound	Q815N5 (40-338), Fe(3,4-DHB)$_2$-bound
Extinction coefficient ε (wavelength and units)	ε_{280} = 32430				
Partial specific volume $\bar{\upsilon}$ (cm^3 g^{-1})	0.7401				
Molecular mass *M* from chemical composition (Da)	32698				
Concentration	0.1 mM				
Solvent composition and source	20 mM Tris-HCl, pH 8.0, 50 mM NaCl				
(*b*) SAS data collection parameters					
	Apo-FatB	PB-FatB	FePB-FatB	$\mathrm{FePB^{v}}$-FatB	Fe(3,4-DHB)$_2$-FatB
Source	4C beamline at Pohang Accelerator Laboratory storage ring (Pohang, South Korea) using a Dectris / EIGER2 X 4M detector				
Wavelength (Å)	0.734				
Beam geometry	Size (Width, Length): 400 μm, 250 μm<br>Sample-to-detector distance: 1 m, 3 m				1 m, 4 m
q-measurement range ($Å^{-1}$)	0.025–0.60				0.014–0.60
Method for monitoring radiation damage	Guinier analysis				
Exposure time, number of exposures	Exposure time: 10 seconds, Number of exposures: 50				
Sample configuration	Path length: 1 mm, Flow rate: 15 μl/min				
Sample temperature (ºC)	4 ˚C				
(*c*) Software employed for SAS data reduction, analysis and interpretation					
	Apo-FatB	PB-FatB	FePB-FatB	$\mathrm{FePB^{v}}$-FatB	Fe(3,4-DHB)$_2$-FatB
SAS data reduction to sample–solvent scattering, and merging	PRIMUS[9]				
Calculation of ε from sequence	ProtParam[10]				
Calculation of $\bar{\upsilon}$ values from chemical composition	SEDNTERP[11]				
Basic analyses: Guinier, *P*(*r*), scattering particle volume	GNOM[12]				
Shape/bead modelling	DAMMIF[4], DAMMIN[5]				
Atomic structure modelling	CRYSOL[13]				
Molecular graphics	PyMOL				

(*d*) Structural parameters					
	Apo-FatB	PB-FatB	FePB-FatB	$FePB^{v}$-FatB	Fe(3,4-DHB)$_2$-FatB
Guinier Analysis					
$I(0)$ (a. u.)	84.2	83.2	73.5	76.8	67.9
R_g (Å)	22.1	22.1	21.2	21.4	21.0
q-range (Å^{-1})	0.0254–0.0593	0.0250–0.0589	0.0260–0.0613	0.0262–0.0609	0.0176–0.0630
qR_g max	1.3	1.3	1.3	1.3	1.3
$P(r)$ analysis					
$I(0)$ (a. u.)	84.57	83.4	73.6	77.1	67.5
R_g (Å)	22.1	22.1	21.3	21.5	21.0
d_{max} (Å)	73.5	74.8	70.7	71.3	71.2
q-range (Å^{-1})	0.0254–0.3714	0.0250–0.3740	0.0251–0.3897	0.0250–0.3929	0.0149–0.3396
Total Estimate	0.76	0.86	0.82	0.82	0.77

(*e*) Shape modelling results (a complete panel for each method)					
	Apo-FatB	PB-FatB	FePB-FatB	$FePB^{v}$-FatB	Fe(3,4-DHB)$_2$-FatB
DAMMIF					
q-range for fitting	0.025–0.365	0.025–0.363	0.025–0.379	0.025–0.377	0.014–0.322
Symmetry	P1	P1	P1	P1	P1
NSD (standard deviation)	0.50 (0.01)	0.95 (0.03)	0.78 (0.04)	0.57 (0.01)	0.55 (0.02)
χ^2 range	0.77–0.97	0.75–0.84	1.95–2.16	1.28–1.36	0.50–0.60
Model resolution (Å)	20	33	25	17	20
DAMMIN					
q-range for fitting	0.025–0.365	0.025–0.363	0.025–0.379	0.025–0.377	0.014–0.322
Symmetry	P1	P1	P1	P1	P1
χ^2 value	0.85	0.87	1.05	0.94	0.75

(*f*) Atomistic modelling					
	Apo-FatB	PB-FatB	FePB-FatB	$FePB^{v}$-FatB	Fe(3,4-DHB)$_2$-FatB
CRYSOL					
Crystal structures	Apo-FatB (21ZD)	Apo-FatB (21ZD)	FePB-FatB (21ZE)	$FePB^{v}$-FatB (21ZF)	Fe(3,4-DHB)$_2$-FatB (21ZG)
q-range for fitting	0.025–0.350	0.025–0.350	0.025–0.350	0.025–0.350	0.014–0.350
χ^2 value	0.99	1.07	1.03	0.99	0.97
Predicted R_g (Å)	21.5	21.5	20.6	20.8	20.4
Vol (Å^3), Dro (e Å^{-3})	39328, 0.036	39328, 0.024	41652, 0.034	43643, 0.033	40766, 0.062

(*g*) Data and model deposition IDs					
	Apo-FatB	PB-FatB	FePB-FatB	$FePB^{v}$-FatB	Fe(3,4-DHB)$_2$-FatB
	SASDYB7	SASDYC7	SASDYD7	SASDYE7	SASDYF7

Supplementary Table 6. Goodness-of-fit (χ^2) values from CRYSOL analysis comparing experimental SAXS profiles to theoretical crystal structure models. Experimental SAXS profiles (rows) were compared against theoretical scattering curves calculated from the four available crystal models (columns). The lowest χ^2 values (approximately 1.0, highlighted in gray) are found along the diagonal, indicating that the SAXS profile for each state (Apo-FatB, FePB-FatB, FePB^{v}-FatB, and Fe(3,4-DHB)$_2$-FatB) is best described by its own (cognate) crystal structure. The SAXS profile for PB-FatB, which lacks a crystal model, is also best fit by the open apo-FatB model. Fitting the open apo-FatB model to any of the closed ferric complex SAXS datasets results in poor fits (high χ^2 values), as does fitting any of the closed crystal models to the open apo-FatB SAXS data. The closed models show relatively good cross-compatibility ($0.9 < \chi^2 < 1.0$) with other closed state profiles.

SAXS profile	$\chi^2_{Apo\text{-}FatB}$	$\chi^2_{FePB\text{-}FatB}$	$\chi^2_{FePBv\text{-}FatB}$	$\chi^2_{Fe(3,4\text{-}DHB)2\text{-}FatB}$
Apo-FatB	0.991	1.581	1.341	1.652
PB-FatB	1.072	1.362	1.268	1.360
FePB-FatB	2.198	1.025	0.948	1.033
FePB^{v}-FatB	3.488	0.923	0.987	0.927
Fe(3,4-DHB)$_2$-FatB	8.674	1.052	1.044	0.966

Supplementary Table 7. Structural parameters derived from SAXS data for FatB variants. The radius of gyration (R_g) and maximum particle dimension (D_{max}) were determined from Guinier analysis and the pair distance distribution function, *P(r)*, respectively, for each construct in both the apo and FePB-bound states.

Complex	R_g (Å)	D_{max} (Å)
Apo-FatB WT	22.1	73.5
Apo-FatB H252A	22.3	73.1
Apo-FatB Y317F	22.4	73.0
Apo-FatB H252A/Y317F	22.9	73.0
FePB-FatB WT	21.2	70.7
FePB-FatB H252A	21.3	70.0
FePB-FatB Y317F	21.2	71.6
FePB-FatB H252A/Y317F	21.4	71.6

Supplementary Table 8. Essential octahedral metrics for FePB and FePB-FatB. Octahedral parameters were extracted from the geometry-optimized structures used in the TD–DFT calculations. Listed are the mean Fe–ligand bond length (Mean Fe–L, ± standard deviation), the maximum absolute deviation of individual Fe–L distances from the mean (Max $|\Delta(\mathrm{Fe} - \mathrm{L})|$), the difference between the average axial and equatorial Fe–L distances (Axial–equatorial diff.), the largest deviations of cis and trans angles from 90° and 180°, respectively (Max cis / Max trans deviation), and the octahedral continuous shape measure CSM (O_h), where 0 corresponds to an ideal octahedral geometry. These metrics indicate that both complexes retain nearly ideal octahedral Fe(III) coordination, with FePB–FatB showing slightly larger bond-length and angular distortions consistent with protein-induced asymmetry.

	FePB	FePB-FatB
Mean Fe–L (Å)	2.03 ± 0.04	2.05 ± 0.10
Max $|\Delta(\mathrm{Fe} - \mathrm{L})|$ (Å)	0.08	0.20
Axial–equatorial diff. (Å)	0.06	0.17
Max cis deviation (°)	11.0	9.5
Max trans deviation (°)	16.9	11.5
CSM (O_h)	0.14	0.11

Supplementary Table 9. System setup for apo-FatB MD simulations.

System	Apo-FatB
Replicas	3
Force field / water	CHARMM36 / SPC/E
Box dimensions (nm)	8.88 × 8.88 × 8.88 (mean, 50–250 ns)
Total atoms	70,606
Water molecules (SOL)	22,061
Ions (#Na^+ / #Cl^-)	4 / 0
Salt concentration	No added salt; neutralizing counterions only (Na^+)
Lipids	None (0)

Supplementary Note 1. Excited-state analysis of the FePB-FatB complex.

For the spectral simulations, vertical excitation energies and oscillator strengths were computed using full time-dependent density functional theory (TD–DFT). Because the full TD–DFT formalism includes both excitation and de-excitation amplitudes, it yields transition densities that satisfy the relevant sum rules and properly account for interference effects between electronic configurations. This aspect is particularly important for analyses based on natural transition orbitals (NTOs) and the one-particle transition-density matrix (1TDM), which rely on a consistent representation of the transition density.

In contrast, the Tamm–Dancoff approximation (TDA) neglects the de-excitation channel and may produce transition densities that are less rigorously connected to the underlying electronic wavefunction. For this reason, all spectral simulations and excited-state analyses in this work were performed using the full TD–DFT formalism.

The excited-state character of each peak in the computed absorption spectra was analyzed using the TheoDORE 3 package[14-17] by evaluating the one-particle transition-density matrices (1TDMs) associated with each TD–DFT transition. Within this framework, the molecular system is partitioned into chemically meaningful fragments. In our case, the Fe(III) center and each coordinating ligand were treated as separate fragments, enabling decomposition of the TD–DFT eigenvectors over individual donor and acceptor groups.

The 1TDM is rewritten in this fragment-based representation to quantify both diagonal (local) and off-diagonal (charge-transfer) contributions. Fragment-based excited-state analyses of this type have been widely used to characterize charge-transfer states in complex molecular and condensed-phase systems[18-20].

This approach enables each excitation to be partitioned into metal-centered (MC), ligand-centered (LC), and charge-transfer (CT) components. The CT contributions were further classified into metal-to-ligand charge transfer (MLCT), ligand-to-metal charge transfer (LMCT), and ligand-to-ligand charge transfer (LLCT) channels. For each transition, the relative contributions of MC, LC, MLCT, LMCT, and LLCT character were evaluated and weighted by the corresponding oscillator strength to determine their contribution to the simulated absorption spectrum.

Natural transition orbitals (NTOs) were additionally analyzed to provide a compact orbital representation of the dominant electron–hole pairs associated with the most intense excitations. The resulting NTOs and the corresponding charge-transfer assignments are reported in Fig. 5 of the main text, where they support interpretation of the calculated absorption features.

Application of this analysis to free FePB shows that the lowest-energy absorption band (~492 nm) is dominated by catecholate→Fe(III) LMCT character. In contrast, the higher-energy region around ~300–320 nm, where FePB photodecarboxylation is typically induced, contains a heterogeneous mixture of LMCT and ligand-centered contributions.

When FePB binds to FatB, the calculations reproduce the experimentally observed red shift of the low-energy absorption band. The 1TDM analysis indicates that this shift does not originate from a change in the fundamental nature of the transition: in the FePB-FatB model the lowest-energy excitation remains predominantly catecholate→Fe(III) LMCT in character. Instead, modifications of the local coordination environment alter the relative energetic alignment of donor and acceptor states, leading to the observed spectral shift.

At higher excitation energies (~320 nm), an additional Tyr317→Fe LMCT contribution becomes apparent in the FePB-FatB model. This contribution redistributes the overall charge-transfer manifold and increases the ligand-to-ligand charge-transfer (LLCT) character in this energy range. Because this spectral region coincides with the excitation window where FePB photodecarboxylation is typically triggered, the calculations provide a mechanistic framework for understanding how the protein environment modulates the excited-state landscape of the FePB-FatB complex.

Supplementary References

1 Blagova, E. V. *et al.* Thermostable homologues of the periplasmic siderophore-binding protein CeuE from Geobacillus stearothermophilus and Parageobacillus thermoglucosidasius. *Acta Crystallogr. D* **79**, 694–705 (2023). https://doi.org/10.1107/S2059798323004473

2 Zawadzka, A. M. *et al.* Characterization of a Bacillus subtilis transporter for petrobactin, an anthrax stealth siderophore. *Proc. Natl. Acad. Sci. U. S. A.* **106**, 21854–21859 (2009). https://doi.org/10.1073/pnas.0904793106

3 Kabsch, W. & Sander, C. Dictionary of protein secondary structure: Pattern recognition of hydrogen-bonded and geometrical features. *Biopolymers* **22**, 2577–2637 (1983). https://doi.org/10.1002/bip.360221211

4 Franke, D. & Svergun, D. I. DAMMIF, a program for rapid ab-initio shape determination in small-angle scattering. *J. Appl. Cryst.* **42**, 342–346 (2009). https://doi.org/10.1107/S0021889809000338

5 Svergun, D. I. Restoring low resolution structure of biological macromolecules from solution scattering using simulated annealing. *Biophys. J.* **76**, 2879–2886 (1999). https://doi.org/10.1016/S0006-3495(99)77443-6

6 Liebschner, D. *et al.* Polder maps: improving OMIT maps by excluding bulk solvent. *Acta Crystallogr. D* **73**, 148–157 (2017). https://doi.org/10.1107/S2059798316018210

7 Kuzmič, P. Program DYNAFIT for the Analysis of Enzyme Kinetic Data: Application to HIV Proteinase. *Anal. Biochem.* **237**, 260–273 (1996). https://doi.org/10.1006/abio.1996.0238

8 Word, J. M., Lovell, S. C., Richardson, J. S. & Richardson, D. C. Asparagine and glutamine: using hydrogen atom contacts in the choice of side-chain amide orientation. *J. Mol. Biol.* **285**, 1735–1747 (1999). https://doi.org/10.1006/jmbi.1998.2401

9 Manalastas-Cantos, K. *et al.* ATSAS 3.0: expanded functionality and new tools for small-angle scattering data analysis. *J. Appl. Cryst.* **54**, 343–355 (2021). https://doi.org/10.1107/S1600576720 013412

10 Gasteiger, E. et al. Protein identification and analysis tools on the ExPASy server. in *The Proteomics Protocols Handbook* (ed. Walker, J. M.) 571–607 (Humana Press, Totowa, 2005). https://doi.org/10.1385/1-59259-890-0:571

11 Philo, J. S. SEDNTERP: a calculation and database utility to aid interpretation of analytical ultracentrifugation and light scattering data. *Eur Biophys J* **52**, 233–266 (2023). https://doi.org/10.1007/s00249-023-01629-0

12 Svergun, D. I. Determination of the regularization parameter in indirect-transform methods using perceptual criteria. *J. Appl. Cryst.* **25**, 495–503 (1992). https://doi.org/10.1107/S0021889892001663

13 Franke, D. *et al.* ATSAS 2.8: a comprehensive data analysis suite for small-angle scattering from macromolecular solutions. *J. Appl. Cryst.* **50**, 1212–1225 (2017). https://doi.org/10.1107/S1600576717007786

14 Plasser, F. TheoDORE: A toolbox for a detailed and automated analysis of electronic excited state computations. *J. Chem. Phys.* **152** (2020). https://doi.org/10.1063/1.5143076

15 Plasser, F., Bäppler, S. A., Wormit, M. & Dreuw, A. New tools for the systematic analysis and visualization of electronic excitations. II. Applications. *J. Chem. Phys.* **141** (2014).

https://doi.org/10.1063/1.4885820

16 Plasser, F. & Dreuw, A. High-Level Ab Initio Computations of the Absorption Spectra of Organic Iridium Complexes. *J. Phys. Chem. A* **119**, 1023–1036 (2015). https://doi.org/10.1021/jp5122917

17 Plasser, F., Wormit, M. & Dreuw, A. New tools for the systematic analysis and visualization of electronic excitations. I. Formalism. *J. Chem. Phys.* **141** (2014). https://doi.org/10.1063/1.4885819

18 Prampolini, G. *et al.* Dynamical and Environmental Effects on the Optical Properties of an Heteroleptic Ru(II)–Polypyridine Complex: A Multilevel Approach Combining Accurate Ground and Excited State QM-Derived Force Fields, MD and TD-DFT. *J. Chem. Theory Comput.* **15**, 529–545 (2019). https://doi.org/10.1021/acs.jctc.8b01031

19 Segalina, A., Assfeld, X., Monari, A. & Pastore, M. Computational Modeling of Exciton Localization in Self-Assembled Perylene Helices: Effects of Thermal Motion and Aggregate Size. *J. Phys. Chem. C* **123**, 6427–6437 (2019). https://doi.org/10.1021/acs.jpcc.9b00494

20 Segalina, A. *et al.* Deciphering Charge Transfer and Hydrogen Bonding Characteristics from Liquid Water XAS Spectra. *J. Chem. Theory Comput.* **21**, 11679–11686 (2025). https://doi.org/10.1021/acs.jctc.5c01156
