# Supplementary material for: Structural basis of FatB-mediated iron uptake via tyrosine/histidine direct coordination accompanying long-distance domain reorganization
Source: Nat Commun. 2026 Apr 18;17:5413. doi: 10.1038/s41467-026-72127-y (PMC13279818; doi:10.1038/s41467-026-72127-y)
Supplement: Supplementary file 2 — Description of Additional Supplementary Files [file 41467_2026_72127_MOESM2_ESM.pdf]

Description of Additional Supplementary Files

File Name: Supplementary Data 1
Description: MD simulation parameter and coordinate files. MD simulation parameter and coordinate files associated with the simulations reported in this study.
