# Supplementary material for: Structural basis of FatB-mediated iron uptake via tyrosine/histidine direct coordination accompanying long-distance domain reorganization
Source: Nat Commun. 2026 Apr 18;17:5413. doi: 10.1038/s41467-026-72127-y (PMC13279818; doi:10.1038/s41467-026-72127-y)
Supplement: Supplementary file 4 — Reporting Summary [file 41467_2026_72127_MOESM4_ESM.pdf]

nature portfolio

Corresponding author(s): Hyotcherl Ihee

Last updated by author(s): Apr 3, 2026

Reporting Summary

Nature Portfolio wishes to improve the reproducibility of the work that we publish. This form provides structure for consistency and transparency in reporting. For further information on Nature Portfolio policies, see our Editorial Policies and the Editorial Policy Checklist.

Statistics

For all statistical analyses, confirm that the following items are present in the figure legend, table legend, main text, or Methods section.

n/a	Confirmed	
☐	☒	The exact sample size (*n*) for each experimental group/condition, given as a discrete number and unit of measurement
☐	☒	A statement on whether measurements were taken from distinct samples or whether the same sample was measured repeatedly
☒	☐	The statistical test(s) used AND whether they are one- or two-sided<br>*Only common tests should be described solely by name; describe more complex techniques in the Methods section.*
☒	☐	A description of all covariates tested
☒	☐	A description of any assumptions or corrections, such as tests of normality and adjustment for multiple comparisons
☐	☒	A full description of the statistical parameters including central tendency (e.g. means) or other basic estimates (e.g. regression coefficient) AND variation (e.g. standard deviation) or associated estimates of uncertainty (e.g. confidence intervals)
☒	☐	For null hypothesis testing, the test statistic (e.g. *F*, *t*, *r*) with confidence intervals, effect sizes, degrees of freedom and *P* value noted<br>*Give P values as exact values whenever suitable.*
☒	☐	For Bayesian analysis, information on the choice of priors and Markov chain Monte Carlo settings
☒	☐	For hierarchical and complex designs, identification of the appropriate level for tests and full reporting of outcomes
☒	☐	Estimates of effect sizes (e.g. Cohen's *d*, Pearson's *r*), indicating how they were calculated

Our web collection on statistics for biologists contains articles on many of the points above.

Software and code

Policy information about availability of computer code

Data collection	X-ray diffraction data were collected using beamline-provided "Mx Data Collector" software at the 11C beamline of PLS-II (pohang Accelerator Laboratory, Korea). X-ray scattering data were collected using beamline-provided "Eiger2 Detector Control" software at the 4C beamline of PLS-II (Pohang Accelerator Laboratory, Korea).
Data analysis	Diffraction data were indexed, integrated, and scaled using HKL 2000 (version 722) and HKL 3000 (version 722). Structures were determined by molecular replacement using Phaser MR (version 2.8.3) in the PHENIX suite (version 1.20.1-4487), followed by model rebuilding with Autobuild and refinement with phenix.refine. Manual model adjustment and ligand fitting were performed in Coot(version 0.9.8.95). SAXS data were analyzed using the ATSAS suite (version 3.0.5), PRIMUS (version 3.0.5), GNOM (version 5.0), DAMMIF (version r13837), DAMMIN (version r13837), and SUPCOMB (version r13837). The fluorescence data were analyzed by nonlinear regression using a one-site binding model implemented in DYNAFIT (version 4.11.111).

For manuscripts utilizing custom algorithms or software that are central to the research but not yet described in published literature, software must be made available to editors and reviewers. We strongly encourage code deposition in a community repository (e.g. GitHub). See the Nature Portfolio guidelines for submitting code & software for further information.

Data

Policy information about availability of data

All manuscripts must include a data availability statement. This statement should provide the following information, where applicable:

- Accession codes, unique identifiers, or web links for publicly available datasets
- A description of any restrictions on data availability
- For clinical datasets or third party data, please ensure that the statement adheres to our policy

All crystallographic coordinates and associated structure-factor data have been deposited into the Protein Data Bank (PDB) under accession codes 21ZD (apo-FatB), 21ZE (FePB-FatB), 21ZF (FePBv-FatB), and 21ZG (Fe(3,4-DHB)2-FatB). The SAXS data have been deposited in the SASBDB database under accession codes SASDYB7 (apo-FatB), SASDYC7 (PB-FatB), SASDYD7 (FePB-FatB), SASDYE7 (FePBv-FatB), SASDYF7 (Fe(3,4-DHB)2-FatB). Source Data are provided as a Source Data file. MD simulation parameter and coordinate files are provided as Supplementary Data 1.

Research involving human participants, their data, or biological material

Policy information about studies with human participants or human data. See also policy information about sex, gender (identity/presentation), and sexual orientation and race, ethnicity and racism.

Reporting on sex and gender	N/A
Reporting on race, ethnicity, or other socially relevant groupings	N/A
Population characteristics	N/A
Recruitment	N/A
Ethics oversight	N/A

Note that full information on the approval of the study protocol must also be provided in the manuscript.

Field-specific reporting

Please select the one below that is the best fit for your research. If you are not sure, read the appropriate sections before making your selection.

[x] Life sciences [ ] Behavioural & social sciences [ ] Ecological, evolutionary & environmental sciences

For a reference copy of the document with all sections, see nature.com/documents/nr-reporting-summary-flat.pdf

Life sciences study design

All studies must disclose on these points even when the disclosure is negative.

Sample size	Sample sizes were chosen based on standard practice for structural and biophysical characterization. Fluorescence quenching experiments were performed as three independent titrations for each condition (n = 3). For SAXS, data were collected under continuous flow conditions, and each condition was measured as a series of 50 consecutive frames to improve signal-to-noise and monitor radiation damage.
Data exclusions	No data were excluded from the analyses.
Replication	Key findings were supported by independent and technical replication. Fluorescence quenching experiments were repeated as three independent titrations (n = 3). SAXS measurements were collected under continuous flow conditions with 50 consecutive frames per condition to ensure reproducibility and to monitor radiation damage.
Randomization	Randomization was not applicable because the study did not involve allocation of subjects or samples into experimental groups.
Blinding	Blinding was not applicable because samples were not assigned to experimental groups and data were collected and analyzed using predefined procedures.

Reporting for specific materials, systems and methods

We require information from authors about some types of materials, experimental systems and methods used in many studies. Here, indicate whether each material, system or method listed is relevant to your study. If you are not sure if a list item applies to your research, read the appropriate section before selecting a response.

Materials & experimental systems

n/a	Involved in the study
☒	☐ Antibodies
☒	☐ Eukaryotic cell lines
☒	☐ Palaeontology and archaeology
☒	☐ Animals and other organisms
☒	☐ Clinical data
☒	☐ Dual use research of concern
☒	☐ Plants

Methods

n/a	Involved in the study
☒	☐ ChIP-seq
☒	☐ Flow cytometry
☒	☐ MRI-based neuroimaging

Plants

Seed stocks	N/A
Novel plant genotypes	N/A
Authentication	N/A
