# Supplementary material for: Structural basis of FatB-mediated iron uptake via tyrosine/histidine direct coordination accompanying long-distance domain reorganization
Source: Nat Commun. 2026 Apr 18;17:5413. doi: 10.1038/s41467-026-72127-y (PMC13279818; doi:10.1038/s41467-026-72127-y)
Supplement: Supplementary file 5 — Transparent Peer Review File [file 41467_2026_72127_MOESM5_ESM.pdf]

nature portfolio

Peer Review File

Structural basis of FatB-mediated iron uptake via tyrosine/histidine direct coordination accompanying long-distance domain reorganization

Corresponding Author: Professor Hyotcherl Ihee

This file contains all reviewer reports in order by version, followed by all author rebuttals in order by version.

Version 0:

Reviewer comments:

Reviewer #1

(Remarks to the Author)
In this communication, Lee et al. report an in-depth investigation of the siderophore-binding FatB protein, combining SAXS, CD and computational analysis.
This combination leads to new insights compared to previously characterized siderophores known in the literature, as the authors unravel the recognition of the FePBv siderophore with a huge 29 A reorganisation of the flexible loop (244-254).

The coordination of the Fe(III) is unambiguously assigned, first from the crystallographic structure and through site-directed mutagenesis of the Tyr317/His252 pair.
The authors also investigate the spectroscopic signature of FePB in solution vs. in the protein, and resort to TDDFT with a QM/QM' embedding to reproduce and interpretate the peak at 492 nm wit
h natural transition orbitals to evidence a combination upon octahedral distortion.
The LMCT band at 320 nm is also reproduced by the DFT calculations, taking advantage of the CAM-B3LYP range-separated hybrid performance for these rather complicated iron-containing systems.
All the findings are clearly illustrated with high-quality figures.

This Communication concisely report important new insights relying on a combination of state-of-the-art techniques and extensive comparison to the closest structural homologs. FatB single out by a strong reorganization that brings the histidine His252 close to the iron center. It is a remarkable example of iron-driven allostery.

In my opinion, this manuscript can be published in Nature Communications with broad interest in structural biology.

Reviewer #2

(Remarks to the Author)
The manuscript entitled “Structural basis of FatB-mediated iron uptake via tyrosine/histidine direct coordination accompanying long-distance domain reorganization” by Lee, Ihee and coworkers describes the siderophore-binding protein FatB from Bacillus cereus and its interactions with its siderophore petrobactin (PB) in various (ferric) forms. It combines several crystal structures with several biophysical techniques (SAXS, CD, MD) to provide a structural framework for siderophore recognition.
This manuscript presents an unusually deep and coherent integration of structural biology and biophysics to elucidate siderophore recognition in a Gram-positive system. Overall, the manuscript contains a huge amount of data, is clearly written and contains high-quality figures. The identification of a protein-derived Tyr/His coordination sphere represents a significant conceptual advance.

Major comments:

Introduction:
•The Introduction provides an excellent and comprehensive overview of siderophore-mediated iron uptake, particularly in Gram-positive bacteria. However, the core mechanistic question—how FatB structurally accommodates chemically labile

ferric ligands while preserving high-affinity uptake—emerges relatively late. Explicitly stating this question earlier would sharpen the narrative focus and help guide readers through the subsequent structural and mechanistic analyses.
•The discussion of Trojan-horse antibiotics and TonB-dependent transporters is informative, but the mechanistic differences between Gram-negative and Gram-positive siderophore recognition are somewhat implicit. A more explicit contrast—particularly highlighting how SBP-mediated recognition imposes distinct structural constraints and opportunities—would better justify why FatB represents a fundamentally different and underexplored target space.
•While the Introduction effectively motivates the study, it is lacking clearly articulating specific hypotheses.
•The description of FePB photodecarboxylation and the formation of FePBn is clear, but the broader mechanistic implications of this chemistry for protein recognition are not fully articulated at the outset. Briefly previewing why photochemical instability poses a unique challenge for siderophore-binding proteins would help contextualize the later emphasis on protein-derived coordination and water-mediated compensation.

Results:
•The large-scale recruitment of the His252-containing loop (~29–30Å displacement) is a striking and important finding. While this motion is well described, its magnitude and functional significance could be emphasized more consistently across the results, particularly when transitioning from static structures to MD simulations, to reinforce how unusual and consequential this rearrangement is among class-III SBPs.
•The manuscript references CeuE, FeuA, FhuD, and HutB at various points, but these comparisons are somewhat dispersed. A more systematic comparison—perhaps summarizing how FatB differs in coordination strategy, loop mobility, or ligand tolerance relative to these systems—would further highlight the novelty of the observed Tyr/His-mediated coordination mode.
•I have briefly checked the maps that the authors made available for review. Overall, they are of excellent quality. However, many Asp/Glu decarboxylations can be seen in the FePBv-FatB structure/map. According to the methods section, the preparation of the photoproduct has been done before setting up xtals, so this decarboxylation in the maps cannot be explained by photoproduct preparation. Could the authors please comment on this? Have they checked for radiation damage?

Minor comments:
•Please display Suppl. Fig. S1 in same orientation as Fig. 1c to facilitate comparison
•Several supplementary figures have not been referenced in manuscript. Please either mention them in text or remove then.
•Could the oxidative decarboxylation of the PBn be shown with electron density map in some figure?
•All Greek characters are absent from figure legends. It should be alpha, beta, nu and chi instead of a,b,n,c
•SAXS data analysis and interpretation is good. However, SAXS data should be submitted to SASBDB.
•Many statistical values in the crystallization and SAXS tables are given with unusual/unrealistic accuracy. Please round them to useful digits. For example: Dmax: 73.46 A or RMSD: 1.816., these don't make sense.
•Line 239: could the hydrogen bond be shown in some figure?
•I am not convinced whether the quantum chemical analysis adds much to the manuscript. In my opinion, this could be shortened or moved to SI.
•Table 2: please explain the Kd differences between FeBP and FeBPn (photoproduct). Why does the latter bind 4x tighter to FatB?

Reviewer #3

(Remarks to the Author)
In this manuscript, Lee et al. investigate the structural and mechanistic basis of ferric siderophore recognition by the Gram-positive substrate-binding protein FatB from Bacillus cereus. Using X-ray crystallography, SAXS, CD spectroscopy, mutagenesis, fluorescence binding assays, molecular dynamics simulations, and TD-DFT calculations, the authors characterize FatB in its apo form and in complex with ferric petrobactin (FePB), its ferric photoproduct, and a catecholate mimic.

The structures reveal that ferric ligand binding induces a conserved hinge-like domain closure characteristic of class-III SBPs. A central finding is that FatB actively remodels the Fe(III) coordination sphere: a protein-derived Tyr317/His252 pair directly coordinates the metal, replacing native ligand donors. In the photoproduct complex, a structured water network compensates for photodecarboxylation-induced ligand changes, preserving high-affinity binding. Mutational analysis supports ligand-dependent contributions of Tyr317 and His252, and spectroscopic/quantum-chemical analyses indicate that Tyr317 modulates the excited-state electronic structure of the bound ferric complex.

Overall, the study is thorough, and the authors support their claims with multiple experimental and computational techniques. However, the following issues should be addressed before the manuscript can be accepted for publication:

1. In lines 129-132 from the main text, the authors describe key conformational changes based on the superposition of apo-FatB and FePB-FatB. It is unclear how the uncertainty in the provided values was determined. Additionally, the value reported (8.2) in "a straightening of the central alpha-helix by 8.2 +/- 0.2 …" differs from the value (8.3) depicted in Figure 2E.

2. The authors clearly define 'apo-FatB' as the ligand-free form. It would be helpful for a more general readership if the authors specified that 'holo' referred to the ligand-bound form (line 136; main text).

3. The authors state a Guinier analysis was performed on the SAXS data in lines 160-162 of the main text. It would be

helpful to include the Guinier plots in the supplementary information, as they are important for assessing quality of SAXS data.

4. In figure 3, it would be helpful if the authors provided a difference in the experimental SAXS profiles for the apo and ligand bound FatB. This is not essential to include in the main text, but it would be beneficial for the reader to at least include in the supplementary information.

5. In the figure 3 caption (line 950), it should read "χ2" instead of "c2"

6. Finally, for each state depicted in Figure 3, the authors present the corresponding DAMFILT bead model superimposed on the structure. It would be helpful for the authors to provide a justification for using the DAMFILT model, as this "averaged" model does not necessarily ensure that the theoretical SAXS profile calculated from the DAMFILT envelope matches the experimental SAXS data.
In ab initio SAXS reconstructions, two commonly reported models that directly fit the experimental data are the most-probable model (e.g., selected based on normalized spatial discrepancy from independent DAMMIF runs) and the DAMMIN-refined model. It would be more appropriate to compare these models with the crystal structures.

Reviewer #4

(Remarks to the Author)
I co-reviewed this manuscript with one of the reviewers who provided the listed reports. This is part of the Nature Communications initiative to facilitate training in peer review and to provide appropriate recognition for Early Career Researchers who co-review manuscripts.

Version 1:

Reviewer comments:

Reviewer #2

(Remarks to the Author)
The authors have addressed all my suggestions and concerns in their rebuttal letter. I particularly appreciate their efforts to acquire a lower-dose FePBv-FatB diffraction dataset. I have no further comments and support publication.

Reviewer #3

(Remarks to the Author)
The authors have addressed all of my questions. I recommend the manuscript for publication in its current form.

Reviewer #4

(Remarks to the Author)
I co-reviewed this manuscript with one of the reviewers who provided the listed reports. This is part of the Nature Communications initiative to facilitate training in peer review and to provide appropriate recognition for Early Career Researchers who co-review manuscripts.

Open Access This Peer Review File is licensed under a Creative Commons Attribution 4.0 International License, which permits use, sharing, adaptation, distribution and reproduction in any medium or format, as long as you give appropriate credit to the original author(s) and the source, provide a link to the Creative Commons license, and indicate if changes were made.
In cases where reviewers are anonymous, credit should be given to 'Anonymous Referee' and the source.
The images or other third party material in this Peer Review File are included in the article's Creative Commons license, unless indicated otherwise in a credit line to the material. If material is not included in the article's Creative Commons license and your intended use is not permitted by statutory regulation or exceeds the permitted use, you will need to obtain permission directly from the copyright holder.
To view a copy of this license, visit https://creativecommons.org/licenses/by/4.0/

Responses to the comments from Reviewer #1

In this communication, Lee et al. report an in-depth investigation of the siderophore-binding FatB protein, combining SAXS, CD and computational analysis.
This combination leads to new insights compared to previously characterized siderophores known in the literature, as the authors unravel the recognition of the FePBv siderophore with a huge 29 A reorganisation of the flexible loop (244-254).

The coordination of the Fe(III) is unambiguously assigned, first from the crystallographic structure and through site-directed mutagenesis of the Tyr317/His252 pair.
The authors also investigate the spectroscopic signature of FePB in solution vs. in the protein, and resort to TDDFT with a QM/QM' embedding to reproduce and interpretate the peak at 492 nm wit
h natural transition orbitals to evidence a combination upon octahedral distortion.
The LMCT band at 320 nm is also reproduced by the DFT calculations, taking advantage of the CAM-B3LYP range-separated hybrid performance for these rather complicated iron-containing systems.
All the findings are clearly illustrated with high-quality figures.

This Communication concisely report important new insights relying on a combination of state-of-the-art techniques and extensive comparison to the closest structural homologs. FatB single out by a strong reorganization that brings the histidine His252 close to the iron center. It is a remarkable example of iron-driven allostery.

In my opinion, this manuscript can be published in Nature Communications with broad interest in structural biology.
→ We sincerely appreciate the reviewer's supportive and insightful comments on our manuscript. We are pleased that the reviewer recognized the value of our integrated approach, combining experimental techniques with high-level computational analysis to elucidate the iron-uptake mechanism of FatB. As the reviewer correctly pointed out, the ~29 Å reorganization of the flexible loop and the subsequent Tyr317/His252-mediated Fe(III) coordination are central to our findings.

Responses to the comments from Reviewer #2

The manuscript entitled "Structural basis of FatB-mediated iron uptake via tyrosine/histidine direct coordination accompanying long-distance domain reorganization" by Lee, Ihee and coworkers describes the siderophore-binding protein FatB from Bacillus cereus and its interactions with its siderophore petrobactin (PB) in various (ferric) forms. It combines several crystal structures with several biophysical techniques (SAXS, CD, MD) to provide a structural framework for siderophore recognition.

This manuscript presents an unusually deep and coherent integration of structural biology and biophysics to elucidate siderophore recognition in a Gram-positive system. Overall, the manuscript contains a huge amount of data, is clearly written and contains high-quality figures. The identification of a protein-derived Tyr/His coordination sphere represents a significant conceptual advance.

→ We sincerely thank the reviewer for recognizing the importance of our work and for supporting its publication. We also greatly appreciate the reviewer's insightful comments, which we address point-by-point below.

Major comments:

Introduction:

The Introduction provides an excellent and comprehensive overview of siderophore-mediated iron uptake, particularly in Gram-positive bacteria. However, the core mechanistic question—how FatB structurally accommodates chemically labile ferric ligands while preserving high-affinity uptake—emerges relatively late. Explicitly stating this question earlier would sharpen the narrative focus and help guide readers through the subsequent structural and mechanistic analyses.

The discussion of Trojan-horse antibiotics and TonB-dependent transporters is informative, but the mechanistic differences between Gram-negative and Gram-positive siderophore recognition are somewhat implicit. A more explicit contrast—particularly highlighting how SBP-mediated recognition imposes distinct structural constraints and opportunities—would better justify why FatB represents a fundamentally different and underexplored target space.

→ We appreciate the reviewer's insightful suggestions. To sharpen the narrative focus earlier and to make the mechanistic contrast explicit, we revised the Trojan-horse paragraph to directly compare Gram-negative TonB-dependent uptake with Gram-positive SBP-mediated recognition. We further added a bridging sentence that introduces FatB and states the core mechanistic question earlier, so that readers are guided into the subsequent structural and mechanistic analysis.

The relevant parts of the manuscript were revised as follows.

Original Text:

"Such strategies successfully target Gram-negative pathogens, as exemplified by cefiderocol, a siderophore-cephalosporin conjugate that exploits TonB-dependent transporters for cell entry[32-34]. However, corresponding mechanisms in Gram-positive bacteria are sparsely characterized due to scarce structural information.

Among class-III SBPs, FatB serves as the cognate SBP for petrobactin (PB), a catecholate-type siderophore widely used by *Bacillus* species[35,36]."

Revised Text:

"Such strategies successfully target Gram-negative pathogens, as exemplified by cefiderocol, a siderophore-cephalosporin conjugate that exploits TonB-dependent transporters for cell entry[32-34]. Mechanistically, whereas Gram-negative uptake is initiated by TonB-dependent outer-membrane

receptors that select and translocate ferric complexes through a dedicated channel, Gram-positive uptake relies on SBP-mediated capture as the primary extracellular selectivity step followed by handoff to an ABC importer. This SBP-centered architecture imposes distinct constraints and opportunities for stabilizing ferric coordination during recognition and delivery.

In this context, FatB provides an opportunity to address a central mechanistic question: how can an SBP preserve high-affinity uptake while accommodating ferric ligands that are chemically labile and can undergo structural modification? FatB is the cognate class-III SBP for petrobactin (PB), a catecholate-type siderophore widely used by *Bacillus* species[35,36].

While the Introduction effectively motivates the study, it is lacking clearly articulating specific hypotheses.

→ We agree with the reviewer that explicitly stating testable hypotheses would strengthen the framing of the study. We therefore added a concise hypothesis statement immediately before the final overview paragraph of the Introduction section, so that the subsequent structural and mechanistic analyses are guided by clearly defined, testable expectations.

The relevant parts of the manuscript were revised as follows.

Original Text:

"[...] against Gram-positive pathogens[9,15,40].

To address these gaps, we investigated the FatB [...]"

Revised Text:

"[...] against Gram-positive pathogens[9,15,40].

We hypothesized that FatB maintains high-affinity uptake across FePB and its photoproduct, $FePB^{\nu}$, by coupling ligand-dependent conformational gating with protein- and solvent-mediated stabilization within the binding cleft. In this view, chemical modification of the ferric ligand may be accommodated by compensatory interactions that preserve a transport-compatible complex. To test this hypothesis, we investigated FatB [...]"

Results:

The description of FePB photodecarboxylation and the formation of FePBn is clear, but the broader mechanistic implications of this chemistry for protein recognition are not fully articulated at the outset. Briefly previewing why photochemical instability poses a unique challenge for siderophore-binding proteins would help contextualize the later emphasis on protein-derived coordination and water-mediated compensation.

→ We thank the reviewer for this insightful suggestion. To better articulate the mechanistic implications of FePB photochemistry for protein recognition at the outset of the Results section, we added a brief preview explaining why photochemical modification of ferric siderophores can challenge SBP-mediated uptake by perturbing ferric coordination and receptor complementarity. This addition provides context for the subsequent structural analysis of protein-derived coordination and second-sphere stabilization mechanisms discussed later.

Original Text:

"Previous biochemical work has shown that apo-FatB binds its native ferric siderophore, FePB, the photolyzed derivative $FePB^{\nu}$, and a minimal catecholate mimic $Fe(3,4\text{-DHB})_2$ (Fig. 1d)[36]. However, the structural basis by which FatB accommodates [...]"

Revised Text:

"Photochemical modification of ferric siderophores can pose a challenge for SBP-mediated uptake because changes in the ligand scaffold may perturb the ferric coordination environment and the interaction features required for stable receptor capture and delivery[10]. This challenge motivated us to

investigate FatB, for which previous biochemical work indicated that high-affinity binding was maintained even after photodecarboxylation of the ferric ligand. Specifically, apo-FatB binds its native ferric siderophore, FePB, the photolyzed derivative $FePB^{v}$, and a minimal catecholate mimic $Fe(3,4\text{-}DHB)_2$ (Fig. 1d)[36]. However, the structural basis by which FatB accommodates [...]"

The large-scale recruitment of the His252-containing loop (~29–30Å displacement) is a striking and important finding. While this motion is well described, its magnitude and functional significance could be emphasized more consistently across the results, particularly when transitioning from static structures to MD simulations, to reinforce how unusual and consequential this rearrangement is among class-III SBPs.

→ We thank the reviewer for highlighting the importance of the His252-loop recruitment. To emphasize both the magnitude (~29–30 Å) and the functional significance of this rearrangement more consistently, particularly at the transition from the static structures to the MD simulations, we added a bridging sentence that explicitly frames the MD analysis as a test of whether apo-FatB samples loop-proximal conformations needed to assemble the Tyr/His coordination site. We also revised the opening sentence of the second paragraph in "His252 loop dynamics~" section to reiterate that apo-FatB transiently explores loop-proximal conformations that move His252 toward the ferric-ligand-bound site geometry.

Original Text:

"[...] the His252-containing loop into the binding cleft. To understand how His252 dynamically accesses the binding geometry, we performed molecular dynamics simulations of apo-FatB.

Simulations reveal that apo-FatB is not a rigid scaffold. The His252-containing loop (residues 244–254) exhibits the highest [...]"

Revised Text:

"[...] the His252-containing loop into the binding cleft. This displacement underscores that high-affinity ferric capture would require substantial rearrangement of the His252-containing loop, raising the question of whether apo-FatB spontaneously samples loop-proximal conformations that could facilitate assembly of the Tyr/His site upon ligand encounter. To understand how His252 dynamically accesses the binding geometry, we performed molecular dynamics (MD) simulations of apo-FatB.

MD simulations indicate that apo-FatB is not a rigid scaffold and can transiently explore loop-proximal conformations that move His252 toward the ferric-ligand-bound site geometry. In particular, residues 244–254, which comprise the His252-containing loop, exhibit the highest [...]"

The manuscript references CeuE, FeuA, FhuD, and HutB at various points, but these comparisons are somewhat dispersed. A more systematic comparison—perhaps summarizing how FatB differs in coordination strategy, loop mobility, or ligand tolerance relative to these systems—would further highlight the novelty of the observed Tyr/His-mediated coordination mode.

→ We thank the reviewer for this suggestion. We agree that the comparisons to *Cj*CeuE, *Bs*FeuA, *Vc*FhuD, and *Vc*HutB are introduced at different points in the Results because each system is cited to benchmark a distinct mechanistic aspect, which can make the overall contrast appear dispersed. To address the point raised by the reviewer, we added an integrative paragraph in the Discussion that organizes these cross-system comparisons into three systematic benchmarks: (i) Fe(III) coordination strategy (*Cj*CeuE versus FatB's Tyr/His-defined first-sphere donors), (ii) conformational gating quantified by the range of C-terminal domain rotations across class-III SBPs (including *Bs*FeuA and *Vc*FhuD, with FatB in an intermediate regime), and (iii) flexibility and loop mobility rationalized by hinge architecture and packing around the central α-helix (*Vc*FhuD versus *Vc*HutB, and the highly dynamic His252-containing loop in apo-FatB). This addition clarifies how FatB differs from

representative class-III SBPs in coordination, gating, and mobility, and thereby more directly highlights the novelty of the Tyr/His-mediated coordination mode.

Original Text:

"[...] indicating how FatB stabilizes ferric siderophores while maintaining high-affinity recognition across chemically distinct ligands.

Beyond structural investigation, [...]"

Revised Text:

"[...] indicating how FatB stabilizes ferric siderophores while maintaining high-affinity recognition across chemically distinct ligands.

The cross-system comparisons with *Cj*CeuE, *Bs*FeuA, *Vc*FhuD and *Vc*HutB given in the Results section can be organized into the following three characteristics that make FatB distinctive among class-III SBPs. First, at the level of Fe(III) coordination, while *Cj*CeuE provides a clear reference for protein participation in completing octahedral geometry for tetradentate ligands[22,53], in FatB, a Tyr/His pair provides the decisive first-sphere donors even for ferric petrobactin species that are intrinsically hexadentate in solution. Second, FatB undergoes ligand-dependent conformational gating via hinge-driven domain closure, with a C-terminal domain rotation of ~15° placing FatB between the larger global motions reported for *Bs*FeuA and the smaller motions reported for *Vc*FhuD[21]. This comparison emphasizes that FatB lies in a moderate regime of global domain closure while ferric capture is accompanied by an unusually pronounced local rearrangement of the His252-containing loop. Third, FatB exhibits pronounced loop mobility centered on the His252-containing loop, which undergoes long-range recruitment upon ferric binding and constitutes the most dynamic region in apo-FatB by MD simulation. A previous comparison of *Vc*FhuD and *Vc*HutB suggested that looser packing around the central α-helix is associated with greater interdomain mobility and enhanced loop plasticity[26]. Consistent with this framework, apo-FatB shows a similarly less tightly packed environment around the central helix, including a one-turn-shorter central α-helix and a less parallel arrangement of helices α4 and α11, providing a plausible structural basis for the unusually large recruitment of His252 into the coordination sphere. Taken together, these comparisons suggest that FatB combines a permissive hinge architecture with unusually large His252-loop recruitment, providing a structural context for protein-derived Tyr/His coordination.

Beyond structural investigation, [...]"

I have briefly checked the maps that the authors made available for review. Overall, they are of excellent quality. However, many Asp/Glu decarboxylations can be seen in the FePBv-FatB structure/map. According to the methods section, the preparation of the photoproduct has been done before setting up xtals, so this decarboxylation in the maps cannot be explained by photoproduct preparation. Could the authors please comment on this? Have they checked for radiation damage?

→ We thank the reviewer for this important observation. We agree that the Asp/Glu carboxylate-centered negative difference density in the original $FePB^{v}$-FatB map cannot be attributed to the photoproduct preparation step and is more appropriately evaluated as a potential X-ray radiation damage artifact.

To address this concern, we collected and analyzed an additional $FePB^{v}$-FatB diffraction dataset using crystals from the same batch, but with a substantially reduced X-ray exposure (20% of the original exposure time) and a shorter sample-to-detector distance, which also provided higher-resolution diffraction data. We then reprocessed and refined the structure and re-examined the corresponding electron-density maps.

In the lower-dose dataset, the number and intensity of Asp/Glu carboxylate-associated negative difference peaks were substantially reduced, supporting the reviewer's concern that a significant fraction of the previously observed carboxylate-centered negative density likely reflected X-ray radiation damage in the original dataset. We have therefore revised the FePB^v-FatB structural analysis using the updated dataset. We have also updated the corresponding wwPDB deposition by replacing the FePB^v-FatB coordinates/structure factors with those refined from the lower-dose dataset while retaining the same entry under hold-for-publication status (PDB ID: 21ZF).

Importantly, the updated structure preserves the main conclusion that FatB stabilizes the FePB^v-bound state through a structured solvent relay at the remodeled ligand interface. At the same time, the higher-quality dataset allowed us to refine the details of this relay network: in the revised model, the interfacial solvent relay is described as four ordered water molecules (W339, W62, W171, and W147), and the previously assigned water (W159) is not resolved in the updated map; instead, the local density in that region is accounted for by the repositioned enolic hydroxyl oxygen of PB^v. We appreciate the reviewer's comment, which led us to perform this additional validation and improve the quality and accuracy of the FePB^v-FatB structural analysis. The revised relay topology is now described in the Results section and reflected in the updated figures, including Fig. 2b, 3a, 4b, 4e, Supplementary Fig. 5 (formerly Supplementary Fig. 4), Supplementary Fig. 11 (formerly Supplementary Fig. 8), Supplementary Fig. 16 (formerly Supplementary Fig. 12), and Supplementary Fig. 21 (formerly Supplementary Fig. 16) as well as Supplementary Table 2–5). In addition, new Supplementary Figs. 9, 15, and 17 have been added on the basis of the newly collected lower-dose FePB^v-FatB dataset to document the revised structure and the improved map quality.

Original Text:

"[...] The FePB^v-FatB structure reveals that FatB compensates for this loss by organizing three ordered water molecules (W159, W13, and W104) into a structured relay that fills the physical void and replaces the missing interactions (Fig. 4b and Supplementary Fig. 12). Crystallographic data unequivocally support the stability of the relay; the water molecules are well-defined with full occupancy, and exhibit B-factors consistent with their environments. The two buried waters (W13 and W104) show low B-factors (23.5 Å^2 and 28.7 Å^2), comparable to neighboring protein atoms, confirming their stable, anchoring nature. The solvent-exposed W159 displays a higher B-factor (43.3 Å^2), reflecting its dynamic role.

This templated solvent network is crucial for stabilizing the modified ligand (Fig. 4b). By forming an extended hydrogen-bonding network, the relay restores interfacial connectivity between the siderophore and the protein. The network initiates at the solvent interface with W159, which anchors the assembly by hydrogen-bonding to the amide and secondary amine nitrogen atoms on the PB^v backbone. The pathway continues through W13, bridging a catecholate oxygen of the ligand to the Arg109 guanidinium group, and is completed by the innermost W104, which connects the second catecholate oxygen to an amide nitrogen on the PB^v backbone (Fig. 4b). This solvent-templated relay functionally replaces the missing donor group, thereby stabilizing the decarboxylated ligand and preserving the octahedral geometry of the Fe(III) center.

To probe the binding plasticity [...]"

Revised Text:

"[...] The FePB^v-FatB structure reveals that FatB compensates for this loss by organizing four ordered water molecules (W339, W62, W171, and W147) into a structured relay that fills the physical void and replaces the missing interactions (Fig. 4b and Supplementary Fig. 16). Crystallographic data support the stability of the relay; the water molecules are well-defined with full occupancy and display relatively

low B-factors (24.6, 21.1, 27.0, and 29.2 Å^2), comparable to neighboring protein atoms, indicating a well-ordered interfacial solvent relay.

This templated solvent network is crucial for stabilizing the modified ligand (Fig. 4b). By forming an extended hydrogen-bonding network, the relay restores interfacial connectivity between the siderophore and the protein. The network initiates at the solvent-facing side of the cavity with W339, which couples a catecholate oxygen to the backbone-side enolic hydroxyl oxygen of PB^v and thereby stabilizes the backbone-side portion of the ligand within the remodeled pocket. In addition, W339 bridges these ligand oxygen atoms to the Arg109 guanidinium group, sustaining protein-ligand coupling at the interface. The relay is propagated by W62, which engages the second catecholate oxygen and transmits the solvent-mediated linkage into the interior of the network. This connectivity is further relayed through W171 to the terminal water W147, which hydrogen-bonds with the carboxylate oxygen on the catecholate-bearing arm of PB^v. Together, these waters form a continuous solvent-mediated hydrogen-bonding network that stabilizes the decarboxylated ligand across the remodeled FePB^v-binding interface (Fig. 4b). This solvent-templated relay functionally compensates for the lost interactions, while maintaining the octahedral geometry of the Fe(III) center.

To probe the binding plasticity [...]"

Minor comments:

Please display Suppl. Fig. S1 in same orientation as Fig. 1c to facilitate comparison.

→ We thank the reviewer for this helpful suggestion. To facilitate direct visual comparison, we have re-rendered Supplementary Fig. 1 using the same viewing orientation as Fig. 1c.

Several supplementary figures have not been referenced in manuscript. Please either mention them in text or remove then.

→ We thank the reviewer for identifying this oversight. We have ensured that every supplementary item is referenced in the manuscript, at least in the Supplementary Information if not in the main text itself. In particular, we now refer to Supplementary Table 1 in the opening paragraph of the Results section when introducing the recombinant FatB construct and sample preparation. We also found that the original order of Supplementary Figures 4 and 5 did not fully align with the sequence in which they are cited in the text. We therefore swapped their order to improve clarity and consistency.

Original Text:

"To establish a structural basis for understanding the function of FatB, we first determined the X-ray crystal structure of FatB in its ligand-free (hereafter referred to as apo-FatB) form."

Revised Text:

"To establish a structural basis for understanding the function of FatB, recombinant FatB was expressed in *E. coli* and purified as described in the Methods section (Supplementary Table 1). We then determined the X-ray crystal structure of FatB in its ligand-free (hereafter referred to as apo-FatB) form."

Could the oxidative decarboxylation of the PBn be shown with electron density map in some figure?

→ We thank the reviewer for this suggestion. Although electron density maps cannot directly visualize the oxidative chemistry step itself, they can provide structural evidence that the bound ligand lacks the carboxylate group expected for FePB and is therefore consistent with the decarboxylated photoproduct (FePB^v). To address this point, we added a new electron-density comparison figure (Supplementary Fig. 15) showing the ligand region in the FePB-FatB and FePB^v-FatB complexes in the same orientation and at the same map contour level ($2mF_o$-DF_c). In the FePB-FatB complex, the density is continuous through the carboxylate group, consistent with the intact ligand model. In contrast, in the FePB^v-FatB

complex, the density expected for the carboxylate group is not observed at the corresponding position, and the nearby electron density assigned as W62 is spatially separated from the ligand. Together, these maps support our assignment that photodecarboxylation has occurred and the bound ligand in the FePB^{ν}-FatB structure lacks the carboxylate group.

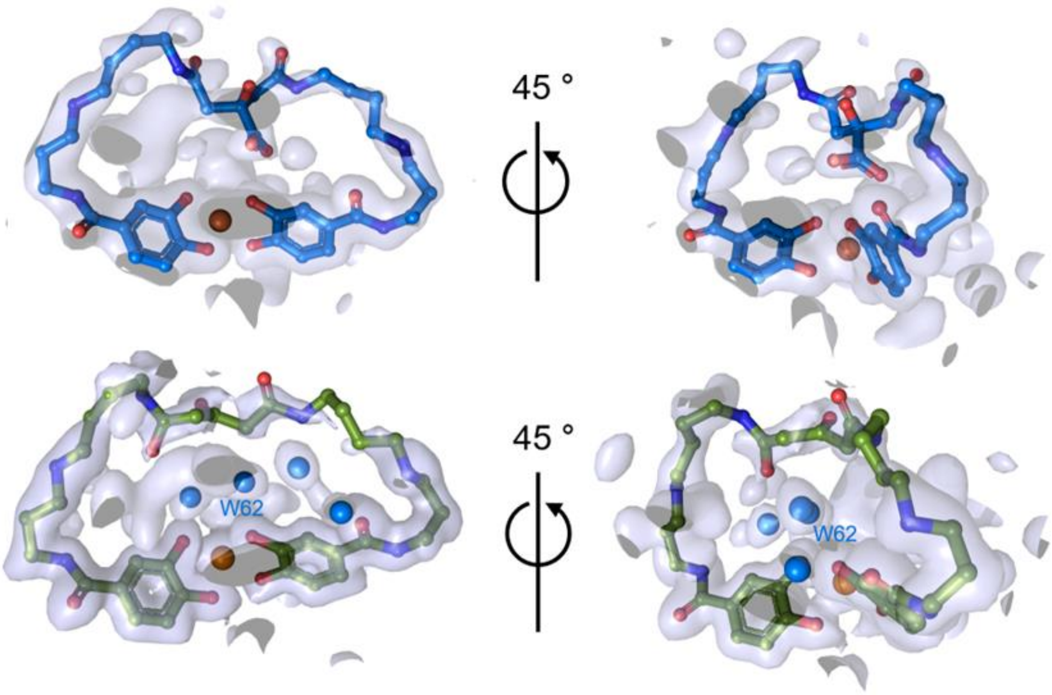

Supplementary Fig. 15. Electron-density comparison of FePB and FePB^{ν} in the FatB binding site. Representative $2mF_o–DF_c$ electron-density maps (gray semitransparent surfaces) are shown around the ferric ligand bound in the FePB-FatB (top, PB; blue sticks) and FePB^{ν}-FatB (bottom, PB^{ν}; green sticks) complexes. For each complex, the ligand region is displayed in two orthogonal views (right panels rotated 45° relative to the left). In the FePB-FatB structure, the density follows the carboxylate-bearing arm of FePB, consistent with the intact ligand model. In the FePB^{ν}-FatB structure, the density expected for the corresponding carboxylate group is absent, consistent with assignment of the decarboxylated photoproduct. Notably, the ordered water W62, which occupies the cavity adjacent to the ligand in the FePB^{ν}-binding interface, is spatially separated from the ligand density. To allow comparison of local density under identical visualization conditions, all maps in this figure are contoured at 0.75σ. The Fe(III) ion is shown as an orange sphere, and ordered solvent molecules associated with the FePB^{ν}-binding interface are shown as blue spheres.

All Greek characters are absent from figure legends. It should be alpha, beta, nu and chi instead of a,b,n,c

→ We appreciate the reviewer for pointing this out. We corrected the affected figure legends by ensuring that Greek symbols are rendered explicitly and consistently (α, β, ν, χ; alpha, beta, nu, chi) throughout the manuscript and Supplementary Information, and we re-checked all figure legends for consistent display.

SAXS data analysis and interpretation is good. However, SAXS data should be submitted to SASBDB.

→ We thank the reviewer for the positive evaluation of our SAXS data analysis. Following the reviewer's recommendation, we have submitted the experimental SAXS profiles to SASBDB. The data are now available under the following accession codes:

SASDYB7: apo-FatB, SASDYC7: PB-FatB, SASDYD7: FePB-FatB, SASDYE7: FePB^{v}-FatB, and SASDYF7: Fe(3,4-DHB)$_2$-FatB.

Many statistical values in the crystallization and SAXS tables are given with unusual/unrealistic accuracy. Please round them to useful digits. For example: Dmax: 73.46 A or RMSD: 1.816., these don't make sense.

→ We thank the reviewer for identifying this oversight. The numbers have been revised to report the appropriate number of significant figures, consistent with the magnitude of the associated errors.

Line 239: could the hydrogen bond be shown in some figure?

→ We thank the reviewer for this helpful suggestion. To address this point more clearly, we have added a new Supplementary Figure (Supplementary Fig. 17) that explicitly shows the hydrogen-bonding networks in the binding clefts of FePB-FatB, FePB^{v}-FatB, and Fe(3,4-DHB)$_2$-FatB (panels a–c). In this figure, hydrogen bonds are directly indicated by dashed lines, including the water-mediated hydrogen-bonding network in the FePB^{v}-FatB complex.

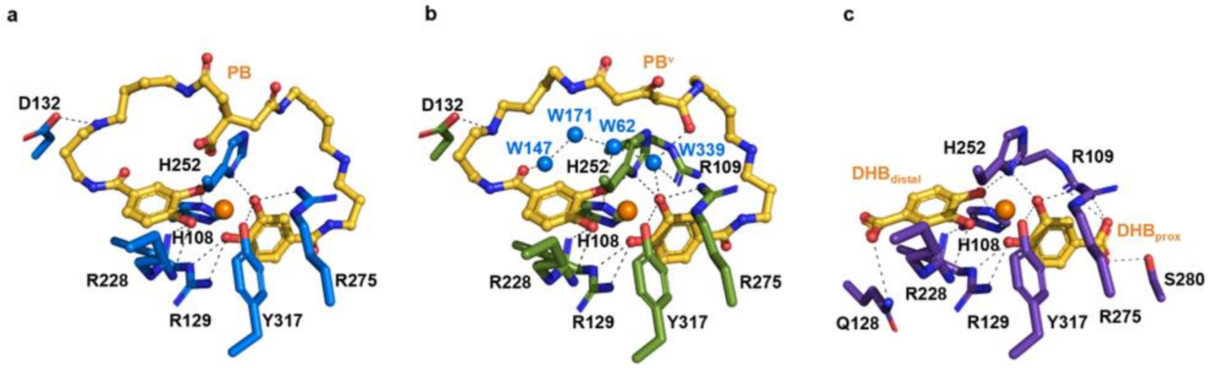

Supplementary Fig. 17. Hydrogen-bonding networks in the FatB binding clefts. Detailed views of the binding clefts of (a) FePB-FatB, (b) FePB^{v}-FatB, and (c) Fe(3,4-DHB)$_2$-FatB showing the hydrogen-bonding interactions around the ligands. Hydrogen bonds are indicated by black dashed lines. Ligands are shown as yellow sticks, and Fe(III) ions as orange spheres. Protein residues are shown as sticks and colored blue, green, and purple in panels a, b, and c, respectively. Ordered water molecules in the FePB^{v}-FatB complex are shown as blue spheres and labeled (W147, W171, W62, and W339).

I am not convinced whether the quantum chemical analysis adds much to the manuscript. In my opinion, this could be shortened or moved to SI.

→ We thank the reviewer for this helpful comment and agree that the presentation of the quantum chemical analysis in the original manuscript was more detailed than necessary for the main text. In response, we have substantially streamlined this part of the manuscript and moved the more technical aspects of the computational analysis, together with the more detailed discussion, to the Supplementary Information. In the revised main text, we now retain only the minimal computational results needed to support the experimental spectroscopy and to convey the key mechanistic message in a form that is accessible to the broad readership of *Nature Communications*. We also shortened the corresponding Methods section so that it now contains only the essential computational setup, while referring the

reader to a new Supplementary Note 1 for the detailed description of the excited-state analysis, including the use of natural transition orbitals (NTOs) and one-particle transition-density matrix (1TDM) analysis. In Supplementary Note 1, we provide the theoretical rationale, fragment-based decomposition scheme, and the expanded interpretation of the calculated spectra for free FePB and the FePB-FatB complex. We believe this revision has improved the balance between mechanistic rigor and readability, while ensuring that the central experimental conclusions remain properly supported.

The relevant revisions to the Results section are as follows:

Original Text:

"[...] by our time-dependent density functional theory (TD–DFT) calculations[55]. To clarify the origin of these bands, we decomposed the one-particle transition-density matrix (1TDM) into fragment contributions, enabling quantitative assessment of all major excitation pathways: LMCT, metal-to-ligand charge transfer (MLCT), ligand-to-ligand charge transfer (LLCT), ligand-centered (LC), and metal-centered (MC)."

Revised Text:

"[...] by our time-dependent density functional theory (TD–DFT) calculations[55]. To clarify the origin of these bands, we analyzed the charge-transfer character of the excited states,[57-60] a fragment-based excited-state analysis widely used to characterize charge-transfer states in complex molecular and condensed-phase systems.[61-63]."

Original Text:

"[...] a heterogeneous mixture of LMCT and LC contributions, with MLCT and MC contributions remaining negligible across the full spectrum (Fig. 5b, c)[38,57,58]."

Revised Text:

"[...] a heterogenous mixture of LMCT and ligand-centered (LC) contributions (Fig. 5b, c)[38,64,65]."

Original Text:

"[...] enabling a direct comparison between experiment and theory. 1TDM decomposition demonstrates that the red shift does not arise from a change in the fundamental nature of the transition; in the FePB-FatB model, the lowest-energy LMCT state remains predominantly catecholate→Fe(III) in character, with negligible Tyr→Fe contribution in the corresponding natural transition orbitals (NTOs)[63-67]."

Revised Text:

"[...] enabling a direct comparison between experiment and theory. Analysis of the excited-state character shows that the red shift does not arise from a change in the fundamental nature of the transition; in the FePB-FatB model, the lowest-energy LMCT state remains predominantly catecholate→Fe(III) in character, with negligible Tyr→Fe contribution[57-61]."

Original Text:

"[...] ligand-to-ligand charge-transfer (LLCT) character. Notably, this redistribution occurs within the ~300–320 nm window where the FePB photoreaction is generally induced, providing a quantitative description of how the protein environment populates a modified ensemble of excited states in this energy range."

Revised Text:

"[...] ligand-to-ligand charge-transfer (LLCT) character. Notably, this redistribution occurs within the ~300–320 nm window where the FePB photoreaction is generally induced."

Revisions to the Methods and Supplementary Information:

The Methods section was condensed to retain only the essential computational setup, while the detailed description of the excited-state analysis was moved to a newly added Supplementary Note 1. Specifically, the original detailed discussion of full TD–DFT versus TDA, TheoDORE-based 1TDM decomposition, fragment partitioning, and oscillator-strength-weighted spectral analysis was removed from the main Methods section and is now presented in Supplementary Note 1 at an appropriate level of detail. The revised main Methods section now includes the following sentence:

"A detailed description of the excited-state analysis, including the use of natural transition orbitals (NTOs) and the one-particle transition-density matrix (1TDM), is provided in Supplementary Note 1."

Table 2: please explain the Kd differences between FeBP and FeBPn (photoproduct). Why does the latter bind 4x tighter to FatB?

→ We thank the reviewer for this important question. Our data show that FePB^{v} binds to FatB with ~4-fold higher apparent affinity than FePB (Table 2). Because the primary Tyr/His-Fe(III) coordination framework is conserved, we interpret this difference as arising from modest changes in the binding interface beyond the first coordination sphere. Notably, the FePB^{v}-FatB structure reveals a structured water relay that fills the cavity generated by photodecarboxylation and introduces additional second-sphere hydrogen-bonding contacts linking the catecholates, Arg109, and the PB^{v} backbone. While our current dataset does not isolate this water relay as the sole causal determinant of the affinity difference, its presence provides a plausible structural basis for enhanced interfacial complementarity and for the reduced mutational sensitivity observed for FePB^{v} binding compared to FePB. We clarified this point in the Discussion section by more explicitly linking the FePB^{v} solvent-relay architecture to the higher apparent affinity of FePB^{v} relative to FePB, while keeping the causal interpretation appropriately qualified.

Original Text:

"[...] Arg109, and the PB^{v} backbone. Together, these results are consistent with a model in which FatB preserves high-affinity recognition of chemically modified ligands by coupling a conserved Tyr/His-Fe(III) core with potentially compensatory water-mediated second-shell interactions that maintain interfacial complementarity.

While apo-FatB retains the conserved class-III SBP fold, [...]"

Revised Text:

"[...] Arg109, and the PB^{v} backbone. This structural framework also provides a plausible basis for the higher observed affinity of FePB^{v} relative to FePB (Table 2). Because the Tyr317/His252-Fe(III) coordination framework is conserved, the affinity difference is more likely to arise from ligand-specific second-sphere interaction at the binding interface. This second-sphere interaction is manifested as a solvent relay that provides compensatory hydrogen-bonding contacts and enhances protein-ligand interaction network at the interface.

While apo-FatB retains the conserved class-III SBP fold, [...]"

Responses to the comments from Reviewer #3

In this manuscript, Lee et al. investigate the structural and mechanistic basis of ferric siderophore recognition by the Gram-positive substrate-binding protein FatB from Bacillus cereus. Using X-ray crystallography, SAXS, CD spectroscopy, mutagenesis, fluorescence binding assays, molecular dynamics simulations, and TD-DFT calculations, the authors characterize FatB in its apo form and in complex with ferric petrobactin (FePB), its ferric photoproduct, and a catecholate mimic.

The structures reveal that ferric ligand binding induces a conserved hinge-like domain closure characteristic of class-III SBPs. A central finding is that FatB actively remodels the Fe(III) coordination sphere: a protein-derived Tyr317/His252 pair directly coordinates the metal, replacing native ligand donors. In the photoproduct complex, a structured water network compensates for photodecarboxylation-induced ligand changes, preserving high-affinity binding. Mutational analysis supports ligand-dependent contributions of Tyr317 and His252, and spectroscopic/quantum-chemical analyses indicate that Tyr317 modulates the excited-state electronic structure of the bound ferric complex.

Overall, the study is thorough, and the authors support their claims with multiple experimental and computational techniques. However, the following issues should be addressed before the manuscript can be accepted for publication:
→ We sincerely appreciate the reviewer's insightful and accurate overview of our work. We also greatly appreciate the reviewer's insightful comments, which we address point-by-point below.

In lines 129-132 from the main text, the authors describe key conformational changes based on the superposition of apo-FatB and FePB-FatB. It is unclear how the uncertainty in the provided values was determined. Additionally, the value reported (8.2) in "a straightening of the central alpha-helix by 8.2 +/- 0.2 ..." differs from the value (8.3) depicted in Figure 2E.
→ We thank the reviewer's careful check of the values. We would like to clarify the distinction between the value shown in the figure and that in the main text. The value of 8.3° shown in Fig. 2e represents the specific straightening angle measured from the direct superposition of apo-FatB and the FePB-FatB complex, as this figure specifically illustrates the FePB-bound state as a representative example. In contrast, the value of 8.2 ± 0.2° reported in the main text represents the mean value calculated across all three ferric-ligand-bound FatB structures determined in this study (FePB-FatB, $FePB^{v}$-FatB, and $Fe(3,4\text{-}DHB)_2$-FatB). The uncertainty (± 0.2) corresponds to the standard deviation of these measurements, demonstrating the consistency of this conformational change regardless of the specific ferric ligand bound.
To avoid potential confusion for the readers, we have revised the caption of Fig. 2e to specify that it shows the value for the representative FePB-FatB complex, and we have added a sentence in the main text to clarify that the reported mean value encompasses all ferric-ligand-bound FatB complexes. We have also detailed this calculation in the legend of Supplementary Table 3. The revised or added texts are as follows.
The added texts in the figure caption of Fig. 2e:
"[...] change of the bend segment (residues 196–202). The 8.3° value shown corresponds to the direct apo-FatB/FePB-FatB superposition used for this representative panel."
The added sentence in the main text:

"[...] 3.0 ± 0.1 Å (Fig. 2d–f and Supplementary Table 3). Here, the reported values (mean ± standard deviation (SD)) were calculated across all three ferric-ligand-bound FatB complexes. These values place FatB [...]"
The added text in the legend of Supplementary Table 3:
"[...] residue 202 (helix terminus). In the main text, values are reported as mean ± SD across the three ferric-ligand-bound complexes (FePB-FatB, $FePB^{v}$-FatB, and $Fe(3,4\text{-DHB})_2$-FatB)."

The authors clearly define 'apo-FatB' as the ligand-free form. It would be helpful for a more general readership if the authors specified that 'holo' referred to the ligand-bound form (line 136; main text).
→ We appreciate the reviewer's constructive suggestion to clarify the terminology for a broader audience. To improve precision and avoid potential confusion, we decided to replace the term 'holo' with explicit descriptors throughout the manuscript. Importantly, our structural and SAXS data show that PB binding alone (PB-FatB) retains an open conformation similar to apo-FatB, whereas only ferric-ligand binding induces the pronounced open-to-closed transition. Therefore, using 'holo' as a general label for ligand-bound states could inadvertently group PB-FatB (open) with ferric-ligand-bound FatB complexes (closed). We now explicitly distinguish these states in the main text.
The relevant parts of the manuscript were revised as follows.
Original Text:
"This transition produces clear crystallographic compaction, quantified by superposing the N-terminal domain of apo-FatB onto each ligand-bound FatB complex."
Revised Text:
"This transition produces clear crystallographic compaction, quantified by superposing the N-terminal domain of apo-FatB onto each ferric-ligand-bound FatB complex."

Original Text:
"Despite the substantial transition from the apo to the holo forms, the three FatB complexes bound to ferric ligands adopt highly similar global structures. The N-terminal domains of any two complexes differ by only 0.22 ± 0.04 Å (RMSD), approximately one-third of the 0.61 ± 0.01 Å difference between the apo and any of the holo forms (Supplementary Table 4). This similarity extends to the entire protein, with an average all-atom RMSD of just 0.24 ± 0.02 Å among the ferric-ligand-bound FatB complexes compared to 1.75 ± 0.06 Å among the apo and holo structures (Supplementary Table 4)."
Revised Text:
"Despite the substantial transition from the open to the closed conformations, the three FatB complexes bound to ferric ligands adopt highly similar global structures. The N-terminal domains of any two complexes differ by only 0.22 ± 0.04 Å (RMSD), approximately one-third of the 0.61 ± 0.01 Å difference between the apo and any of the ferric-ligand-bound forms (Supplementary Table 4). This similarity extends to the entire protein, with an average all-atom RMSD of just 0.24 ± 0.02 Å among the FatB complexes with ferric ligands, compared to 1.75 ± 0.06 Å for apo-FatB relative to the ferric-ligand-bound FatB structures (Supplementary Table 4)."

The authors state a Guinier analysis was performed on the SAXS data in lines 160-162 of the main text. It would be helpful to include the Guinier plots in the supplementary information, as they are important for assessing quality of SAXS data.
→ We agree with the reviewer that Guinier plots are important for assessing the SAXS data quality. Accordingly, we have added a new Supplementary Figure (Supplementary Fig. 7) presenting the Guinier plots for all FatB states. Within the fitted low-q region satisfying $qR_g < 1.3$, the Guinier plots show linear behavior without low-q upturn. In addition, the symmetric, monodisperse peaks in the size-

exclusion chromatography profiles (Supplementary Fig. 8; formerly, Supplementary Fig. 6) further support sample monodispersity under the SAXS conditions. We updated the main text to reference these supporting data.
The following is the revised part of the manuscript according to the reviewer's comments.
Original Text:
"[...] by the SAXS-derived structural parameters (Table 1). Guinier analysis showed that the open states (apo- and PB-FatB) share a radius of gyration (R_g) of approximately 22.1 Å, whereas the ferric-ligand-bound FatB complexes are more compact, with smaller R_g values ranging from 21.0 to 21.4 Å[46]."
Revised Text:
"[...] by the SAXS-derived structural parameters (Table 1). To determine the radius of gyration (R_g), we performed Guinier analysis for all FatB states, and the corresponding Guinier plots are provided in Supplementary Fig. 7. The resulting Guinier plots show linear behavior in the fitted low-q region (qR_g < 1.3) without a low-q upturn, and the symmetric, monodisperse peaks observed in the size-exclusion chromatography profiles (Supplementary Fig. 8) are consistent with sample monodispersity under the SAXS conditions. Based on the Guinier analysis, the open states (apo- and PB-FatB) share an R_g of approximately 22.1 Å, whereas the ferric-ligand-bound FatB complexes are more compact, with smaller R_g values ranging from 21.0 to 21.4 Å[46]."

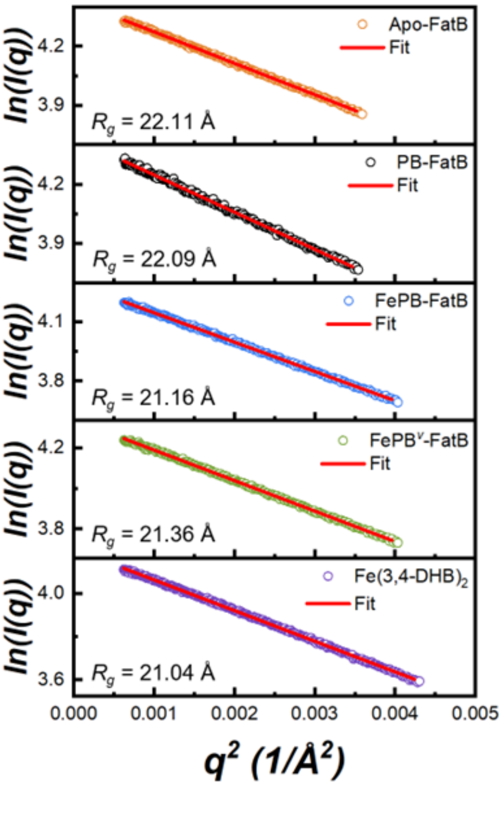

Supplementary Fig. 7. Guinier plots of apo- and ligand-bound FatB states. Guinier plots (*ln I*(*q*) vs q^2) are shown for apo-FatB, PB-FatB, FePB-FatB, FePB^v-FatB, and Fe(3,4-DHB)$_2$-FatB. Linear Guinier fits (red lines) were performed within the low-q region satisfying the standard Guinier criterion (qR_g < 1.3), and the resulting R_g values are reported in Table 1.

In figure 3, it would be helpful if the authors provided a difference in the experimental SAXS profiles for the apo and ligand bound FatB. This is not essential to include in the main text, but it would be beneficial for the reader to at least include in the supplementary information.

→ We agree with the reviewer that presenting difference SAXS profiles facilitates direct visual comparison between the apo and ligand-bound states. We therefore added a new figure (Supplementary Fig. 6) showing the difference SAXS curves relative to apo-FatB ($\Delta I(q) = I(q)_{ligand\text{-}bound\ FatB} - I(q)_{apo\text{-}FatB}$) for PB-FatB, FePB-FatB, FePB^{v}-FatB and Fe(3,4-DHB)$_2$-FatB. These curves provide an intuitive visualization consistent with our CRYSOL-based classification: PB-FatB remains close to apo-FatB, whereas ferric-ligand-bound FatB complexes show clear deviations.

The relevant part of the manuscript was revised as follows.

Original Text:

"[...] by their respective closed-state crystal models. The observation that PB-FatB retains [...]"

Revised Text:

"[...] by their respective closed-state crystal models. This separation is also evident directly in the experimental data: difference SAXS curves relative to apo-FatB ($\Delta I(q) = I(q)_{ligand\text{-}bound\ FatB} - I(q)_{apo\text{-}FatB}$) remain close to zero for PB-FatB within experimental uncertainty, whereas those of all ferric-ligand-bound FatB complexes show clear, systematic deviations (Supplementary Fig. 6). The observation that PB-FatB retains [...]"

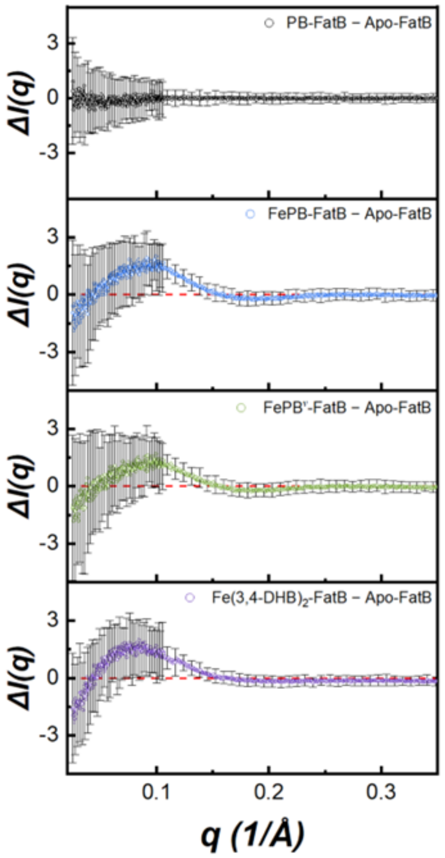

Supplementary Fig. 6. Difference SAXS profiles between apo-FatB and ligand-bound states. Difference SAXS profiles were calculated as $\Delta I(q) = I(q)_{ligand\text{-}bound\ FatB} - I(q)_{apo\text{-}FatB}$ using buffer-subtracted SAXS profiles placed on the same intensity scale (identical concentration normalization for all states). Scattering curves are shown for PB-FatB, FePB-FatB, FePB^{v}-FatB, and Fe(3,4-DHB)$_2$-FatB

relative to apo-FatB, with panels arranged from top to bottom in this order. Error bars represent propagated experimental uncertainties, and the red dashed line indicates $\Delta I(q) = 0$.

In the figure 3 caption (line 950), it should read "χ2" instead of "c2"
→ We thank the reviewer for noticing this typographical error. We have corrected "c2" to "χ^2" in the Fig. 3 caption. In addition, because this issue arose from symbol-font conversion, we systematically checked the entire manuscript (including figure legends and Supplementary Information) for similar Greek-character rendering errors and corrected them throughout.

Finally, for each state depicted in Figure 3, the authors present the corresponding DAMFILT bead model superimposed on the structure. It would be helpful for the authors to provide a justification for using the DAMFILT model, as this "averaged" model does not necessarily ensure that the theoretical SAXS profile calculated from the DAMFILT envelope matches the experimental SAXS data.
In ab initio SAXS reconstructions, two commonly reported models that directly fit the experimental data are the most-probable model (e.g., selected based on normalized spatial discrepancy from independent DAMMIF runs) and the DAMMIN-refined model. It would be more appropriate to compare these models with the crystal structures.
→ We thank the reviewer for this important point regarding the interpretation and reporting of our *ab initio* SAXS bead models. We acknowledge that our original presentation was not sufficiently clear. In Fig. 3a, the reported χ^2 values quantify the agreement between the experimental SAXS profiles and the theoretical scattering curves calculated from the crystal structures using CRYSOL; these χ^2 values do not originate from DAMFILT-derived envelopes. Accordingly, the residuals plotted beneath the scattering profiles represent the deviations between the experimental SAXS data and the CRYSOL curves calculated from the crystal structures, rather than any comparison to bead-model envelopes. In the original figure, DAMFILT was included solely as a consensus visualization of recurring shape features across independent *ab initio* reconstructions, rather than as the primary model for assessing agreement with the experimental SAXS data.

Following the reviewer's suggestion and common SAXS reporting practice, we have revised the manuscript to compare the crystal structures against bead models that directly originate from fitting the experimental scattering profiles. Specifically, for each FatB state, we performed 10 independent *ab initio* reconstructions using DAMMIF and enabled the built-in "Refine with DAMMIN" option, yielding a corresponding DAMMIN-refined bead model for each run. After completing the ensemble of reconstructions, DAMSUP was used to superpose the ensemble and to compute the pairwise NSD matrix. We used DAMSEL to select a representative model defined as the one with the smallest mean NSD within the ensemble. In the revised Fig. 3a and 3c, we now show the DAMMIN-refined bead models superimposed with the corresponding crystal structures, replacing the previous DAMFILT envelopes.

For transparency, we additionally provide a new Supplementary Figure (Supplementary Fig. 9) showing, for each FatB state, side-by-side overlays of the most-probable model with the smallest mean NSD (from DAMSEL) and the DAMMIN-refined model with the crystal structure, together with NSD statistics (mean ± SD with the NSD value of the selected model). We note that ab initio bead reconstructions are inherently non-unique and that refinement can alter bead-level details while optimizing the fit to the experimental scattering profile. Therefore, we do not over-interpret fine structural features of either bead model; instead, these additional overlays are provided to document the range of acceptable representations and to allow readers to directly assess the agreement between the solution envelopes and the corresponding crystal structures.

Consequently, our comparison in Supplementary Fig. 9 is intended to document the range of acceptable ab initio representations rather than to argue for the superiority of one specific reconstruction. Despite the inherent non-uniqueness of these models, the fact that both the most-probable and DAMMIN-refined envelopes consistently encompass the crystal structure validates the robustness of our structural interpretation. This dual reporting allows readers to directly assess the high degree of agreement between the solution-state envelopes and the crystallographic data, confirming that the FatB structure observed in the crystal lattice is representative of its conformation in solution.

The relevant parts of the manuscript were revised as follows.

Original Text in Methods:

"[...] using a software called GNOM[47]. To visualize the three-dimensional solution shape, *ab initio* bead modeling was performed using DAMMIF[48]. Ten independent models were generated assuming P1 symmetry, averaged using DAMAVER, and subsequently filtered using DAMFILT to produce the final representative envelope[49]. The alignment of the atomic crystal [...]"

Revised Text:

"[...] using a software called GNOM[47]. To visualize the three-dimensional solution shape, ab initio bead modeling was performed using DAMMIF[48]. Ten independent ab initio reconstructions were generated assuming P1 symmetry, and each reconstruction was further refined against the experimental scattering profile using DAMMIN (Refine with DAMMIN option)[48]. The resulting bead models were superposed using DAMSUP to calculate the pairwise normalized spatial discrepancy (NSD) matrix, and a representative reconstruction was selected using DAMSEL based on the smallest mean NSD within the ensemble[49]. The alignment of the atomic crystal [...]"

Original Text in Fig. 3 legend:

"[...] matches the experimental data. For every state, the corresponding DAMFILT *ab initio* bead model (gray) is shown with this best-fitting crystal structure superposed, and the residuals for each fit are plotted beneath the scattering profiles. (b) Pair-distance distribution function [...]"

Revised Text:

""[...] matches the experimental data. For every state, the corresponding DAMMIN-refined ab initio bead model (gray) is shown with this best-fitting crystal structure superposed, and the residuals from the CRYSOL fits to the experimental SAXS data are plotted beneath the scattering profiles. (**b**) Pair-distance distribution functions [...]"

Original Text in Fig. 3 legend:

"[...] (**c**) Superposition of the apo-FatB and FePB-FatB DAMFILT envelopes with their crystal structures, [...]"

Revised Text:

"(**c**) Superposition of the apo-FatB and FePB-FatB DAMMIN-refined bead models with their crystal structures, [...]"

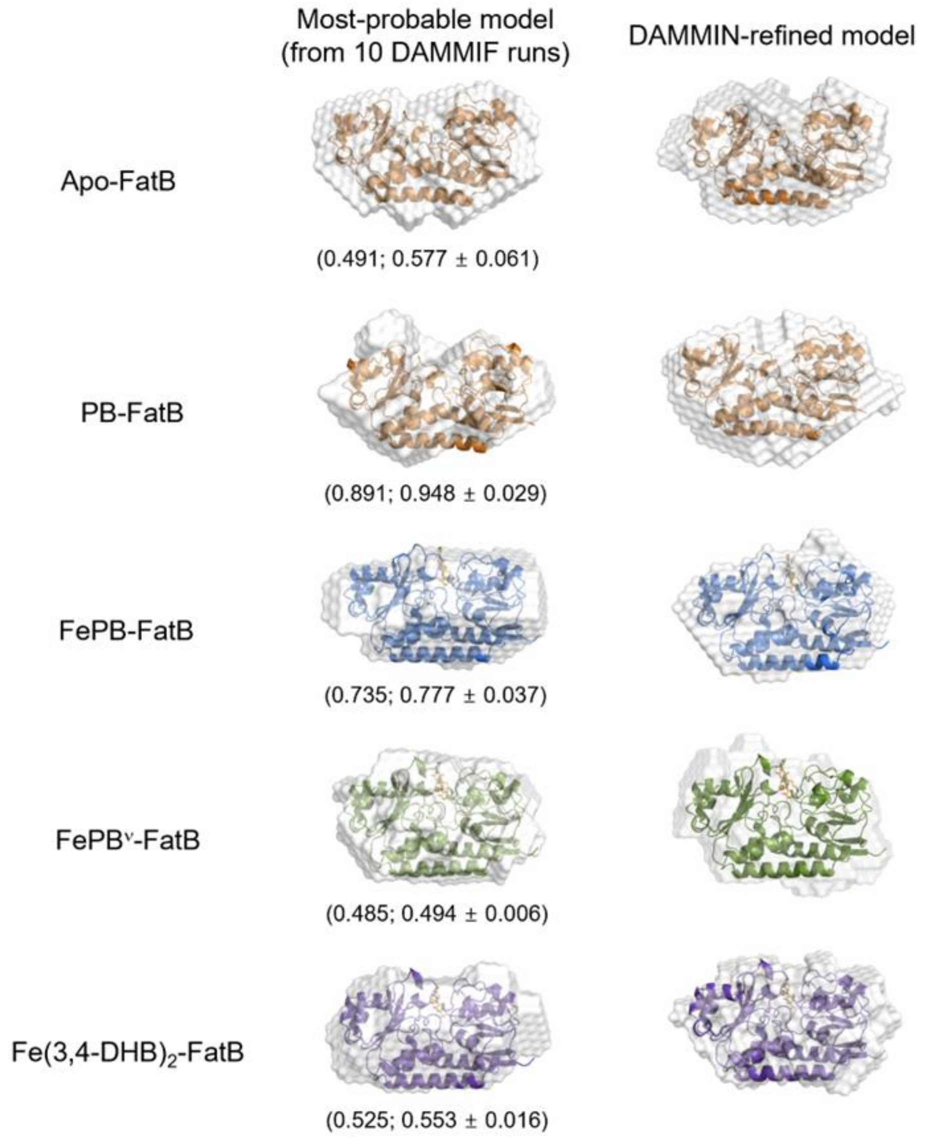

Supplementary Fig. 9. Most-probable and DAMMIN-refined ab initio bead models for FatB states. For each state (apo-FatB, PB-FatB, FePB-FatB, FePB^{v}-FatB, and Fe(3,4-DHB)$_2$-FatB), 10 independent ab initio reconstructions were generated using DAMMIF assuming P1 symmetry. (Left) the most-probable model (DAMSEL-selected) defined as the reconstruction with the smallest mean NSD after DAMSUP superposition of the ensemble. (Right) The corresponding DAMMIN-refined model obtained by refining each reconstruction against the experimental scattering profile (Refine with DAMMIN option). In each panel, the bead-model envelope is shown in gray and superposed with the crystal structure colored as apo-FatB (orange), FePB-FatB (blue), FePB^{v}-FatB (green), and Fe(3,4-DHB)$_2$-FatB (purple) (PB-FatB is superposed with the same best-matching crystal structure used for Fig. 3a). Numbers in parentheses report the NSD of the selected model and the ensemble mean ± SD. These additional overlays are provided to document the range of acceptable representations and to allow readers to directly assess the agreement between the solution envelopes and the corresponding crystal structures.
